# Carbonic Anhydrase Inhibition with Sulfonamides Incorporating Pyrazole- and Pyridazinecarboxamide Moieties Provides Examples of Isoform-Selective Inhibitors

**DOI:** 10.3390/molecules26227023

**Published:** 2021-11-20

**Authors:** Andrea Angeli, Victor Kartsev, Anthi Petrou, Mariana Pinteala, Volodymyr Brovarets, Roman Vydzhak, Svitlana Panchishin, Athina Geronikaki, Claudiu T. Supuran

**Affiliations:** 1 NeuroFarba Department, Sezione di Scienze Farmaceutiche, Università degli Studi di Firenze, Via Ugo Schiff 6, 50019 Sesto Fiorentino, Italy; andrea.angeli@unifi.it; 2 Centre of Advanced Research in Bionanoconjugates and Biopolymers, Petru Poni Institute of Macromolecular Chemistry, Aleea Grigore Ghica-Voda, no. 41A, 700487 Iasi, Romania; pinteala@icmpp.ro; 3 InterBioScreen, Chernogolovka, 142432 Moscow Region, Russia; vkartsev@ibscreen.chg.ru; 4 Department of Pharmacy, School of Health, Aristotle University of Thessaloniki, 54124 Thessaloniki, Greece; anthi.petrou.thessaloniki1@gmail.com; 5 Department of Chemistry of Bioactive Nitrogen-Containing Heterocyclic Bases, V.P. Kukhar Institute of Bioorganic Chemistry and Petrochemistry, NAS of Ukraine 1, Murmanska St, 02094 Kyiv, Ukraine; brovarets@bpci.kiev.ua (V.B.); rmvydzhak@gmail.com (R.V.); svpanch@ukr.net (S.P.)

**Keywords:** carbonic anhydrase, inhibitors, metalloenzymes, pyrazole derivatives

## Abstract

A series of benzenesulfonamides incorporating pyrazole- and pyridazinecarboxamides decorated with several bulky moieties has been obtained by original procedures. The new derivatives were investigated for the inhibition of four physiologically crucial human carbonic anhydrase (hCA, EC 4.2.2.1.1) isoforms, hCA I and II (cytosolic enzymes) as well as hCA IX and XII (transmembrane, tumor-associated isoforms). Examples of isoform-selective inhibitors were obtained for all four enzymes investigated here, and a computational approach was employed for explaining the observed selectivity, which may be useful in drug design approaches for obtaining inhibitors with pharmacological applications useful as antiglaucoma, diuretic, antitumor or anti-cerebral ischemia drugs.

## 1. Introduction

The proper function of physiological processes in the human body depends on the preservation of an adequate acid–base balance. Indeed, the normal intracellular pH range is between 7.35 and 7.45, but when the pH deviates from this range, pathological conditions are commonly observed [1]. CO_2_ is generated and used in many metabolic reactions, and one of the most important buffer systems used by cells is the homeostatic HCO_3_^−^/CO_2_ mechanism. Because of the slow reaction between CO_2_ and H_2_O, enzymes are fundamental to speed up the process; for example, carbonic anhydrases (CAs, EC 4.2.1.1) are efficient catalysts for the reversible reaction between CO_2_ and HCO_3_^−^ [2]. To date this class of enzymes has been divided into eight distinct and genetically unrelated families [3,4,5,6]. In humans, there are 15 isoforms, and their overexpression is often related to different diseases. Indeed, the abnormal expression of CA I, IV, IX, and XII isoforms in serum and synovium specimens are related to rheumatoid arthritis, and their overexpression also has been demonstrated to negatively affect cellular immunity processes and to enhance associated symptoms [7,8,9]. In addition, the overexpression of hCA IX and XII was observed in several cancer diseases, as well as in patients suffering from cerebral ischemia [3,10,11,12].

**Figure 1:**
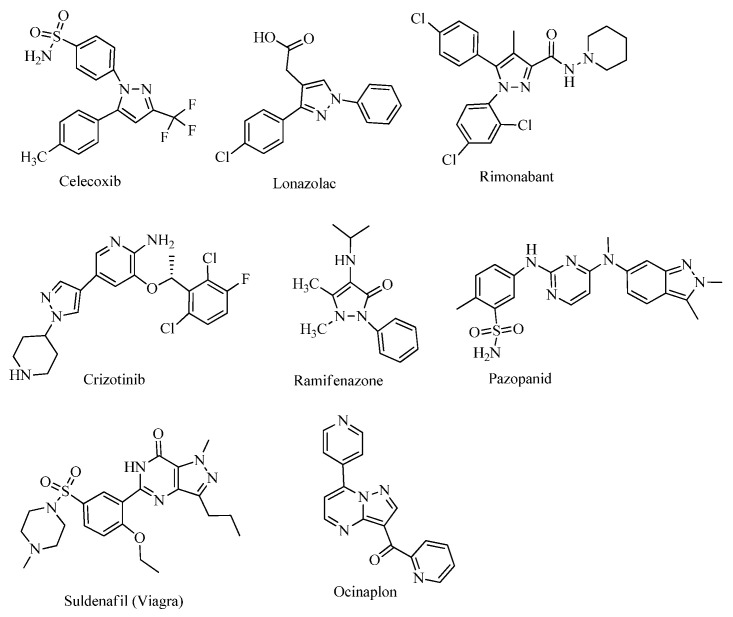
Structure of drugs bearing the pyrazole moiety.

In this context, the pyrazole scaffold is a versatile molecule that has attracted attention due to its wide range of diverse pharmacological activities, which make it a versatile lead molecule in several drug molecules such as celecoxib, ramifenazone, lonazolac, and rimonabant, drugs approved as COX-2 inhibitors [13,14,15,16], crizotinib [17] and paropanib [17] as anticancer drugs, sildenafil [17] (Viagra) PDE5 inhibitor, zometapine [18] as antidepressant, ocinaplon [19] as anxiolytic (Figure 1), and many others. Additionally, in recent years, their derivatives have been reported to possess antimicrobial activity [20,21,22] as well as antiviral [23,24], antidiabetic [25,26], anti-Alzheimer [27,28], antitubercular [29,30], and antileishmanial properties [31].

**Figure 2:**
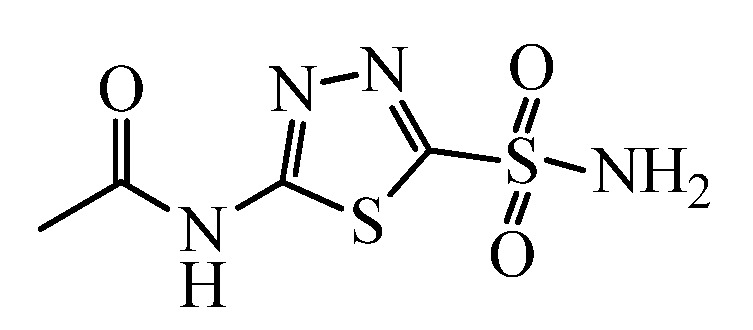
Structure of acetazolamide (**AAZ**).

Our aim was to further support our previous studies on hCAs as valid and robust pharmacological targets for the treatment of different pathological conditions that are characterized by the overexpression of different human CA isoforms such as CA IX and XII over the ubiquitous hCA I and hCA II in order to decrease the side effects due to their inhibition, such as with the clinically approved drug acetazolamide (**AAZ**) depicted in Figure 2.

## 2. Results

### 2.1. Chemistry

**Scheme 1:**
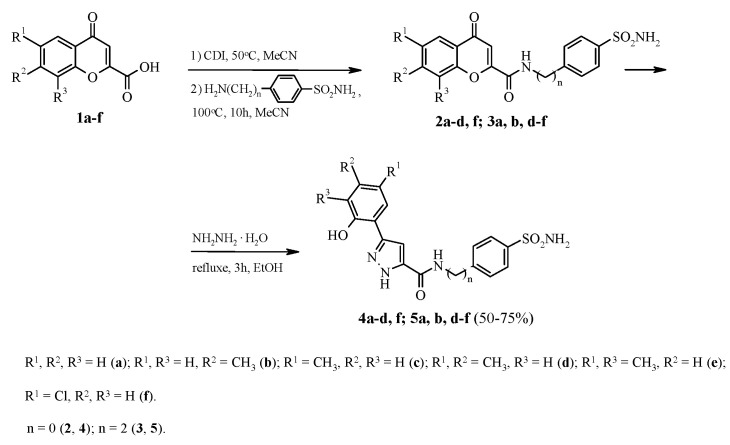
Synthesis of 3-(2-hydroxyaryl)-1H-pyrazole derivatives.

The synthesis of target compounds **4** and **5** is presented in Scheme 1. Starting 4-oxo-4H-chromene-2-carboxylic acids **1** [32,33] were initially converted into substituted amides **2** and **3**, which without further purification were used for the preparation of pyrazole derivatives **4a**–**d**,**f** and **5a**,**b**,**d**–**f**. Some examples of such transformations of 4-oxo-4H-chromene-2-carboxamides to substituted pyrazoles were also described earlier [34].

The structure and composition of compounds **4a**–**d**,**f** and **5a**,**b**,**d**–**f** were confirmed by ^1^H, ^13^C NMR spectroscopy and elemental analysis (see Supplementary Materials). The signals of all observed in the ^1^H NMR spectra protons appear in usual spectral regions that unambiguously correspond to the structure of the synthesized substances. For compound **5a** ^1^H NMR, spectra were recorded in solutions of DMSO-d_6_, CF_3_COOD and DMSO-d_6_ + 5% (mass) CF_3_SO_3_H. It should be noted that, obviously, compound **5a** predominantly exists as two tautomeric forms in the solution of DMSO-d_6_. In this case, a double set of signals of the OH group (13.78 s, 0.35H and 13.17 s, 0.65H), NH pyrazole fragment (10.39 s, 0.35H and 10.20 s, 0.65H) and NH carboxamide group (8.69 s, 0.35H and 8.13 s, 0.65H) was observed, while the signals of aromatic protons of hydroxyaryl substituents, 4-H pyrazole moiety and aliphatic groups appeared as broad multiplets and the signals of the arylsulfamide moiety were clearly separated and resolved at 7.28 (s, 2H, NH_2_).

In the ^1^H NMR spectra of compound **5a**, measured in CF_3_COOD, OH and NH proton signals were not detected; at the same time, the aromatic and aliphatic signals were more clearly separated and resolved, although some of their broadening was also observed. In the spectrum of compound **5a** measured in DMSO-d_6_ + 5% (mass) CF_3_SO_3_H, signals of protons of NH-pyrazole, hydroxy and aminosulfonyl groups were not detected, whereas the signals of aromatic and aliphatic protons were more clearly resolved and separated. Signals of aromatic protons of the arylsulfamide fragment were at 7.73 (d, *J* = 8.0 Hz, 2H, Ar) and 7.41 (d, *J* = 8.0 Hz, 2H, Ar). The signal of 4-pyrazole was superimposed with the signal of the orthohydroxyphenyl substituent at 7.24–7.11 ppm. At the same time, the NH signal of the carboxamide group of **5a** (8.45 s, 1H, NH) was clearly observable. In the NMR ^1^H spectra of compounds **4a**–**d**,**f** and **5b**,**d**–**f** recorded in DMSO-d_6_ and DMSO-d_6_ + 5% (mass) CF_3_SO_3_H, there was a set of signals similar to the corresponding set of signals in the NMR ^1^H spectra of compound **5a** measured in the same solvents. Measurement of the ^13^C NMR spectra of compound **5a** in DMSO-d_6_ or CF_3_COOD gave low-resolved or undefined peaks, while in spectra recorded in the solution of DMSO-d_6_ + 5% (mass) CF_3_SO_3_H, all the signals of carbon atoms (the number of corresponding to the number of carbon atoms in their structure) appeared as well-defined and highly resolved peaks.

Probably, the addition of a small amount of trifluoromethanesulfonic acid (as one of the strongest acids) is sufficient for the protonation of the pyrazole ring, which leads to an increase in the proton exchange rate, resulted in obtaining the more highly resolved and clearly defined spectra. Additionally, in the ^13^C NMR spectra, a quartet of trifluoromethyl group (clearly separated from the signals of the carbon atoms of compounds **4a**–**d**, **f** and **5a**, **b**, **d**–**f**) appeared. Molecular ion peaks [M+H]^+^ and [M−H]^−^ are usually detected in LC/MS spectra of the compounds **4**, **5**.

**Scheme 2:**
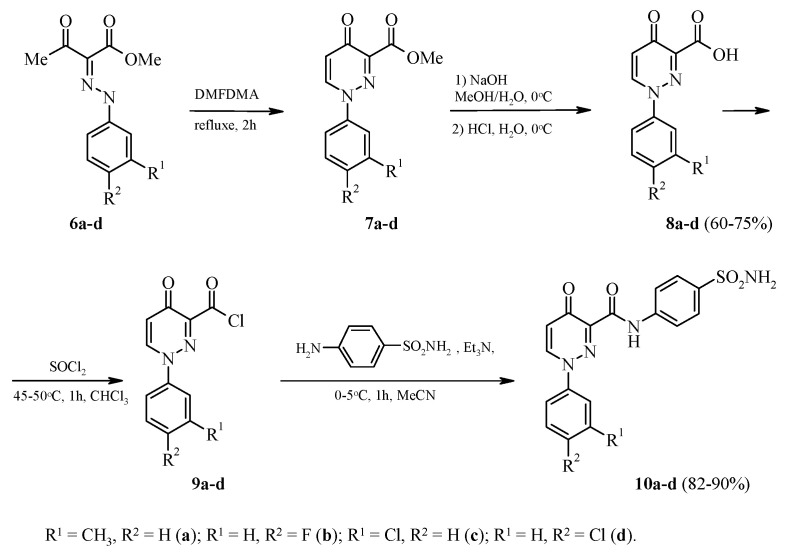
Synthesis of 1-aryl-4-oxo-1,4-dihydropyridazine derivatives.

N-[4-(aminosulfonyl) phenyl]-1-aryl-4-oxo-1,4-dihydropyridazine-3-carboxamides **10** were synthesized according to the pathway shown in Scheme 2. Methyl 4-oxo-1-aryl-1,4-dihydropyridazine-3-carboxylates **7** were obtained by refluxing the corresponding methyl 3-oxo-2-(arylhydrazono)butanoates **6** in DMFDMA [35,36,37], and their further hydrolysis led to 4-oxo-1-aryl-1,4-dihydropyridazine-3-carboxylic acids **8** [36,37]; then, **8** were converted into appropriate acid chlorides **9**; finally, treatment with aromatic amines yielded **10a**–**d**.

The structure and composition of compounds **8a**–**d** and **10a**–**d** were confirmed by ^1^H, ^13^C NMR spectroscopy and elemental analysis. The signals of the 1-aryl-4-oxo-1,4-dihydropyridazine moiety of compounds **8** and **10** were observed at: H-5 (d, *J* = 7.9 Hz) 6.90–7.05 ppm; and H-6 (d, *J* = 7.9 Hz) 7.95–8.10 ppm. The carboxamide group signals of compounds **10a**–**d** appeared as a singlet at 11.90–12.15 ppm, whereas the signals of the sulfonamide groups appeared as a singlet at 7.30–7.35 ppm. 

It should be mentioned that N-[4-(aminosulfonyl)phenyl]-1-(4-chlorophenyl)-4-oxo-1,4-dihydropyridazine-3-carboxamide **10d** was obtained earlier by another method [38]. The proton signal data in the ^1^H NMR spectra of obtained compound **10d** did not match the data described in [38], while all ^1^H NMR spectral data of the acids **8a**–**d** and amides **10a**–**d** correlated with ^1^H NMR spectral data of the similar compounds described in [35,36,37,39,40]. Due to this, probably, the structure of **10d** presented in [38] is not correct. The number of signals in the ^13^C NMR spectra of compounds **10a**–**d** corresponds to the number of carbon atoms in their structures, whereas the signals of carbon atoms of 4-fluorophenyl substituent in **10b** appeared as doublets. Molecular ion peaks [M+H]^+^ and [M−H]^−^ are usually detected in LC/MS spectra of the compounds **10a**–**d**.

**Scheme 3:**
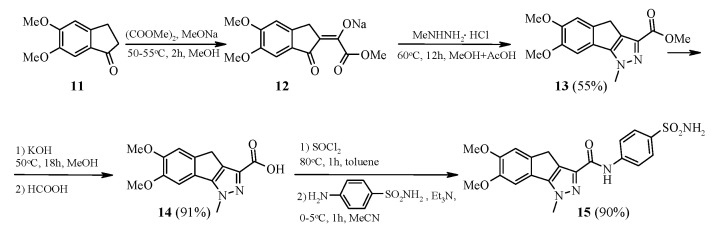
Synthesis of 6,7-dimethoxy-1-methyl-1,4-dihydroindeno[1,2-c]pyrazole derivatives.

N-[4-(Aminosulfonyl)phenyl]-6,7-dimethoxy-1-methyl-1,4-dihydroindeno[1,2-c]pyrazole-3-carboxamide **15** was obtained according to the pathway shown in Scheme 3. Compound **12** prepared by condensation reaction of 5,6-dimethoxyindan-2-one with dimethyloxalate in the presence of sodium methylate was used without additional purification to obtain 6,7-dimethoxy-1-methyl-1,4-dihydroindeno[1,2-c]pyrazole-3-carboxylate **13**, hydrolysis of which led to acid **14**. It should be also noted that some similar derivatives of 1-methyl-1,4-dihydroindeno[1,2-c]pyrazoles were described earlier [41]. Then, 6,7-dimethoxy-1-methyl-1,4-dihydroindeno[1,2-c]pyrazole-3-carboxylic acid **14** was converted into acid chloride, which, without further purification, was transformed to **15**.

The structure and composition of compounds **13**–**15** were confirmed by ^1^H, ^13^C NMR spectroscopy and elemental analysis. The signals of all present protons were observed in the ^1^H NMR spectra of N-[4-(aminosulfonyl)phenyl]-6,7-dimethoxy-1-methyl-1,4-dihydroindeno[1,2-c]pyrazole-3-carboxamide **15**. The NH proton signal was found as singlet at 10.36 ppm, and the NCH_3_ group signal and signals of two methoxy groups as a singlets at 4.18, 3.85, 3.79 ppm. The signal of the methyl group appeared as a singlet at 3.62 ppm, while aromatic proton signals were observed as singlet (7.34 ppm) and doublets (8.02, *J* = 8.5 Hz and 7.76, *J* = 8.5 Hz). The number of signals in the ^13^C NMR spectra of compounds **13**–**15** corresponded to the number of carbon atoms in their structures. Molecular ion peaks [M+H]^+^ and [M−H]^−^ are usually detected in LC/MS spectra of compound **15**.

### 2.2. Biological Evaluation

All synthesized compounds were evaluated for their inhibitory activity against four human CA isoforms, which are: hCA I, hCA II, hCA IX and hCA XII (Table 1).

The inhibition of hCA I spanning among nanomolar to micromolar ranges, in particular, eight compounds **4b**, **4c**, **4d**, **4f**, **5d**, **5e**, **10a** and **10c** (Table 1), exhibited higher activity than reference drug acetozolamide (Ki 250 nM). The best activity among them was achieved for compound **10d** with K_i_ 6.2 nM, followed by compound **5e** (K_i_ 71.4nM). The lowest activity was shown by compound 4a with K_i_ at 3822 nM. The order of activity of these compounds against hCA I can be presented as follows: **10d** > **5e** > **5a** > **4b** > **4f** > **10a** > **5d** > **4c** > **5b** > **10b** > **15** > **10c** > **4d** > **5f** > **4a**.

According to structure–activity relationships, the presence of [(4-sulfamoylphenyl)amino]carbonyl substitute at position 3 of 1-(4-chlorophenyl)pyridazin-4(1H)-one (**10d**) is beneficial for hCA I inhibitory activity. The order of activity of phenyl-substituted pyridazine-4(1H)-one derivatives can be presented as **10d** > **10a** > **10b** > **10c**. Thus, replacement of 4-chlorobenzene by 3-tolyl resulted in compound **10a** with much lower activity than **10d,** followed by 4-fluoro- (**10b**) and 3-chlorobenzene derivatives (**10c**). The replacement of pyridazine derivative **10d** by 3-(2-hydroxy-3,5-dimethylphenyl)-N-(4-sulfamoylphenethyl)-1H-pyrazole-5-carboxamide (**5e**) decreased some activity. Removing both methyl groups from **5e** led to less-active compound (**5a**) compared with the previous one. Introduction of 2-hydroxy-4-methylbenzene to position 3 of pyrazole moiety as well as [(4-sulfamoylphenyl)amino]carbonyl substitute at position 5 (**4b**) further decreased the activity against hCA I, but this was still among the active compounds. The presence of 2-hydroxyphenyl substituent at position 3 of pyrazole ring and [N-(4-aminosulfonyl)phenyl]-5-carboxamide (**4a**) appeared to be detrimental. It is interesting to notice that for [4-(aminosulfonyl)phenyl]ethyl-1H-pyrazole carboxamides (compounds **5a**–**5f**), the presence of 2-OH and 3,5 dimethyl substitution of phenyl ring, respectively, is very important for hCA I inhibitory activity, while for [4-(aminosulfonyl)phenyl]-1H-pyrazole-5-carboxamides (**4a**–**4f**) a positive role is played by the presence of 2-OH, 4-Me substitution of phenyl ring. The K_i_ values against cytosolic hCA II isoform were in the range of 3.3 to 866.7 nM, and the order of activity was **15** > **10a** > **10d** > **5f** > **4d** > **5b** = **5e** > **5d** > **5a** > **4f** > **10b** > **10c** > **4c** > **4b** > **4a**. Compound **15** displayed the highest activity among others against hCA II, with Ki at 3.3 nM compared to AAZ (Ki at 12.1 nM). Furthermore, this compound was the most selective one, with a selectivity index (SI) of 219.9 towards hCA I, 2.0 compared to hCA IX and 24.4 towards hCA XII isoforms. Four compounds displayed higher activity against cytosolic hCA II isoform than reference drug **AAZ**.

**Figure 3:**
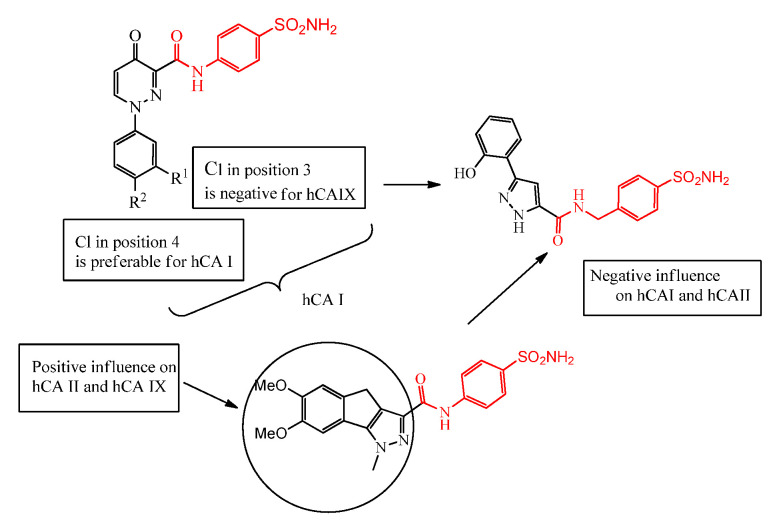
Structure–activity relationship summary of tested compounds.

The structure–activity relationship studies revealed that the presence of 5,6-dimethoxy-2,3-dihydro-1H-indene fused to pyrazole ring (**15**) was favorable for inhibitory activity towards hCA II isoform. Replacement of 6,7-dimethoxy-1-methyl-N-(4-sulfamoylphenyl)-1,4-dihydroindeno[1,2-c]pyrazole-3-carboxamide by 4-oxo-N-(4-sulfamoylphenyl)-1-(m-tolyl)-1,4-dihydropyridazine-3-carboxamide yielded the somewhat less active compound **10a**. Replacement of m-tolyl group by 4-chlorophenyl (**10d**) decreased the activity further. The 3-(5-chloro-2-hydroxyphenyl)-N-(4-sulfamoylphenethyl)-1H-pyrazole-5-carboxamide appeared to be less active (**5f**) compared to compound **10d**. Finally, among all compounds tested, the group (**5a**–**f**) appeared to be the more active compared to **4a**–**f** and **10a**–**d**. Thus, for compounds **5a**–**f**, the most favorable was the presence of 5-chloro-2-hydroxyphenyl substitution at position 3 of pyrazole moiety of N-{2-[4-(aminosulfonyl)phenyl]ethyl}-3-(5-chloro-2-hydroxyphenyl)-1H-pyrazole-5-carboxamide (**5f**), while the presence of 2-hydroxy substituent (**5a**) had the most negative influence on hCA II inhibitory activity. On the contrary, this compound was among the active compounds (third position in order of activity) against hCA I isoform. As far as [(4-aminosulfonyl) phenyl] 3-substituted phenyl-1H-pyrazole-5-carboxamide derivatives are concerned, the most beneficial was the 2-hydroxy-4,5-dimethylphenyl substitution at position 3 of the pyrazole moiety. Replacement of 2-hydroxy-4,5-dimethylphenyl group by 2-hydroxy-5-chlorophenyl led to less active compound **5b**, which was equipotent with compound **5e** bearing 2-hydroxy-3,5-dimethoxyphenyl substituent. Removal of both methyl groups was detrimental to the hCA II inhibitory activity, leading to the less active compound (**4a**) in this group. Finally, as regards the N-[4-(aminosulfonyl)phenyl]-1-aryl-4-oxo-1,4-dihydropyridazine-3-carboxamides (**10a**–**10d**), the most favorable for inhibitory activity towards hCA II appeared to be 3-methylphenyl substituent, while 3-chlorophenyl had a negative effect on activity. As regards the activity towards hCA IX isoform, the activity order of the compounds could be presented as follows: **15 > 4c > 5b > 4f > 10a > 5e > 5d > 4b > 5f > 5a > 10b > 4d > 10d > 4a > 10c.** The Ki values were in range of 6.1 to 568.8nM. The best activity was again observed for compound **15,** as in case of the hCA II isoform, with Ki at 6.1 nM, followed by compound **4c** (K_i_ 8.5 nM. Three compounds (**4c, 5b** and **15**) showed activity towards the hCA IX isoform better than AAZ (Ki at 25.8 nM). The lowest activity was shown by compound **10c** with Ki at 568.8nM. According to the structure–activity relationships, the presence of 5,6-dimethoxy-2,3-dihydro-1H-indene fused to pyrazole ring (**15**), as in the case of hCA II, was beneficial for hCA IX inhibitory activity. Among three groups of compounds, it seem that 3-(2-hydroxyaryl)-1H-pyrazole-5-carboxamide **4a**–**d**,**f** and **5a**,**b**,**d**–**f** were more potent. Thus, for group of **4a**–**d**,**f** the presence of 2-hydroxy-5-methylphenyl substituent at position 3 of pyrazole moiety and N-[4-(aminosulfonyl) phenyl carboxamide at the 5th position (**4c**) were positive for hCA IX inhibitory activity. Replacement of this substituent by 5-chloro-2-hydroxyphenyl (**4f**) decreased activity slightly, being in position 4 of the activity order, while removal of 5-Cl substituent was detrimental. In the group of N-{2-[4-(aminosulfonyl)phenyl]ethyl substituted phenyl-1H-pyrazole carboxamides, the most favorable substitution appeared to be 5-chloro-2-hydroxyphenyl (**5f**), followed by 2-hydroxy-3,5-dimethylphenyl (**5e**) with slightly lower activity. 2-Hydroxyphenyl substitution, as in the case of the previous group of compounds, had negative effect on hCA IX inhibitory activity. Unfortunately, these compounds showed moderate to weak inhibitory activity, with K_i_ in the range of 61.3–432.8 nM compared to the 25.7 nM of acetozolamide towards the hCA XII isoform. The summary of SAR is presented in Figure 3.

### 2.3. Molecular Docking Studies

In order to predict the possible mechanism of inhibition of the tested compounds, molecular docking studies were performed on the most active compounds **5d**, **5e**, **10d** and **15** as representative of the whole set of compounds. Human CAs isoforms have analogous active sites containing His94, His96 and His119 as conserved residues, which act as zinc ligands, and conserved residues Thr199 and Glu105, which act as ‘’gate keepers’’ [42,43,44,45]. Nevertheless, these isoforms differ in the residues mostly in the middle and to the exit of the active site cavity. Table 2 presents the results of the molecular docking studies of the tested compounds on hCA I, II, IX and XII isoforms. According to the docking results, all tested compounds bind the enzymes, chelating the Zn (II) ion, in a deprotonated form, as anions (negative nitrogen of the sulfonamide group) [46].

**Figure 4:**
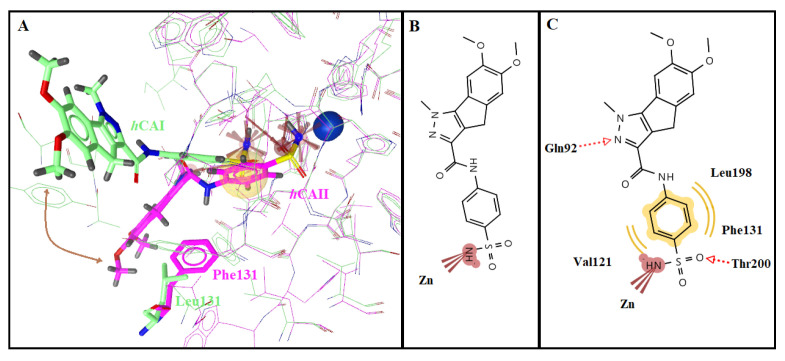
(**A**) Superposition of compound **15** bound to hCA I (green) in comparison to hCA II (magenta), with specific residues labeled. (**B**) 2D interaction diagram of compound **15** docking pose interactions with the key amino acids in hCA I, (**C**) in hCA II. Active site zinc shown as blue sphere; red dotted arrows indicate H-bond, and yellow spheres hydrophobic interactions. Orange double-headed arrow indicates the direction of conformational change of the compound bound to hCA I in comparison to hCA II enzyme.

Docking studies revealed that the selectivity profile as well as the inhibition mode of some compounds to each isoform depend on the variances in the active sites of the enzymes. More precisely, the nature of the amino acids of the active site of the enzymes affects the inhibition profile of the compounds because they play an important role in the final conformation adopted and interactions formed by compounds within the enzyme active site. For example, compound **15**, with K_i_ value for hCA I enzyme of 725.6 nM and K_i_ value for hCA II of 3.3 nM, adopts a different conformation when binding both hCAs. This is probably due to the presence of the hydrophobic residue Phe131 in hCA II enzyme in contrast to the minor residue Leu131 in hCA I. Despite the fact that this smaller residue in the hCA I enzyme allows ligands to freely enter the active site of the enzyme in compound **15** with a bulky part, it is not favorable. On the other hand, residue Phe131 interacts hydrophobically with compound **15**, increasing the enzyme–ligand interactions and consequently the inhibition and selectivity of the compound to this isoform (Figure 4A). Furthermore, compound **15** forms a hydrogen bond between the sulfonamide and the backbone of Thr200 to both isoforms and another H-bond between N-atom and residue Gln92 to isoform hCA II, which further stabilizes the complex and explains the high inhibition potency (Figure 4B,C).

**Figure 5:**
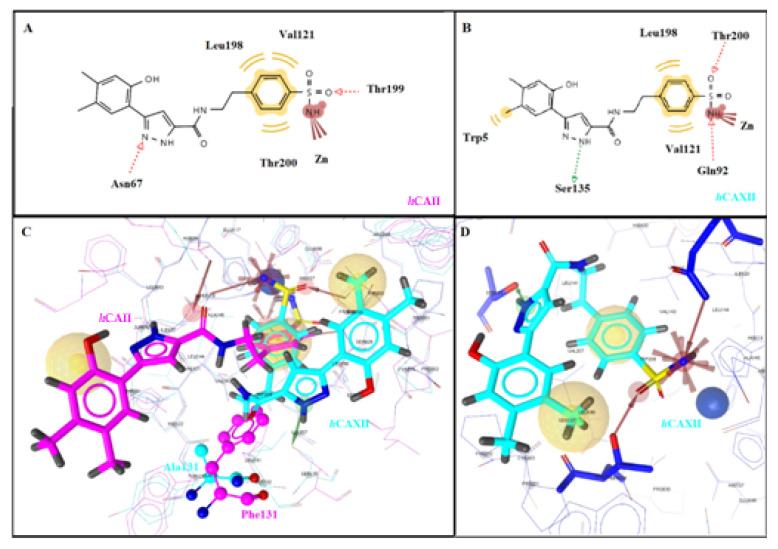
(**A**) 2D interaction diagram of compound **5d** docking pose interactions with the key amino acids in hCA II, (**B**) in hCA XII. (**C**) Superposition of compound **5d** bound to hCA II (magenta) in comparison to hCA XII (blue), with specific residues labeled. (**D**) 3D diagram of compound **5d** bound to hCA XII. Active site zinc shown as blue sphere, red dotted and green arrows indicate H-bond and yellow spheres hydrophobic interactions.

On the other hand, compound **5d** differs from compound **15** by the presence of an ethyl -longer chain. This longer chain provides flexibility to the compound, and enables it to avoid the steric hindrance of the bulky residue Phe131 hCA II isoform, increasing the inhibition potency. This is illustrated in Figure 5 where compound **5d** in both hCA II and hCA XII isoforms adopts a conformation that favors the interactions with both active sites of the isoforms, increasing the stability of the complex and the inhibition potency (Figure 5C,D). In both structures, the negative nitrogen of the sulphonamide group chelates the Zn (II) ion and forms hydrogen bonds. In isoform hCA I, the one oxygen atom of the sulphonamide group forms a hydrogen bond with residue Thr199, while in isoform hCA XII, it forms two hydrogen bonds with residues Thr200 and Thr199. Moreover, in isoform hCA XII, the N atom of heterocycle ring forms another H-bond with residue Ser135. Additionally, the benzene moiety interacts hydrophobically with residues Val121 and Leu198 (Figure 5A,B).

**Figure 6:**
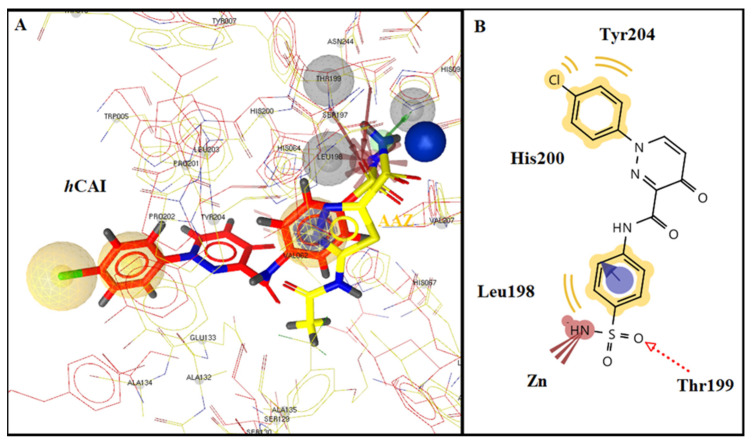
(**A**) Superposition of compound **10d** bound to hCA I (orange) in comparison to **AAZ** (yellow) in hCA I. (**B**) 2D interaction diagram of compound **10d** docking pose interactions with the key amino acids in hCA I. Active site zinc shown as blue sphere, red dotted arrows indicate H-bond, and yellow spheres indicate hydrophobic interactions.

The docking pose of compound **10d** into the active site of hCA I isoform revealed the probable reason for its high inhibition profile. As illustrated in Figure 6, compound **10d** binds hCA I in the same manner as **AAZ**, with the negative nitrogen of the sulphonamide group chelating the Zn (II) ion. However, the benzene moiety of the compound additionally interacts hydrophobically with residues His200 and Tyr204, increasing the stability of the enzyme–compound complex, probably explaining its lower K_i_ value in accordance with that of reference drug **AAZ**.

## 3. Materials and Methods

All used solvents were of analytical grade. The ^1^H and ^13^C spectra were recorded at 298 K on a Bruker AVANCE DRX-500 spectrometer (Rheinstetten, Germany) (at 500 and 125 MHz) in solutions of (TMS as internal reference): DMSO-*d_6_*, CF_3_COOD and DMSO-*d_6_* + 5%(mass)CF_3_SO_3_H. Chemical shifts (δ) are reported in ppm, and coupling constants (J) in Hz. Chemical ionization at atmospheric pressure mass spectra (APCI) were measured with an Agilent 1200 LC/MSD SL system (Waldbronn, Germany) equipped with DAD/ELSD/LSMS-6120 diode matrix and mass-selective detector, scan range *m*/*z* 80–1000. Melting points were determined in a Fischer-Johns melting point apparatus (Pittsburgh, USA) and are uncorrected. Elemental analysis was carried out in the Analytical Laboratory of the Institute of Bioorganic and Petrochemistry of the National Academy of Sciences of Ukraine by manual methods: the carbon and hydrogen contents were determined using the Pregl gravimetric method, nitrogen was determined using the Duma’s gasometrical micromethod, and sulfur was determined by the Scheininger titrimetric method.

### 3.1. General Procedure for the Synthesis of 3-(2-Hydroxyaryl)-1H-pyrazole-5-carboxamide 4**a**–**d**, **f** and 5**a**, **b**, **d**–**f**

To a solution of 2.5 mmol of the corresponding 4-oxo-4*H*-chromene-2-carboxylic acid 1 in 15 mL of acetonitrile, 2.5 mmol of CDI was added, and the mixture was heated at 50 °C until completion of carbon dioxide evolution. After cooling to ambient temperature, 0.374 g (2.2 mmol) of 4-aminobenzenesulfonamide or 0.44 g (2.2 mmol) 4-(2-aminoethyl)benzenesulfonamide was added to reaction mixture, which was then heated in a sealed vial for 10 h at 100 °C. After cooling to ambient temperature, the mixture was evaporated to dryness under reduced pressure. The residue was treated with 15 ml of 5% water sodium bicarbonate solution and stirred for 2 h on an ultrasonic stirrer at cooling (0–5 °C). The formed precipitate was filtered off, washed with water (10 mL) and then with ethanol (5 mL), and suspended in 15 mL of ethanol. Then, 0.63 g (6 mmol) of hydrazine hydrate was added to this suspension while stirring and the mixture was allowed to reflux for 3 h. After the cooling to ambient temperature, the resulted solution was left for 12 h, then the formed precipitate was filtered off, washed with water (10 mL), then ethanol (5 mL) and finally crystallized with DMF-ethanol mixture to yield **4a**–**d**,**f** and **5a**, **b**, **d**–**f** as colorless solids.

*N-[4-(Aminosulfonyl)phenyl]-3-(2-hydroxyphenyl)-1H-pyrazole-5-carboxamide* (**4a**). Yield 62%; m.p.325–327 °C. ^1^H-NMR (500 MHz, DMSO-*d*_6_ + 5%(mass)CF_3_SO_3_H) δ: 10.33 (s, 1H, NH), 7.97 (d, *J* = 8.5 Hz, 2H, Ar), 7.79 (d, *J* = 8.5 Hz, 2H, Ar), 7.68 (d, *J* = 7.7 Hz, 1H, Ar), 7.33 (s, 1H, Ar), 7.22–7.16 (m, 1H, Ar), 6.98 (d, *J* = 8.0 Hz, 1H, Ar), 6.92–6.86 (m, 1H, Ar). ^13^C-NMR (125 MHz, DMSO-*d*_6_ + 5%(mass)CF_3_SO_3_H) δ: 160.41, 154.78, 144.61, 143.78, 142.14, 138.96, 130.08, 127.93, 126.94, 120.27, 119.85, 116.82, 116.28, 105.62. MS (APCI): *m*/*z* = 359.0 [M+H]^+^; *m*/*z* = 357.0 [M–H]^.^ Anal.: Calcd. for C_16_H_14_N_4_O_4_S (%): C, 53.62; H, 3.94; N, 15.63; S, 8.95. Found: C, 53.51; H, 4.02; N, 15.70; S, 9.10.

*N-[4-(Aminosulfonyl)phenyl]-3-(2-hydroxy-4-methylphenyl)-1H-pyrazole-5-carboxamide* (**4b**). Yield 54%; m.p. 326–328 °C. ^1^H-NMR (500 MHz, DMSO-*d*_6_ + 5%(mass)CF_3_SO_3_H) δ: 10.40 (s, 1H, NH), 7.98 (d, *J* = 8.5 Hz, 2H, Ar), 7.79 (d, *J* = 8.5 Hz, 2H, Ar), 7.56 (d, *J* = 7.9 Hz, 1H, Ar), 7.29 (s, 1H, Ar), 6.71 (d, *J* = 7.9 Hz, 1H, Ar), 2.22 (s, 3H, CH_3_). ^13^C-NMR (125 MHz, DMSO-*d*_6_ + 5%(mass)CF_3_SO_3_H) δ: 159.97, 154.25, 144.18, 143.41, 141.70, 139.48, 138.52, 127.29, 126.50, 120.30, 119.80, 116.77, 113.06, 104.75, 20.79. MS (APCI): *m*/*z* = 373.2 [M+H]^+^; *m*/*z* = 371.0 [M–H]^−^ Anal.: Calcd. for C_17_H_16_N_4_O_4_S (%): C, 54.83; H, 4.33; N, 15.04; S, 8.61. Found: C, 54.90; H, 4.28; N, 14.87; S, 8.79.

*N-[4-(Aminosulfonyl)phenyl]-3-(2-hydroxy-5-methylphenyl)-1H-pyrazole-5-carboxamide* (**4c**). Yield 71%; m.p. 297–299 °C. ^1^H-NMR (500 MHz, DMSO-*d*_6_ + 5%(mass)CF_3_SO_3_H) δ: 10.41 (s, 1H, NH), 7.98 (d, *J* = 8.7 Hz, 2H, Ar), 7.78 (d, *J* = 8.7 Hz, 2H, Ar), 7.51 (d, *J* = 2.2 Hz, 1H, Ar), 7.32 (s, 1H, Ar), 7.00 (dd, *J* = 8.2 Hz, *J* = 2.2 Hz, 1H, Ar), 6.87 (d, *J* = 8.2 Hz, 1H, Ar), 2.23 (s, 3H, CH_3_). ^13^C-NMR (125 MHz, DMSO-*d*_6_ + 5%(mass)CF_3_SO_3_H) δ: 160.49, 152.57, 144.72, 143.76, 142.18, 138.95, 130.54, 128.36, 128.04, 126.93, 120.22, 116.71, 115.90, 105.59, 20.45. MS (APCI): *m*/*z* = 373.0 [M+H]^+^; *m*/*z* = 371.0 [M–H]^−^ Anal.: Calcd. for C_17_H_16_N_4_O_4_S (%): C, 54.83; H, 4.33; N, 15.04; S, 8.61. Found: C, 54.95; H, 4,40; N, 14.91; S, 8.82.

*N-[4-(Aminosulfonyl)phenyl]-3-(2-hydroxy-4,5-dimethylphenyl)-1H-pyrazole-5-carboxamide* (**4d**). Yield 67%; m.p. 272–274 °C. ^1^H-NMR (500 MHz, DMSO-*d*_6_ + 5%(mass)CF_3_SO_3_H) δ: 10.33 (s, 1H, NH), 7.96 (d, *J* = 8.7 Hz, 2H, Ar), 7.77 (d, *J* = 8.7 Hz, 2H, Ar), 7.44 (s, 1H, Ar), 7.27 (s, 1H, Ar), 6.75 (s, 1H, Ar), 2.16–2.12 (m, 6H, 2CH_3_). ^13^C-NMR (125 MHz, DMSO-*d*_6_ + 5%(mass)CF_3_SO_3_H) δ: 160.40, 152.67, 144.62, 143.85, 142.14, 138.94, 138.42, 128.43, 127.32, 126.92, 120.21, 117.19, 113.33, 105.15, 19.73, 18.76. MS (APCI): *m*/*z* = 387.0 [M+H]^+^; *m*/*z* = 385.0 [M–H]^−^ Anal.: Calcd. for C_18_H_18_N_4_O_4_S (%): C, 55.95; H, 4.70; N, 14.50; S, 8.30. Found: C, 55.79; H, 4.73; N, 14.35; S, 8.47.

*N-[4-(Aminosulfonyl)phenyl]-3-(5-chloro-2-hydroxyphenyl)-1H-pyrazole-5-carboxamide* (**4f**). Yield 75%; m.p. 302–304 °C. ^1^H-NMR (500 MHz, DMSO-*d*_6_ + 5%(mass)CF_3_SO_3_H) δ: 10.45 (s, 1H, NH), 7.98 (d, *J* = 8.8 Hz, 2H, Ar), 7.82–7.75 (m, 3H, Ar), 7.42 (s, 1H, Ar), 7.22 (dd, *J* = 8.7 Hz, *J* = 2.7 Hz, 1H, Ar), 6.99 (d, *J* = 8.7 Hz, 1H, Ar). ^13^C-NMR (125 MHz, DMSO-*d*_6_ + 5%(mass)CF_3_SO_3_H) δ: 160.26, 153.67, 144.46, 142.56, 142.15, 139.00, 129.37, 126.97, 126.93, 123.40, 120.26, 118.51, 118.35, 106.49. MS (APCI): *m*/*z* = 395.0([M(^37^Cl)+H]^+^; 30); *m*/*z* = 392.9([M(^35^Cl)+H]^+^, 100); *m*/*z* = 393.0 ([M(^37^Cl)–H]^−^, 30); *m*/*z* = 390.9 ([M(^35^Cl)–H]^−^, 100). Anal.: Calcd. for C_16_H_13_ClN_4_O_4_S (%): C, 48.92; H, 3.34; N, 14.26; S, 8.16. Found: C, 48.81; H, 3.38; N, 14.11; S, 8.33.

*N-{2-[4-(Aminosulfonyl)phenyl]ethyl}-3-(2-hydroxyphenyl)-1H-pyrazole-5-carboxamide* (**5a**). Yield 70%; m.p. 252–253 °C. ^1^H-NMR (500 MHz, DMSO-*d*_6_) δ: 13.78 (s, 0.351H, OH), 13.17 (s, 0.65H, OH), 10.39 (s, 0.35H, NH), 10.20 (s, 0.65H, NH), 8.69 (s, 0.35H, NH), 8.13 (s, 0.65H, NH), 7.76 (d, *J* = 7.9 Hz, 2H, Ar), 7.71–7.59 (m, 1H, Ar), 7.44 (d, *J* = 7.9 Hz, 2H, Ar), 7.28 (s, 2H, NH_2_), 7.24–7.16 (m, 1H, Ar), 7.11–6.90 (m, 2H, Ar), 6.94–6.86 (m, 1H, Ar), 3.59–3.49 (m, 2H, NCH_2_), 2.98–2.89 (m, 2H, CH_2_Ar). ^1^H-NMR (500 MHz, CF_3_COOD) δ: 7.94–7.88 (m, 2H, Ar), 7.80–7.74 (m, 1H, Ar), 7.58 (s, 1H, Ar), 7.55–7.45 (m, 3H, Ar), 7.18–7.10 (m, 2H, Ar), 3.90 (t, *J* = 7.2 Hz, 2H, NCH_2_), 3.14 (t, *J* = 7.2 Hz, 2H, CH_2_Ar). ^1^H-NMR (500 MHz, DMSO-*d*_6_ + 5%(mass)CF_3_SO_3_H) δ: 8.45 (s, 1H, NH), 7.73 (d, *J* = 8.0 Hz, 2H, Ar), 7.66–7.59 (m, 1H, Ar), 7.41 (d, *J* = 8.0 Hz, 2H, Ar), 7.24–7.11 (m, 2H, Ar), 6.96 (d, *J* = 8.1 Hz, 1H, Ar), 6.91–6.84 (m, 1H, Ar), 3.49–3.55 (m, 2H, NCH_2_), 2.91 (t, *J* = 7.2 Hz, 2H, CH_2_Ar). ^13^C-NMR (125 MHz, DMSO-*d*_6_ +5%(mass)CF_3_SO_3_H) δ: 160.76, 154.98, 144.54, 144.19, 143.72, 142.43, 130.17, 129.63, 127.87, 126.19, 119.92, 116.90, 116.30, 104.61, 40.19, 35.21. MS (APCI): *m*/*z* = 387.0 [M+H]^+^; *m*/*z* = 385.0 [M–H]^−^. Anal.: Calcd. for C_18_H_18_N_4_O_4_S (%): C, 55.95; H, 4.70; N, 14.50; S, 8.30. Found: C, 55.79; H, 4.64; N, 14.68; S, 8.54.

*N-{2-[4-(Aminosulfonyl)phenyl]ethyl}-3-(2-hydroxy-4-methylphenyl)-1H-pyrazole-5-carboxamide* (**5b**). Yield 64%; m.p. 254–255 °C. ^1^H-NMR (500 MHz, DMSO-*d*_6_ +5%(mass)CF_3_SO_3_H) δ: 8.39 (s, 1H, NH), 7.72 (d, *J* = 8.0 Hz, 2H, Ar), 7.49 (d, *J* = 7.9 Hz, 1H, Ar), 7.40 (d, *J* = 8.0 Hz, 2H, Ar), 7.14 (s, 1H, Ar), 6.76 (s, 1H, Ar), 6.71–6.68 (m, 1H, Ar), 3.52 (t, *J* = 7.2 Hz, 2H, NCH_2_), 2.91 (t, *J* = 7.2 Hz, 2H, CH_2_Ar), 2.22 (s, 3H, CH_3_). ^13^C-NMR (125 MHz, DMSO-*d*_6_ +5%(mass)CF_3_SO_3_H) δ: 160.62, 154.88, 144.49, 144.14, 143.67, 142.41, 140.10, 129.59, 127.68, 126.15, 120.79, 117.24, 113.39, 104.26, 40.16, 35.16, 21.28. MS (APCI): *m*/*z* = 401.0 [M+H]^+^; *m*/*z* = 399.0 [M–H]^−^. Anal.: Calcd. for C_19_H_20_N_4_O_4_S (%): C, 56.99; H, 5.03; N, 13.99; S, 8.01. Found: C, 57.14; H,5.08; N, 13.84; S, 8.19.

*N-{2-[4-(Aminosulfonyl)phenyl]ethyl}-3-(2-hydroxy-4,5-dimethylphenyl)-1H-pyrazole-5-carboxamide* (**5d**). Yield 61%; m.p. 256–258 °C. ^1^H-NMR (500 MHz, DMSO-*d*_6_ +5%(mass)CF_3_SO_3_H) δ: 8.46 (s, 1H, NH), 7.74 (d, *J* = 8.0 Hz, 2H, Ar), 7.45–7.33 (m, 3H, Ar), 7.17 (s, 1H, Ar), 6.75 (s, 1H, Ar), 3.55–3.49 (m, 2H, NCH_2_), 2.92 (t, *J* = 7.1 Hz, 2H, CH_2_Ar), 2.14–2.11 (m, 6H, 2CH_3_). ^13^C-NMR (125 MHz, DMSO-*d*_6_ +5%(mass)CF_3_SO_3_H) δ: 160.72, 152.84, 144. 52, 144.12, 143.73, 142.40, 138.48, 129.56, 128.33, 127.31, 126.13, 117.87, 113,32, 104.14, 40.12, 35.16, 19.76, 18.80. MS (APCI): *m*/*z* = 415.0 [M+H]^+^; *m*/*z* = 413.1 [M–H]^−^. Anal.: Calcd. for C_20_H_22_N_4_O_4_S (%): C, 57.96; H, 5.35; N, 13.52; S, 7.74, Found: C, 58.04; H, 5.40; N, 13.41; S, 7.92.

*N-{2-[4-(Aminosulfonyl)phenyl]ethyl}-3-(2-hydroxy-3,5-dimethylphenyl)-1H-pyrazole-5-carboxamide* (**5e**). Yield 65%; m.p. 273–275 °C. ^1^H-NMR (500 MHz, DMSO-*d*_6_ +5%(mass)CF_3_SO_3_H) δ: 8.56 (s, 1H, NH), 7.72 (d, *J* = 8.1 Hz, 2H, Ar), 7.41 (d, *J* = 8.1 Hz, 2H, Ar), 7.26 (s, 1H, Ar), 7.20 (s, 1H, Ar), 6.88 (s, 1H, Ar), 3.53–3.47 (m, 2H, NCH_2_), 2.91 (t, *J* = 7.2 Hz, 2H, CH_2_Ar), 2.19 (s, 3H, CH_3_), 2.14 (s, 3H, CH_3_). ^13^C-NMR (125 MHz, DMSO-*d*_6_ +5%(mass)CF_3_SO_3_H) δ: 159.41, 151.33, 149.53, 144.05, 142.45, 140.27, 131.73, 129.59, 128.15, 126.16, 125.59, 124.93, 116.00, 102.78, 40.24, 35.07, 20.49, 16.4. MS (APCI): *m*/*z* = 415.2 [M+H]^+^; *m*/*z* = 413.0 [M–H]^−^. Anal.: Calcd. for C_20_H_22_N_4_O_4_S (%): C, 57.96; H, 5.35; N, 13.52; S, 7.74. Found: C, 57.88; H, 5.40; N, 13.34; S, 7.96.

*N-{2-[4-(Aminosulfonyl)phenyl]ethyl}-3-(5-chloro-2-hydroxyphenyl)-1H-pyrazole-5-carboxamide* (**5f**). Yield 72%; m.p. 260–261 °C. ^1^H-NMR (500 MHz, DMSO-*d*_6_ +5%(mass)CF_3_SO_3_H) δ: 8.42 (s, 1H, NH), 7.75–7.68 (m, 3H, Ar), 7.41 (d, *J* = 8.0 Hz, 2H, Ar), 7.24 (s, 1H, Ar), 7.19 (dd, *J* = 8.7 Hz, *J* = 2.7 Hz, 1H, Ar), 6.96 (d, *J* = 8.7 Hz, 1H, Ar), 3.53–3.48 (m, 2H, NCH_2_), 2.91 (t, *J* = 7.1 Hz, 2H, CH_2_Ar). ^13^C-NMR (125 MHz, DMSO-*d*_6_ +5%(mass)CF_3_SO_3_H) δ: 160.85, 153.79, 144.24, 143.70, 143.34, 142.41, 129.65, 129.35, 126.94, 126.20, 123.50, 118.59, 105.35, 40.18, 35.23. MS (APCI): *m*/*z* = 423.0([M(^37^Cl)+H]^+^; 30); *m*/*z* = 421.0([M(^35^Cl)+H]^+^, 100); *m*/*z* = 421.0 ([M(^37^Cl)–H]^−^, 30); *m*/*z* = 419.0 ([M(^35^Cl)–H]^−^, 100). Anal.: Calcd. for C_18_H_17_ClN_4_O_4_S (%): C, 51.37; H, 4.04; N, 13.31; S, 7.62. Found: C, 51.24; H, 4.08; N, 13.45; S, 7.75.

### 3.2. General Procedure for the Synthesis of 4-Oxo-1-aryl-1,4-dihydropyridazine-3-carboxylic acid **8a**–**d**

A solution of 30 mmol of the corresponding methyl 3-oxo-2-(arylhydrazono)butanoates 6 in 30 ml DMFDMA was refluxed for 2 h. The reaction mixture was cooled to ambient temperature and then chilled to 0 °C. The formed precipitate was filtered off, then washed with ethyl acetate (5 mL) to yield methyl 4-oxo-1-aryl-1,4-dihydropyridazine-3-carboxylates **7a**–**d** as a pale-yellow solids. Then, a solution of 2 g (50 mmol) of sodium hydroxide in 50 ml of water was added at 0 °C to the suspension of 7 in 130 ml of methanol and was stirred at ambient temperature for 3 h. Next, 52 ml of 1M water solution of hydrochloric acid was added to this suspension at 0 °C. The resulting mixture was concentrated under reduced pressure; the formed precipitate was filtered off, then washed with water and dried at 45–50 °C to yield 8a-d as yellow solids.

*1-(3-Methylphenyl)-4-oxo-1,4-dihydropyridazine-3-carboxylic acid* (**8a**). Yield 72%; m.p. 217–219 °C. ^1^H-NMR (500 MHz, DMSO-*d*_6_) δ: 14.87 (s, 1H, COOH), 9.05 (d, *J* = 7.7 Hz, 1H, H-6), 7.62–7.53 (m, 2H, Ar), 7.51–7.44 (m, 1H, Ar), 7.35 (d, *J* = 7.2 Hz, 1H, Ar), 7.03 (d, *J* = 7.7 Hz, 1H, H-5), 2.42 (s, 3H, CH_3_). Anal.: Calcd. for C_12_H_10_N_2_O_3_ (%): C, 62.61; H, 4.38; N, 12.17. Found: C, 62.49; H, 4.35; N, 12.09.

*1-(4-Fluorophenyl)-4-oxo-1,4-dihydropyridazine-3-carboxylic acid* (**8b**). Yield 65%; m.p. 227–229 °C. ^1^H-NMR (500 MHz, DMSO-*d*_6_) δ: 14.50 (s, 1H, COOH), 9.03 (d, *J* = 7.8 Hz, 1H, H-6), 7.91–7.79 (m, 2H, Ar), 7.47–7.35 (m, 2H, Ar), 7.02 (d, *J* = 7.8 Hz, 1H, H-5). Anal. Calcd. for C_11_H_7_FN_2_O_3_ (%): C, 56.42; H, 3.01; N, 11.96. Found: C, 56.33; H,3.09; N, 11.80.

*1-(3-Chlorophenyl)-4-oxo-1,4-dihydropyridazine-3-carboxylic acid* (**8c**). Yield 67%; m.p. 224–226 °C. ^1^H-NMR (500 MHz, DMSO-*d*_6_) δ: 14.62 (s, 1H, COOH), 9.06 (d, *J* = 7.8 Hz, 1H, H-6), 7.92–7.87 (m, 1H, Ar), 7.78–7.73 (m, 1H, Ar), 7.62–7.51 (m, 2H, Ar), 7.03 (d, *J* = 7.8 Hz, 1H, H-5). Anal.: Calcd. for C_11_H_7_ClN_2_O_3_ (%): C, 52.71; H, 2.82; N, 11.18. Found: C, 52.79; H, 2.88; N, 11.27.

*1-(4-Chlorophenyl)-4-oxo-1,4-dihydropyridazine-3-carboxylic acid* (**8d**). Yield 75%; m.p. 225–227 °C. ^1^H-NMR (500 MHz, DMSO-*d*_6_) δ: 14.84 (s, 1H, COOH), 9.09 (d, *J* = 7.8 Hz, 1H, H-6), 7.83 (d, *J* = 8.9 Hz, 2H, Ar), 7.63 (d, *J* = 8.9 Hz, 2H, Ar), 702 (d, *J* = 7.8 Hz, 1H, H-5). Anal.: Calcd. for C_11_H_7_ClN_2_O_3_ (%): C, 52.71; H, 2.82; N, 11.18. Found: C, 52.83; H, 2.77; N, 11.31.

### 3.3. General Procedure for the Synthesis of 4-Oxo-1-aryl-1,4-dihydropyridazine-3-carbonyl chloride **9a**–**d**

To the suspension of 2 mmol of 8 in 20 mL of dry chloroform 2 mL of freshly distilled thionyl chloride and one drop of dry DMF was added. The mixture was heated for 1 h at 45–50 °C and then evaporated to dryness under reduced pressure. The residue was treated with 5 mL of dry toluene and the mixture was evaporated to dryness under reduced pressure again. The residues were used for the next step without any additional purification.

### 3.4. General Procedure for the Synthesis of N-[4-(aminosulfonyl)phenyl]-1-aryl-4-oxo-1,4-dihydropyridazine-3-carboxamide **10a**–**d**

To a solution of 0.17 g (1 mmol) of 4-aminobenzenesulfonamide and 0.11 g (1.1 mmol) of triethylamine in 5 mL of acetonitrile, a solution of 1 mmol of compounds **9a**–**d** in 10 mL acetonitrile was added while stirring and cooling to 0–5 °C. The mixture was stirred for 1 h at ambient temperature and evaporated to dryness under reduced pressure. Then, 15 mL of water was added, and the formed precipitate was filtered off, dried in air and crystallized from the DMF:ethanol mixture to yield **10a**–**d** as brown or yellowish-brown solids.

*N-[4-(Aminosulfonyl)phenyl]-1-(3-methylphenyl)-4-oxo-1,4-dihydropyridazine-3-carboxamide* (**10a**). Yield 90%; m.p. 295–297 °C. ^1^H-NMR (500 MHz, DMSO-*d*_6_) δ: 12.10 (s, 1H, NH), 8.98 (d, *J* = 7.8 Hz, 1H, H-6), 7.91–7.81 (m, 4H, Ar), 7.65–7.54 (m, 2H, Ar), 7.52–7.45 (m, 1H, Ar), 7.35–7.26 (m, 3H, NH_2_, Ar), 6.92 (d, *J* = 7.8 Hz, 1H, H-5), 2.42 (s, 3H, CH_3_). MS (APCI): *m*/*z* = 385.0 [M+H]^+^; *m*/*z* = 383.0 [M–H]^−^. Anal.: Calcd. for C_18_H_16_N_4_O_4_S (%): C, 56.24; H, 4.20; N, 14.57; S, 8.34. Found: C, 56.19; H, 4.28; N, 14.39; S, 8.51.

*N-[4-(Aminosulfonyl)phenyl]-1-(4-fluorophenyl)-4-oxo-1,4-dihydropyridazine-3-carboxamide* (**10b**). Yield 90%; m.p. 313–315 °C. ^1^H-NMR (500 MHz, DMSO-*d*_6_) δ: 12.11 (s, 1H, NH), 8.97 (d, *J* = 7.8 Hz, 1H, H-6), 7.92–7.78 (m, 6H, Ar), 7.51–7.42 (m, 2H, Ar), 7.33 (s, 2H, NH_2_), 6.93 (d, *J* = 7.8 Hz, 1H, H-5). ^13^C-NMR (126 MHz, DMSO-*d*_6_) δ: 169.29, 161.64 (d, J_CF_ = 246.4 Hz), 159.97, 147.22, 142.10, 140.96, 139.46, 139.33, 126.97, 123.92 (d, *J_CF_* = 9.0 Hz), 120.61, 119.52, 116.53 (d, *J_CF_* = 23.4 Hz). MS (APCI): *m*/*z* = 389.0 [M+H]^+^; *m*/*z* = 387.0 [M–H]^−^. Anal.: Calcd. for C_17_H_13_FN_4_O_4_S (%): C, 52.57; H, 3.37; N, 14.43; S, 8.26. Found: 52.69; H, 3,31; N, 14.28; S, 8.47.

*N-[4-(Aminosulfonyl)phenyl]-1-(3-chlorophenyl)-4-oxo-1,4-dihydropyridazine-3-carboxamide* (**10c**). Yield 82%; m.p. 297–299 °C. ^1^H-NMR (500 MHz, DMSO-*d*_6_) δ: 11.98 (s, 1H, NH), 9.05 (d, *J* = 7.9 Hz, 1H, H-6), 7.95 (d, *J* = 2.1 Hz, 1H, Ar), 7.91–7.78 (m, 5H, Ar), 7.69–7.56 (m, 2H, Ar), 7.32 (s, 2H, NH_2_), 6.93 (d, *J* = 7.9 Hz, 1H, H-5). ^13^C-NMR (126 MHz, DMSO-*d*_6_) δ: 169.82, 160.34, 147.97, 144.21, 142.16, 141.36, 139.80, 134.43, 131.81, 128.86, 127.39, 121.71, 120.90, 120.37, 119.96. MS (APCI): *m*/*z* = 406.9([M(^37^Cl)+H]^+^; 30); *m*/*z* = 404.9([M(^35^Cl)+H]^+^, 100); *m*/*z* = 405.0 ([M(^37^Cl)–H]^−^, 30); *m*/*z* = 402.9 ([M(^35^Cl)–H]^−^, 100). Anal.: Calcd. for C_17_H_13_ClN_4_O_4_S (%): C, 50.44; H, 3.24; N, 13.84; S, 7.92. Found: C, 50.29; H, 3.31; N, 13.65; S, 8.09.

*N-[4-(Aminosulfonyl)phenyl]-1-(4-chlorophenyl)-4-oxo-1,4-dihydropyridazine-3-carboxamide* (**10d**). Yield 90%; m.p. 310–312 °C (lit. 190 °C [7]). ^1^H-NMR (500 MHz, DMSO-*d*_6_) δ: 12.03 (s, 1H, NH), 9.01 (d, *J* = 7.8 Hz, 1H, H-6), 7.93–7.81 (m, 6H, Ar), 7.68 (d, *J* = 8.7 Hz, 2H, Ar), 7.32 (s, 2H, NH_2_), 6.93 (d, *J* = 7.8 Hz, 1H, H-5). ^13^C-NMR (126 MHz, DMSO-*d*_6_) δ: 169.27, 159.93, 147.51, 141.66, 141.59, 140.92, 139.33, 133.04, 129.63, 126.93, 123.06, 120.52, 119.48. MS (APCI): *m*/*z* = 407.0([M(^37^Cl)+H]^+^; 30); *m*/*z* = 405.0([M(^35^Cl)+H]^+^, 100); *m*/*z* = 405.0 ([M(^37^Cl)–H]^−^, 30); *m*/*z* = 403.0 ([M(^35^Cl)–H]^−^, 100). Anal.: Calcd. for C_17_H_13_ClN_4_O_4_S (%): C, 50.44; H, 3.24; N, 13.84; S, 7.92. Found: C, 50.47; H, 3.29; N, 13.68; S, 8.13. 

### 3.5. Synthesis of N-[4-(aminosulfonyl)phenyl]-6,7-dimethoxy-1-methyl-1,4-dihydroindeno[1,2-c]pyrazole-3-carboxamide **15**

*Sodium 1-(5,6-dimethoxy-1-oxo-1,3-dihydro-2H-inden-2-ylidene)-2-methoxy-2-oxoethanolate* (**12**). A solution of 5.76 g (30 mmol) of 5,6-dimethoxyindan-1-one 11 and 4.72 g (40 mmol) of dimethyl oxalate in 50 mL methanol was added in one portion to a solution of 2.7 g (50 mmol) of sodium methylate in 30 ml of methanol. The reaction mixture was heated for 2 h at 55–60 °C, cooled to ambient temperature and then chilled to 0 °C. The formed precipitate was filtered off, washed with methanol (10 mL) and then diethyl ether (5 mL) to yield 12 as a yellow solid. It was used for the next step without additional purification.

*Methyl 6,7-dimethoxy-1-methyl-1,4-dihydroindeno[1,2-c]pyrazole-3-carboxylate* (**13**). To a stirred solution of 2.48 g (30 mmol) of methylhydrazine hydrochloride in 50 ml of methanol and 10 ml of glacial acetic acid, 7.5 g (25 mmol) of 12 was added. The mixture was heated for 12 h at 60 °C, evaporated to dryness under reduced pressure, and the resulting residue was treated with 50 mL of water. The formed precipitate was filtered off, washed with water (10 mL), then methanol (5 mL) and finally twice crystallized from methanol to afford 13 as a colorless solid. Yield 55%; m.p. 187–188 °C. ^1^H-NMR (500 MHz, DMSO-*d*_6_) δ: 7.32 (s, 1H, Ar), 7.23 (s, 1H, Ar), 4.13 (s, 3H, CH_3_), 3.85 (s, 3H, CH_3_), 3.81 (s, 3H, CH_3_), 3.80 (s, 3H, CH_3_), 3.55 (s, 2H, CH_2_). ^13^C-NMR (125 MHz, DMSO-*d*_6_) δ: 162.59, 150.30, 148.66, 148.63, 141.40, 135.77, 128.59, 124.01, 110.96, 103.98, 56.52, 56.18, 51.90, 38.67, 29.47. Anal.: Calcd. for C_15_H_16_N_2_O_4_ (%): C, 62.49; H, 5.59; N, 9.72. Found (%): C, 62.55; H, 5.51; N, 9.83.

*6,7-Dimethoxy-1-methyl-1,4-dihydroindeno[1,2-c]pyrazole-3-carboxylic acid* (**14**). A stirred solution of 2.88 g (10 mmol) of compound 13 in 30 mL of methanol was treated with a solution of 1.68 g (30 mmol) of potassium hydroxide in 15 mL water. The resulting mixture was stirred at 50 °C for 18 h (TLC control), cooled to ambient temperature and acidified with formic acid. The formed precipitate was filtered off, washed with water, dried in air and crystallized from ethanol to yield 14 as colorless solid. Yield 91%; m.p. 235–236 °C. ^1^H-NMR (500 MHz, DMSO-*d*_6_,) δ: 12.60 (s, 1H, COOH), 7.28 (s, 1H, Ar), 7.19 (s, 1H, Ar), 4.10 (s, 3H, CH_3_), 3.83 (s, 3H, CH_3_), 3.78 (s, 3H, CH_3_), 3.50 (s, 2H, CH_2_). ^13^C-NMR (125 MHz, DMSO-*d*_6_) δ: 163.64, 150.15, 148.59, 148.49, 141.47, 136.85, 128.64, 124.16, 110.92, 103.86, 56.48, 56.14, 38.54, 29.48. MS (APCI): *m*/*z* = 275.1 [M+H]^+^; *m*/*z* = 273.1 [M–H]^−^. Calcd. for C_14_H_14_N_2_O_4_ (%): C, 61.31; H, 5.14; N, 10.21 Found (%): C, 61.43; H, 5.19; N, 10.08.

*N-[4-(Aminosulfonyl)phenyl]-6,7-dimethoxy-1-methyl-1,4-dihydroindeno[1,2-c]pyrazole-3-carboxamide* (**15**). To a stirred solution of 0.28 g (1 mmol) of 14 in 10 mL of anhydrous toluene 1 mL of thionyl chloride was added. The mixture was heated for 1 h at 80 °C and then evaporated to dryness under reduced pressure. The residue was treated with 5 mL of toluene and the resulted mixture was continuously evaporated to dryness under reduced pressure again. The residue was dissolved in 10 mL of acetonitrile and then was added to a solution of 0.17 g (1 mmol) of 4-aminobenzenesulfonamide and 0.11 g (1.1 mmol) of triethylamine in 5 mL acetonitrile while stirring and cooling (0–5 °C). The mixture was stirred for 1 h, evaporated to dryness under reduced pressure, residue was treated with 15 mL of water, the formed precipitate was filtered off, dried at air and crystallized from the DMF:ethanol mixture (1:7 *v*/*v*) to yield 15 as colorless solid. Yield 90%; m.p. 286–287 °C. ^1^H-NMR (500 MHz, DMSO-*d*_6_) δ: 10.36 (s, 1H, NH), 8.02 (d, *J* = 8.5 Hz, 2H, Ar), 7.76 (d, *J* = 8.5 Hz, 2H, Ar), 7.34 (s, 1H, Ar), 7.28–7.21 (m, 3H, Ar, NH_2_), 4.18 (s, 3H, CH_3_), 3.85 (s, 3H, CH_3_), 3.79 (s, 3H, CH_3_), 3.62 (s, 2H, CH_2_). ^13^C-NMR (125 MHz, DMSO-*d*_6_) δ: 160.96, 151.00, 148.70, 148.63, 142.33, 141.88, 139.33, 138.82, 127.55, 126.85, 123.96, 120.05, 110.97, 104.06, 56.56, 56.16, 38.50, 29.43. MS (APCI): *m*/*z* = 429.0 [M+H]^+^; *m*/*z* = 427.0 [M–H]^−^. Anal.: Calcd. for C_20_H_20_N_4_O_5_S (%): C, 56.07; H, 4.70; N, 13.08; S, 7.48. Found: C, 55.92; H, 4,78; N, 12.95; S, 7.61.

### 3.6. Molecular Docking Studies

Molecular modeling studies were performed using the software AutoDock 4.2 [47]. Protein Data Bank was also used in order to obtain the he crystal structures of *h*CA I (PDB code 3W6H) and *h*CA II (PDB code 3HS4) cytosolic isoforms as well as *h*CA IX (PDB code 3IAI) and *h*CA XII (PDB code 1JD0) transmembrane tumor-associated isoforms [48]. All of the procedure was carried out as described in our previous work [49]. 

### 3.7. CA Inhibition Assay

An Applied Photophysics stopped-flow instrument was used for assaying the CA-catalyzed CO_2_ hydration activity [50]. Phenol red (at a concentration of 0.2 mM) was used as an indicator, working at the absorbance maximum of 557 nm, with 20 mM Hepes (pH 7.4) as a buffer, and 20 mM Na_2_SO_4_ (for maintaining constant ionic strength), following the initial rates of the CA-catalyzed CO_2_ hydration reaction for a period of 10–100 s. The CO_2_ concentrations ranged from 1.7 to 17 mM for the determination of the kinetic parameters and inhibition constants [48]. The non-catalyzed CO_2_ hydration was not subtracted from these curves and accounted for the remaining observed activity even at high concentration of inhibitor, being in the range of 16–25%. However, the background activity from the uncatalyzed reaction was always subtracted when IC50 values were obtained by using the data analysis software for the stopped-flow instrument. Enzyme concentrations ranged between 5 nM and 12 nM. For each inhibitor, at least six traces of the initial 5–10% of the reaction were used for determining the initial velocity. The uncatalyzed rates were determined in the same manner and subtracted from the total observed rates. Stock solutions of the inhibitor (0.1 mM) were prepared in distilled–deionized water, and dilutions up to 0.01 nM were performed thereafter with the assay buffer. Inhibitor and enzyme solutions were preincubated together for 15 min at room temperature prior to the assay, to allow for the formation of the E–I complex. The inhibition constants were obtained by nonlinear least-squares methods using PRISM 3 and the Cheng–Prusoff equation as reported earlier and represented the mean from at least three different determinations. All CA isoforms were recombinant proteins obtained in-house, as reported earlier [2,51,52].

## 4. Conclusions

In conclusion, we synthesized and investigated a novel series of sulfonamides incorporating pyrazole- and pyridazinecarboxamide moieties for their effective inhibition against different and most relevant human carbonic anhydrase isoforms such as the ubiquitous hCA I, hCA II, and tumor associated isoforms hCA IX and XII, which are involved in a variety of diseases such as glaucoma, retinitis pigmentosa, epilepsy, and tumors. Compound **4c** showed a good selectivity index of 26 on hCA IX compared to hCA I and two times compared to hCA II. On the other hand, compound **10f** was shown to be the most active on hCA II with an SI of 236 compared to hCA I and 8.7 compared to hCA IX. These interesting features make them good candidates for preclinical evaluation in glaucoma or various tumors in which the two enzymes (hCA II and hCA IX) are involved. Furthermore, computational procedures were used to investigate the binding mode of this class of compounds.

## Figures and Tables

**Table 1:** Inhibition data of human CA isoforms I, II, IX and XII with compounds **4a**–**f**, **5a**, **b**, **d**–**f**, **10a**–**d**, **15** and **AAZ** by a stopped flow CO_2_ hydrase assay.

					K_I_ (nM) ^1^			
Com/ds	R^1^	R^2^	R^3^	n	hCAI	hCAII	hCAIX	hCAXII
**4a**	H	H	H	0	3822	866.7	450.2	271.1
**4b**	H	H	Me	0	93.3	308.6	78.9	372.5
**4c**	Me	H	H	0	221.1	16.1	8.5	250.0
**4f**	Cl	H	H	0	154.7	37.2	34.2	90.8
**5a**	H	H	H	2	88.1	31.5	93.8	188.5
**5b**	H	H	Me	2	344.6	16.2	16.1	329.5
**5d**	Me	Me	H	2	218.5	25.2	63.5	61.3
**5e**	Me	H	Me	2	71.4	16.2	52.3	191.2
**5f**	Cl	H	H		2150	9.1	79.4	77.3
**10a**	Me	H	-	-	197.9	7.4	41.6	90.8
**10b**	H	F	-	-	481.0	72.5	130.8	94.8
**10c**	Cl	H	-	-	839.7	76.3	568.8	432.8
**10d**	H	Cl	-	-	6.2	8.0	165.2	65.7
**15**	OMe	OMe	-	-	725.6	3.3	6.1	80.5
**AAZ**					250.0	12.1	25.8	5.7
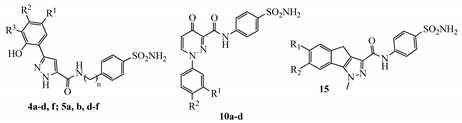								

^1^ Mean from 3 different assays, by a stopped flow technique (errors were in the range of 5–10% of the reported values).

**Table 2:** Molecular docking free binding energies (kcal/mol) and interactions of tested compounds on hCA I, II, IX and XII isoforms.

No	R^1^	R^2^	R^3^	n	hCA Isoform	Estimated Free Binding Energy (Kcal/mol)	Chelating the Zn (II) Ion	Residues Involved in H-Bond Interactions	Residues Involved in Hydrophobic Interactions
**5d**					hCA I	−6.82	Yes	Thr199	Ile191, Val121, Leu198
	Me	Me	H	2	hCA II	−8.17	Yes	Thr199	Val121, Leu198, Thr200
					hCA IX	−6.84	Yes	Thr200	Val121, Trp209
					hCA XII	−8.10	Yes	Gln92, Ser135, Thr200	Trp5, Val121, Leu198
					hCA I	−7.16	Yes	Thr199	Ala132, Ala135, Leu198
**5e**	Me	H	Me	2	hCA II	−8.82	Yes	His94, Thr199	Val121, Phe131, Thr200
					hCA IX	−7.71	Yes	Thr199	Val121, Leu198, Thr200
					hCA XII	−6.21	Yes	-	Val121, Leu198
					hCA I	−12.38	Yes	Thr199	Leu198, His200, Tyr204
**10d**	H	Cl	-	-	hCA II	−9.11	Yes	Gln92, Thr199	Val121, Phe131, Leu198
					hCA IX	−6.55	Yes	Thr199	Val121, Leu198
					hCA XII	−7.02	Yes	His94	Val121, Ala131, Leu198
					hCA I	−4.21	Yes	-	-
**15**	OMe	OMe	-	-	hCA II	−9.25	Yes	Gln92, Thr200	Val121, Phe131, Leu198
					hCA IX	−10.20	Yes	Thr199, Thr200	Val121, Val131, Leu198, Thr200
					hCA XII	−7.11	Yes	Thr200	Val121, Leu198
					hCA I	−8.28	Yes	Gln92	Leu198, Thr199, His200, Pro201, Trp209
**AAZ**					hCA II	−8.87	Yes	Thr199, Thr200	Val121, Phe131, Leu198, Trp209
					hCA IX	−9.02	Yes	Thr199, Thr200	Val121, Val143, Val131, Leu198, Trp209
					hCA XII	−9.14	Yes	Thr199, Thr200	Val121, Val143, Leu198, Trp209

## Data Availability

Not applicable.

## Supplementary Materials

The following are available online: Copies of ^1^H, ^13^C NMR and LCMS Spectra of Products (Figures S1A–S19C).

## Sample Availability

Samples of the compounds are available from the authors.
