# Supplementary material for: Carbonic Anhydrase Inhibition with Sulfonamides Incorporating Pyrazole- and Pyridazinecarboxamide Moieties Provides Examples of Isoform-Selective Inhibitors
Source: Molecules. 2021 Nov 20;26(22):7023. doi: 10.3390/molecules26227023 (PMC8625619; doi:10.3390/molecules26227023)
Supplement: Supplementary file 1 [file molecules-26-07023-s001.zip › molecules-1461225-supplementary.pdf]

Supplementary materials

Carbonic Anhydrase Inhibition with Sulfonamides Incorporating Pyrazole- and Pyridazinecarboxamide Moieties Provides Examples of Isoform-Selective Inhibitors

Andrea Angeli [1,2], Victor Kartsev [3], Anthi Petrou [4], Mariana Pinteala [2], Volodymyr Brovarets [5], Roman Vydzhak [5], Svitlana Panchishin [5], Athina Geronikaki [4,*] and Claudiu T. Supuran [1,*]

[1] NeuroFarba Department, Sezione di Scienze Farmaceutiche, Università degli Studi di Firenze, Via Ugo Schiff 6, Sesto Fiorentino (Florence) 50019, Italy; andrea.angeli@unifi.it
[2] Centre of Advanced Research in Bionanoconjugates and Biopolymers, Petru Poni Institute of Macromolecular Chemistry, Aleea Grigore Ghica-Voda, no. 41A, Iasi 700487, Romania; pinteala@icmpp.ro
[3] InterBioScreen, Chernogolovka, Moscow Region 142432, Russia; vkartsev@ibscreen.chg.ru
[4] Department of Pharmacy, School of Health, Aristotle University of Thessaloniki, Thessaloniki 54124, Greece; anthi.petrou.thessaloniki1@gmail.com
[5] Department of Chemistry of Bioactive Nitrogen-Containing Heterocyclic Bases, V.P. Kukhar Institute of Bioorganic Chemistry and Petrochemistry, NAS of Ukraine 1, Murmanska St, Kyiv 02094, Ukraine; brovarets@bpci.kiev.ua (V.B.); s.slivchyk@mail.enamine.net (S.S.); stepanpilyo@ukr.net (S.P.)
* Correspondence: geronik@oharm.auth.gr (A.G.); claudiu.supuran@unifi.it (C.T.S.)

*

Table of Contents

I. ^{1}H, ^{13}C NMR and LCMS Spectra of Products 2

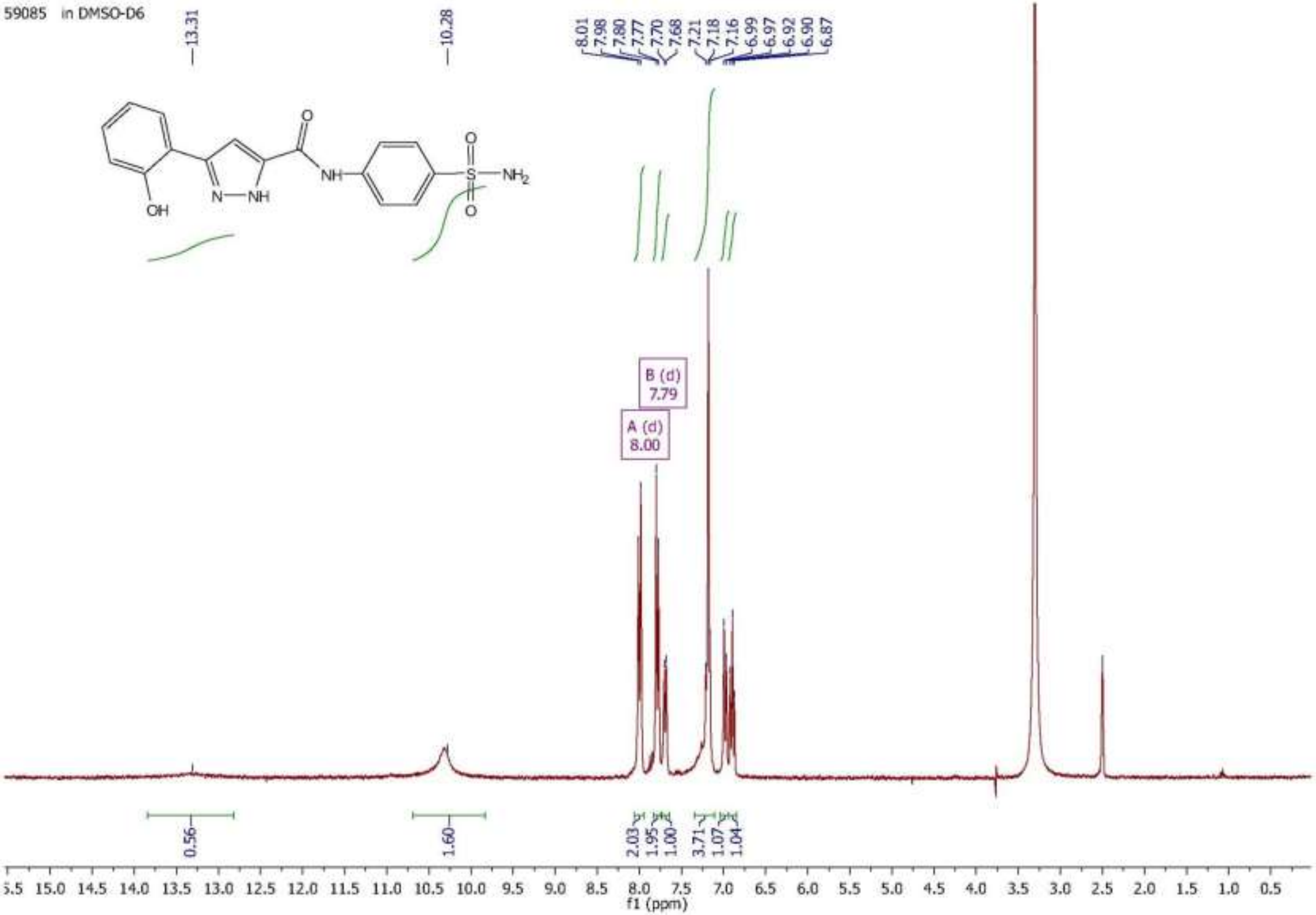

Figure S1A. ^{1}H NMR spectrum of *N-[4-(Aminosulfonyl)phenyl]-3-(2-hydroxyphenyl)-1H-pyrazole-5-carboxamide* (**4a**) in *DMSO-D6*.

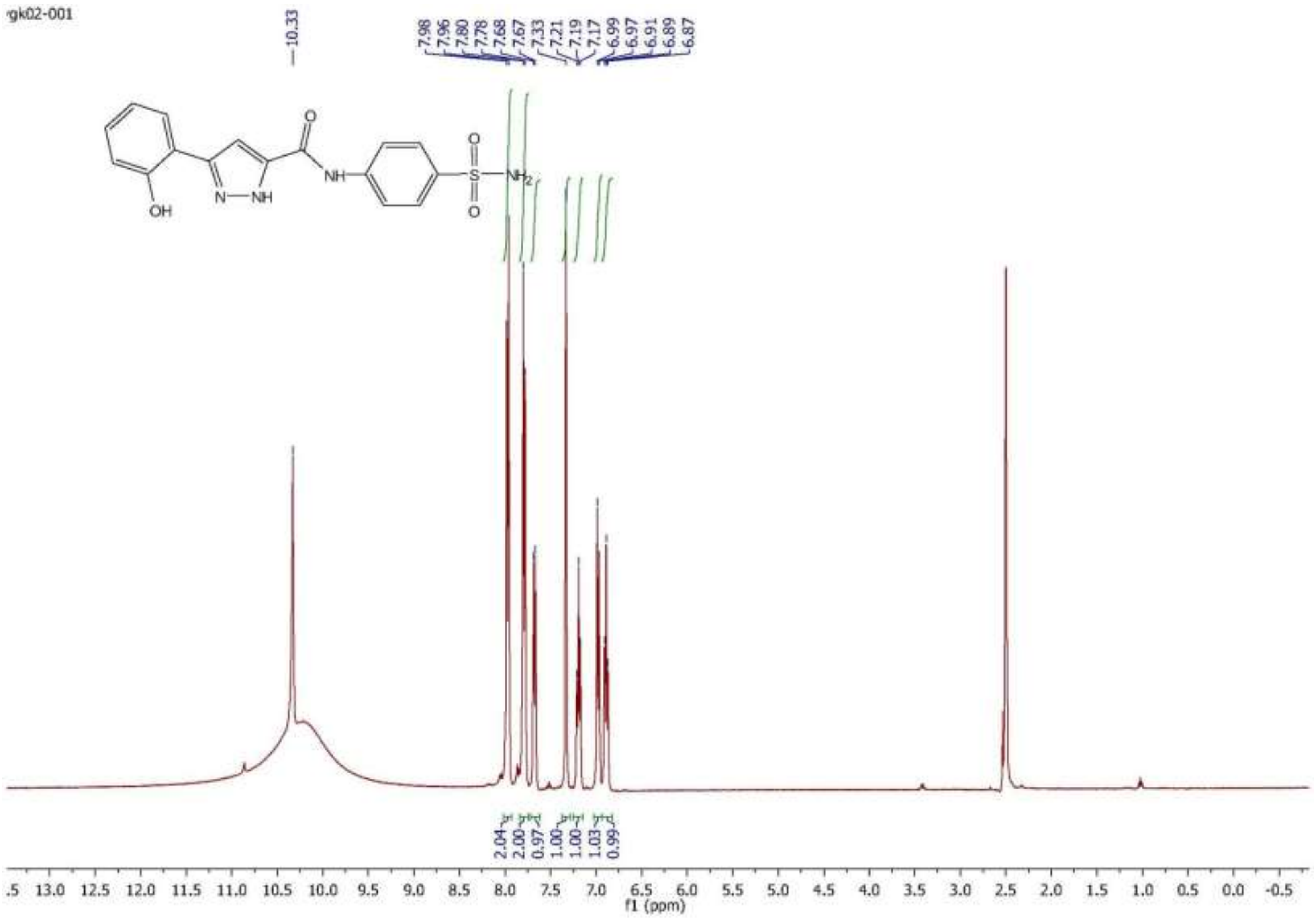

Figure S1B. ^{1}H NMR spectrum of *N-[4-(Aminosulfonyl)phenyl]-3-(2-hydroxyphenyl)-1H-pyrazole-5-carboxamide* (**4a**) in *DMSO-D6*.+ 5%CF_3SO_3H.

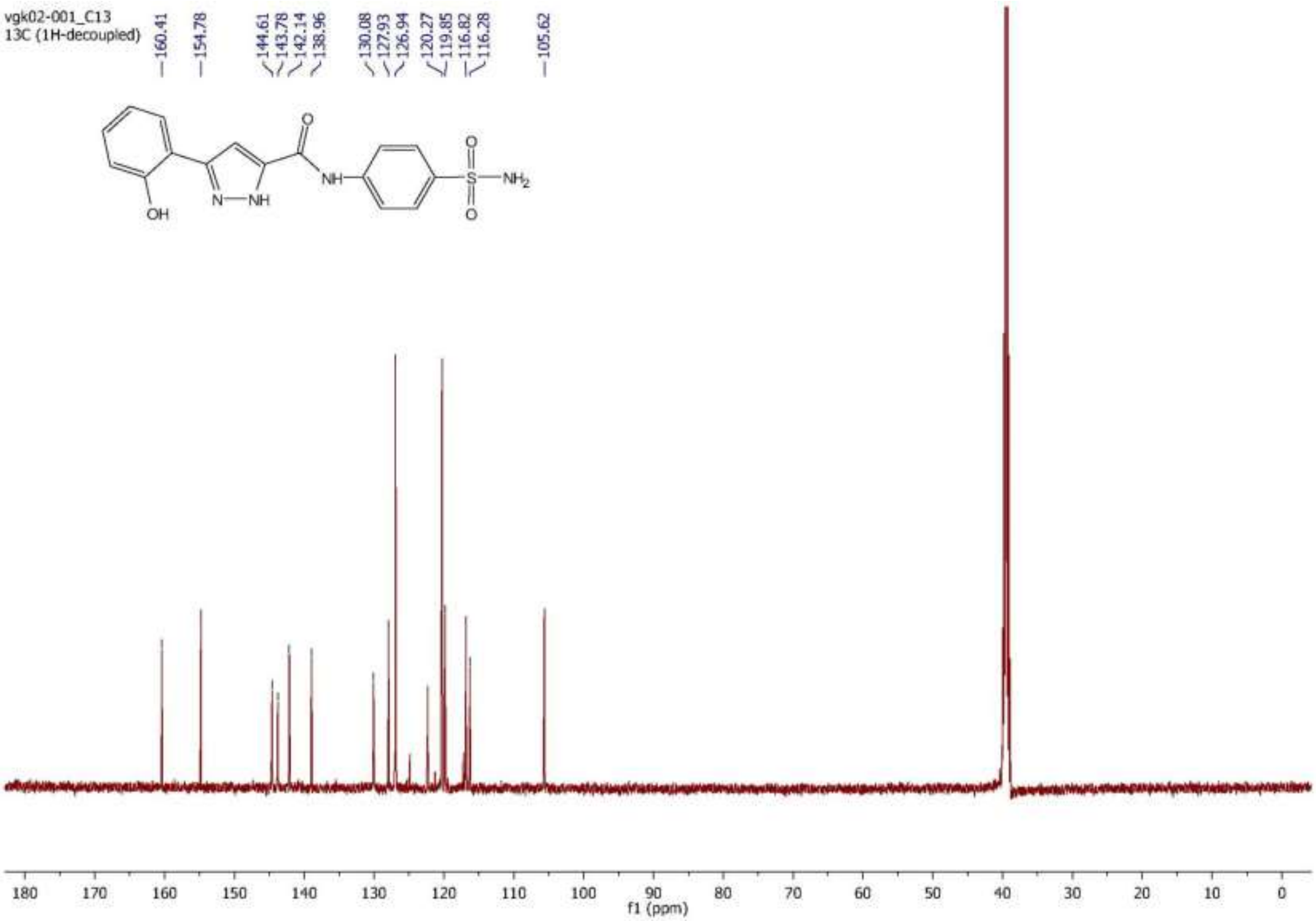

Figure S1C. ^{13}C NMR spectrum of *N-[4-(Aminosulfonyl)phenyl]-3-(2-hydroxyphenyl)-1H-pyrazole-5-carboxamide* (**4a**) in DMSO-*d6*.+ 5%CF_3SO_3H.

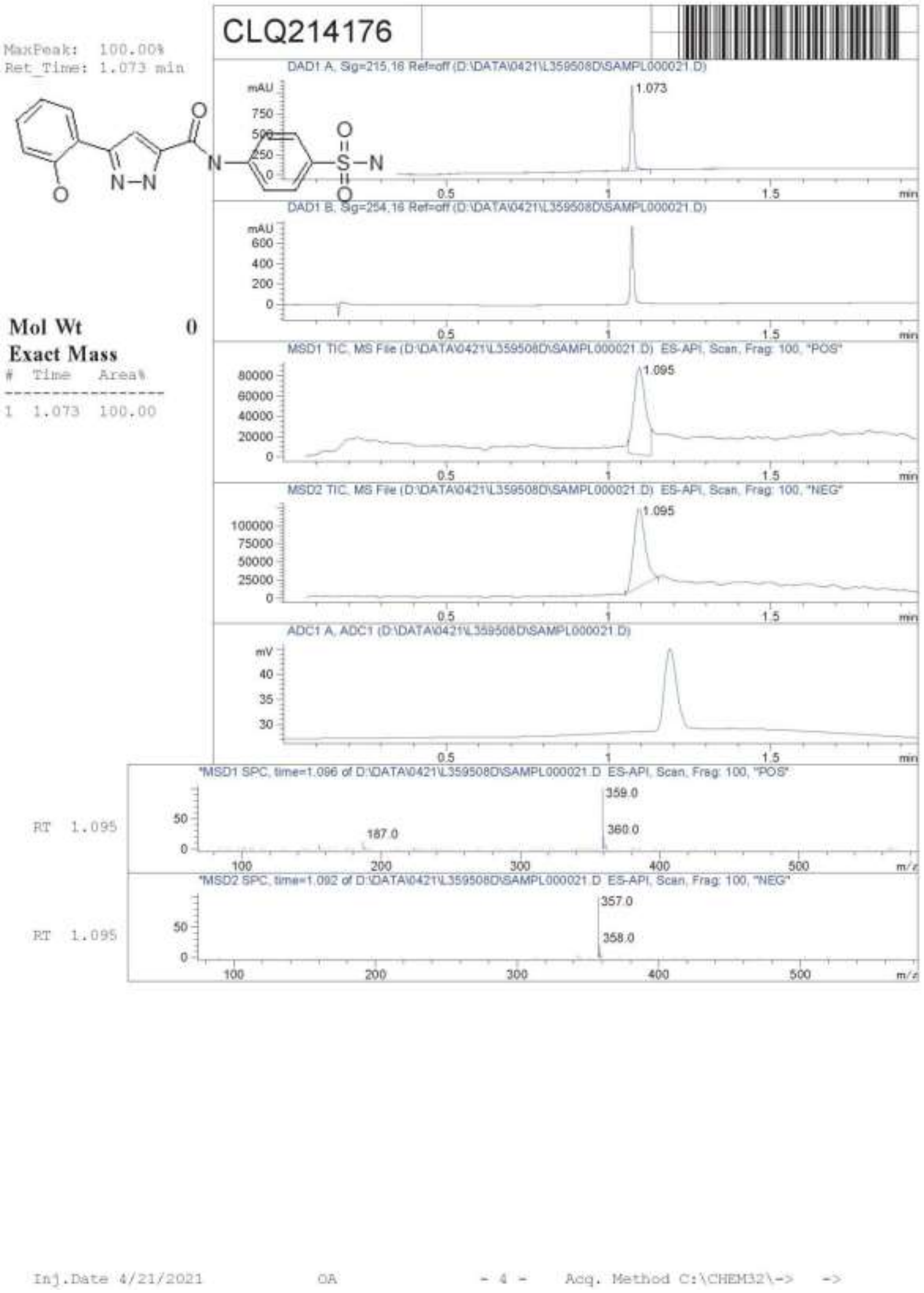

Figure S1D. LCMS spectrum of *N-[4-(Aminosulfonyl)phenyl]-3-(2-hydroxyphenyl)-1H-pyrazole-5-carboxamide* (**4a**).

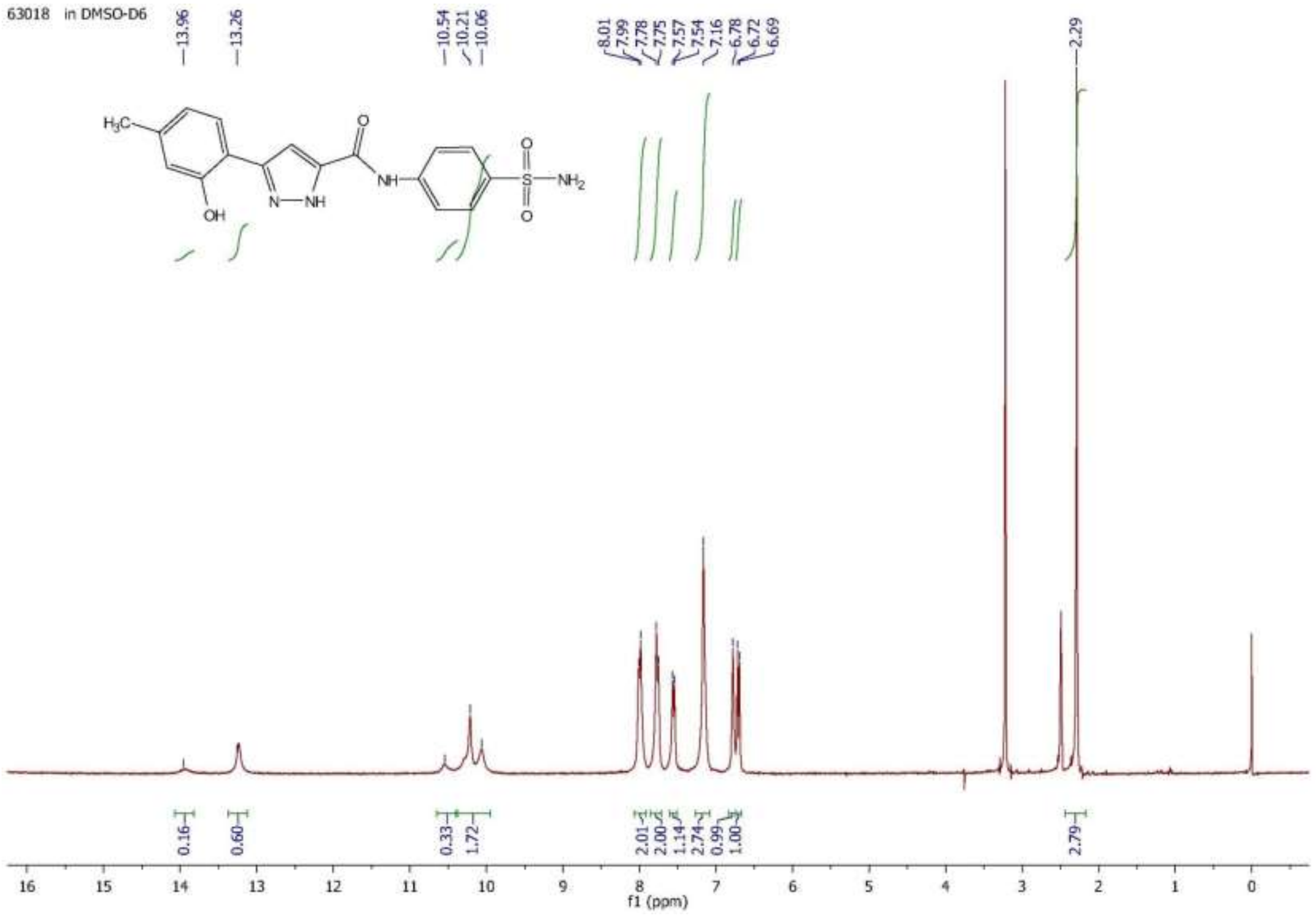

Figure S2A. ^{1}H NMR spectrum of *N-[4-(Aminosulfonyl)phenyl]-3-(2-hydroxy-4-methylphenyl)-1H-pyrazole-5-carboxamide* (**4b**)) in DMSO-*d6*.

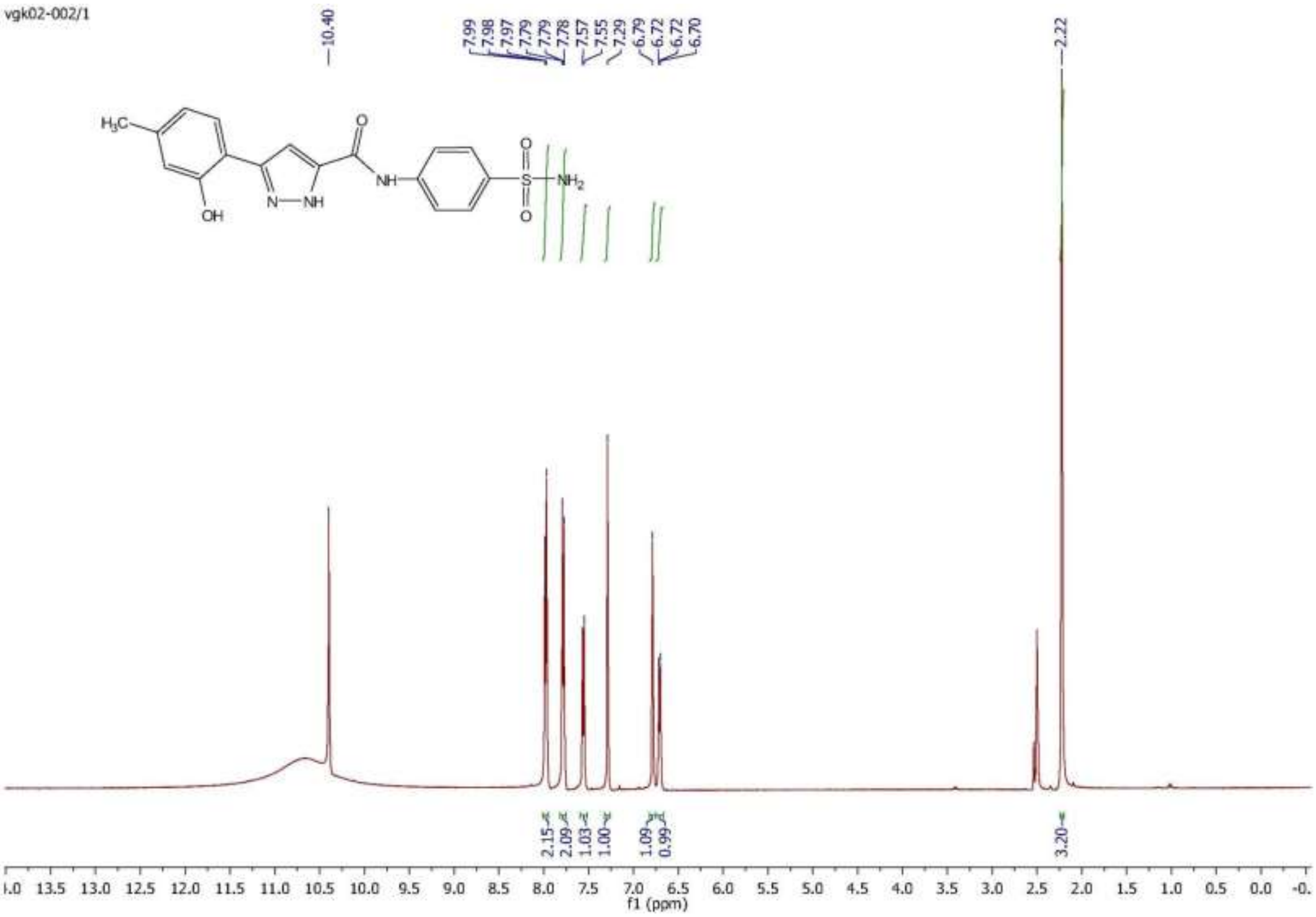

Figure S2B. ^{1}H NMR spectrum of *N-[4-(Aminosulfonyl)phenyl]-3-(2-hydroxy-4-methylphenyl)-1H-pyrazole-5-carboxamide* (**4b**) in DMSO-*d6*.+ 5%CF_3SO_3H.

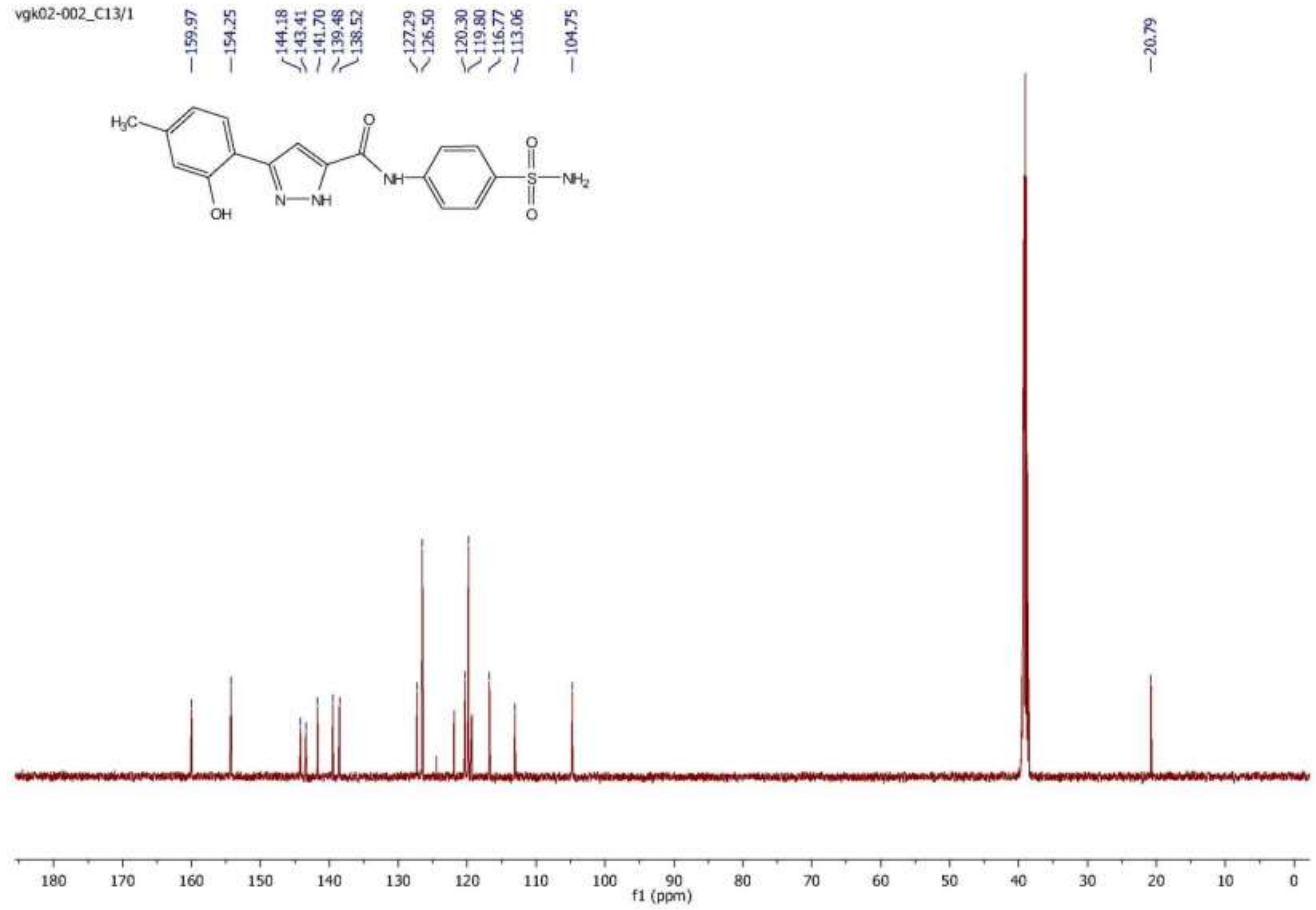

Figure S2C. ^{13}C NMR spectrum of *N-[4-(Aminosulfonyl)phenyl]-3-(2-hydroxy-4-methylphenyl)-1H-pyrazole-5-carboxamide* (**4b**) in DMSO-*d6*.+ 5%CF_3SO_3H.

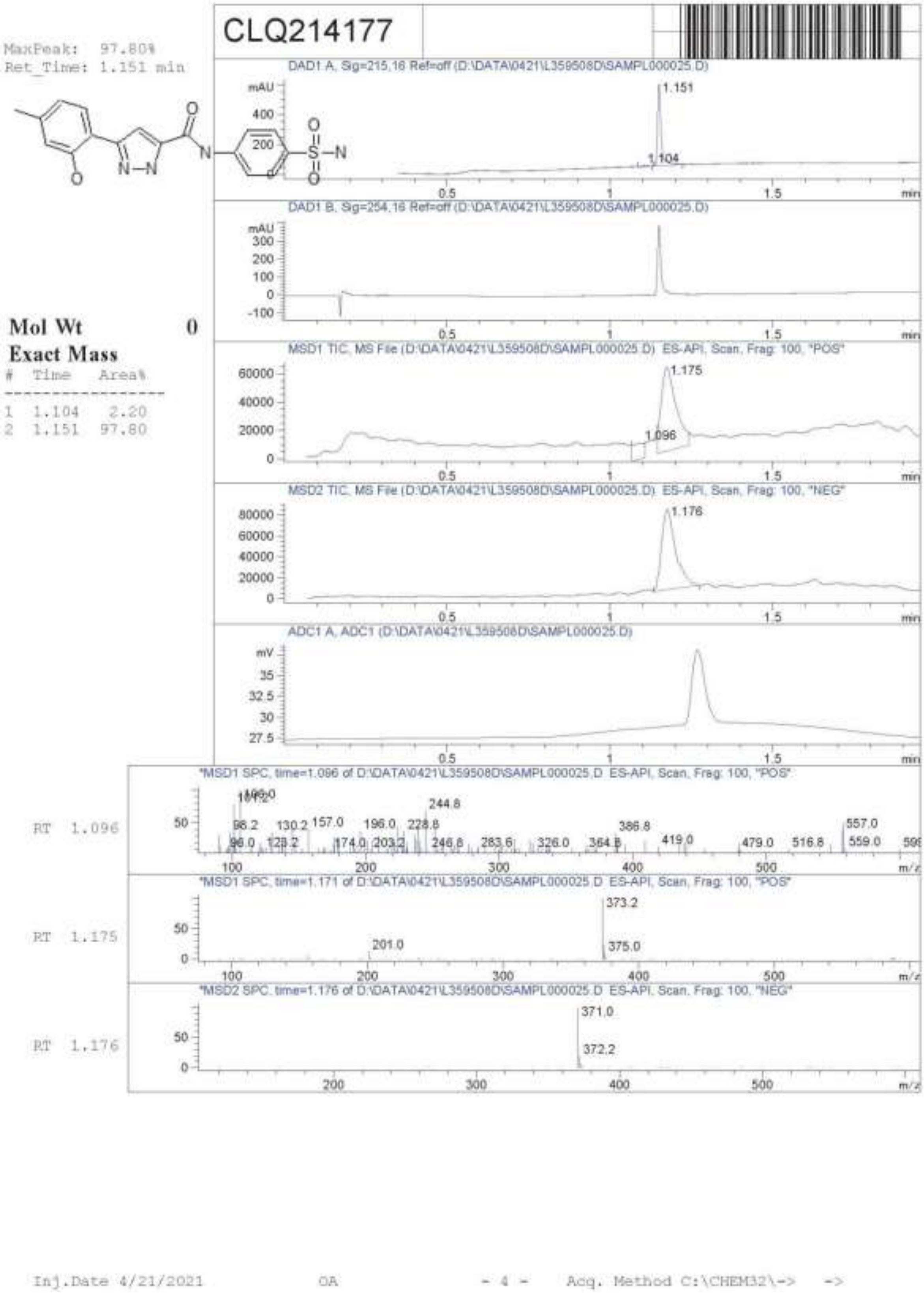

Figure S2D. LCMS spectrum of *N-[4-(Aminosulfonyl)phenyl]-3-(2-hydroxy-4-methylphenyl)-1H-pyrazole-5-carboxamide* (**4b**).

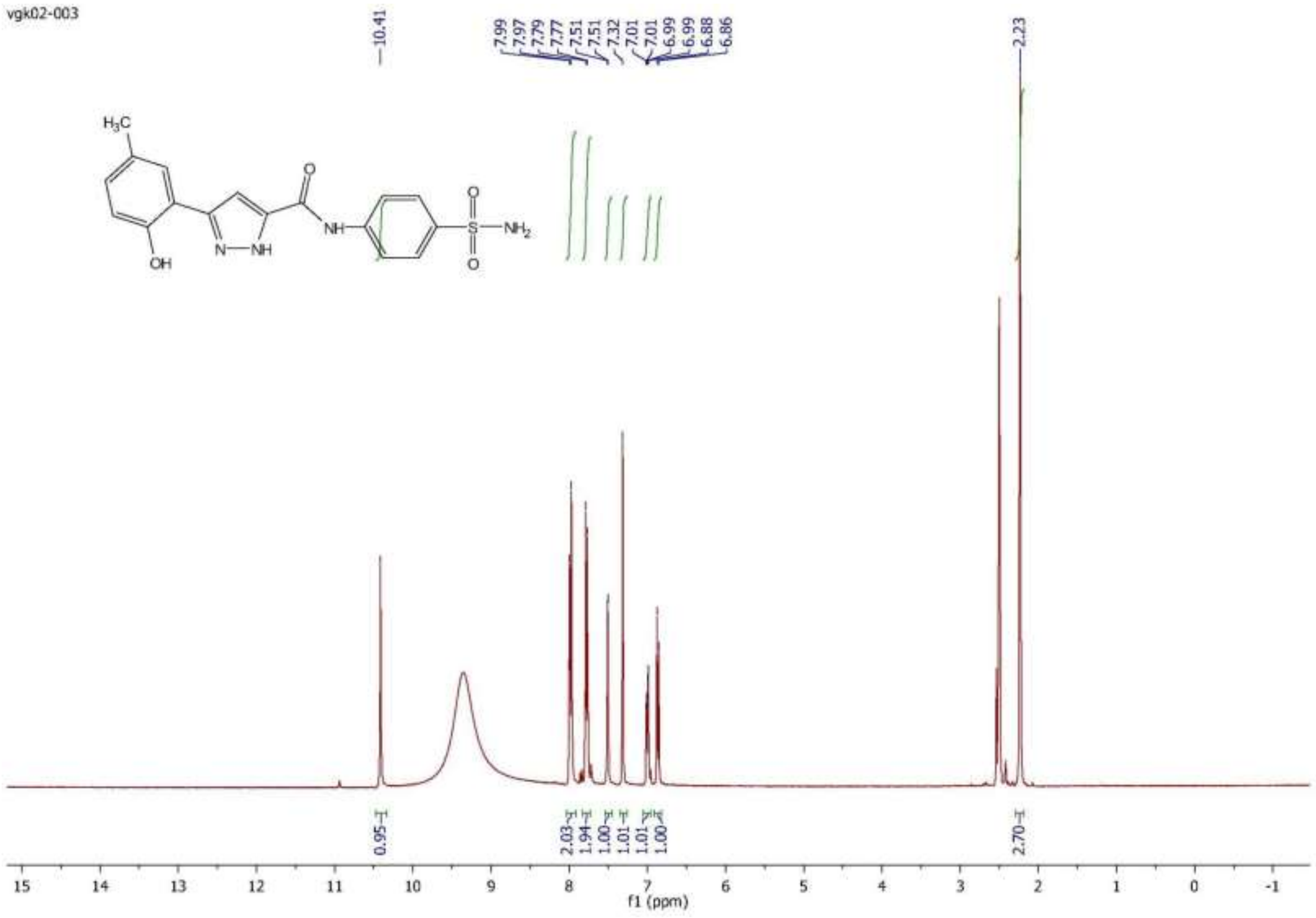

Figure S3B. ^{1}H NMR spectrum of *N-[4-(Aminosulfonyl)phenyl]-3-(2-hydroxy-5-methylphenyl)-1H-pyrazole-5-carboxamide* (**4c**) in DMSO-*d6*.+ 5%CF_3SO_3H.

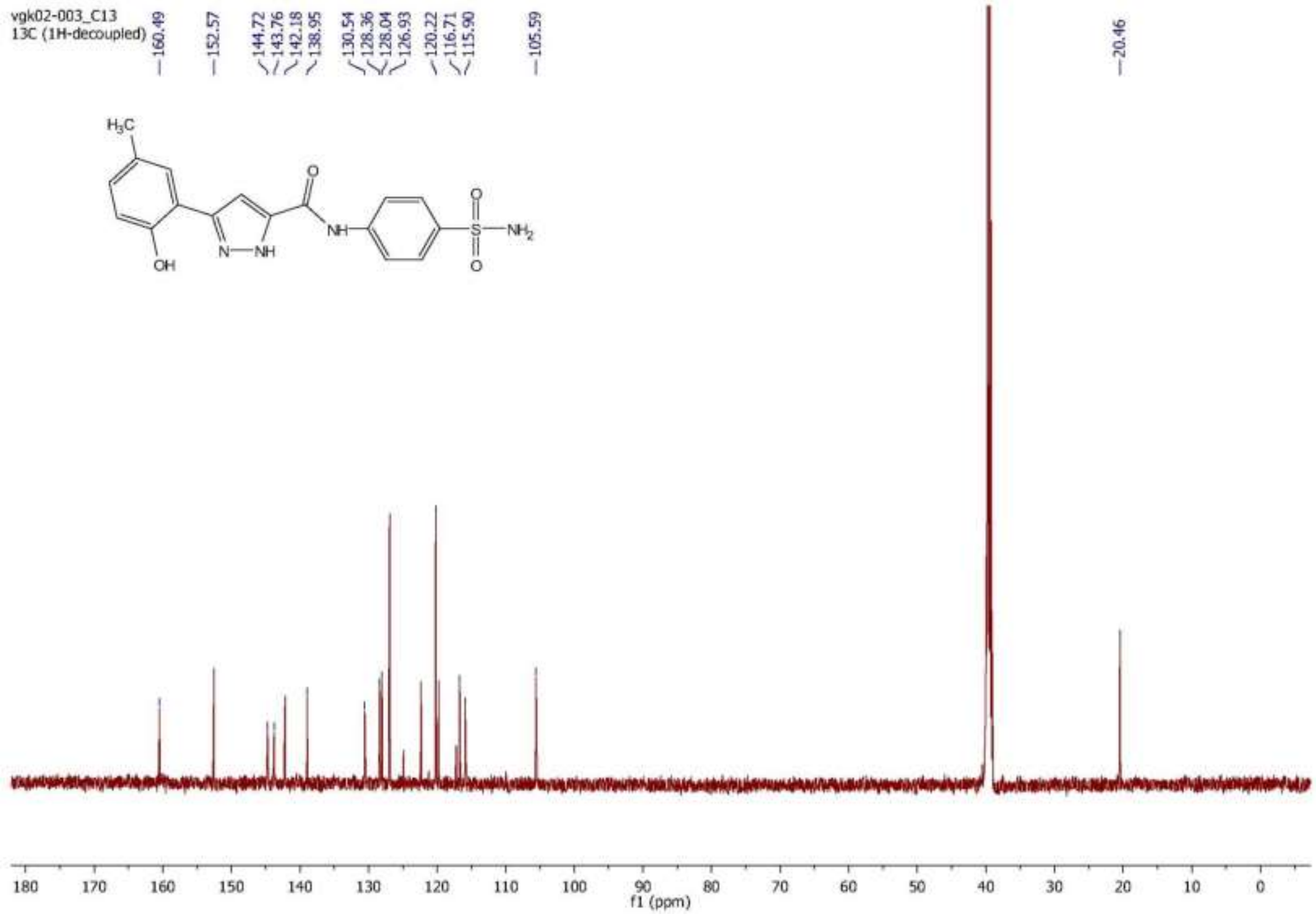

Figure S3C. ^{13}C NMR spectrum of *N-[4-(Aminosulfonyl)phenyl]-3-(2-hydroxy-5-methylphenyl)-1H-pyrazole-5-carboxamide* (**4c**) in DMSO-*d6*.+ 5%CF_3SO_3H.

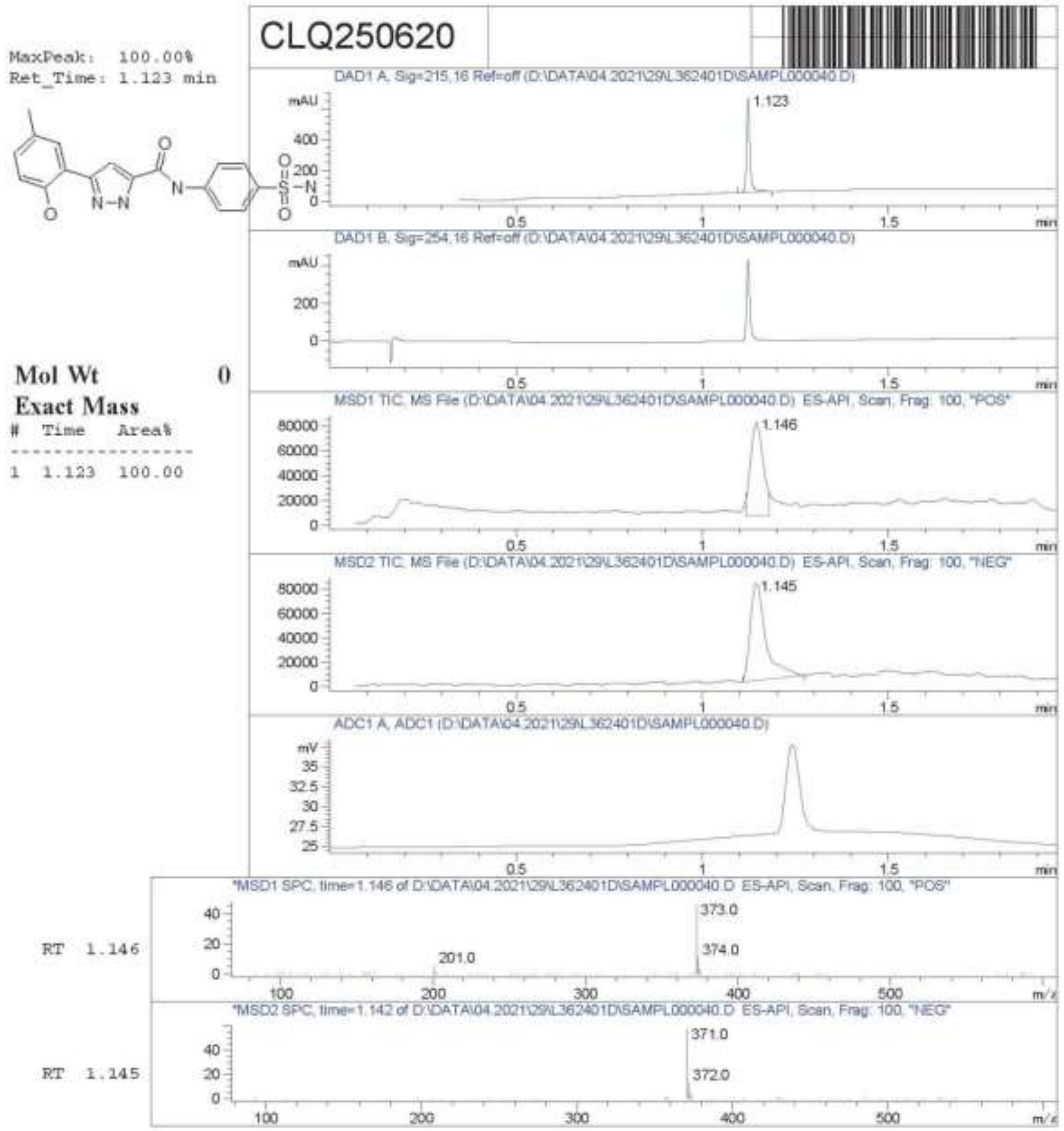

Inj.Date 4/29/2021 CH P2-E-06 - 4 - Acq. Method C:\CHEM32\-> ->

Figure S3D. LCMS spectrum of *N-[4-(Aminosulfonyl)phenyl]-3-(2-hydroxy-5-methylphenyl)-1H-pyrazole-5-carboxamide* (**4c**).

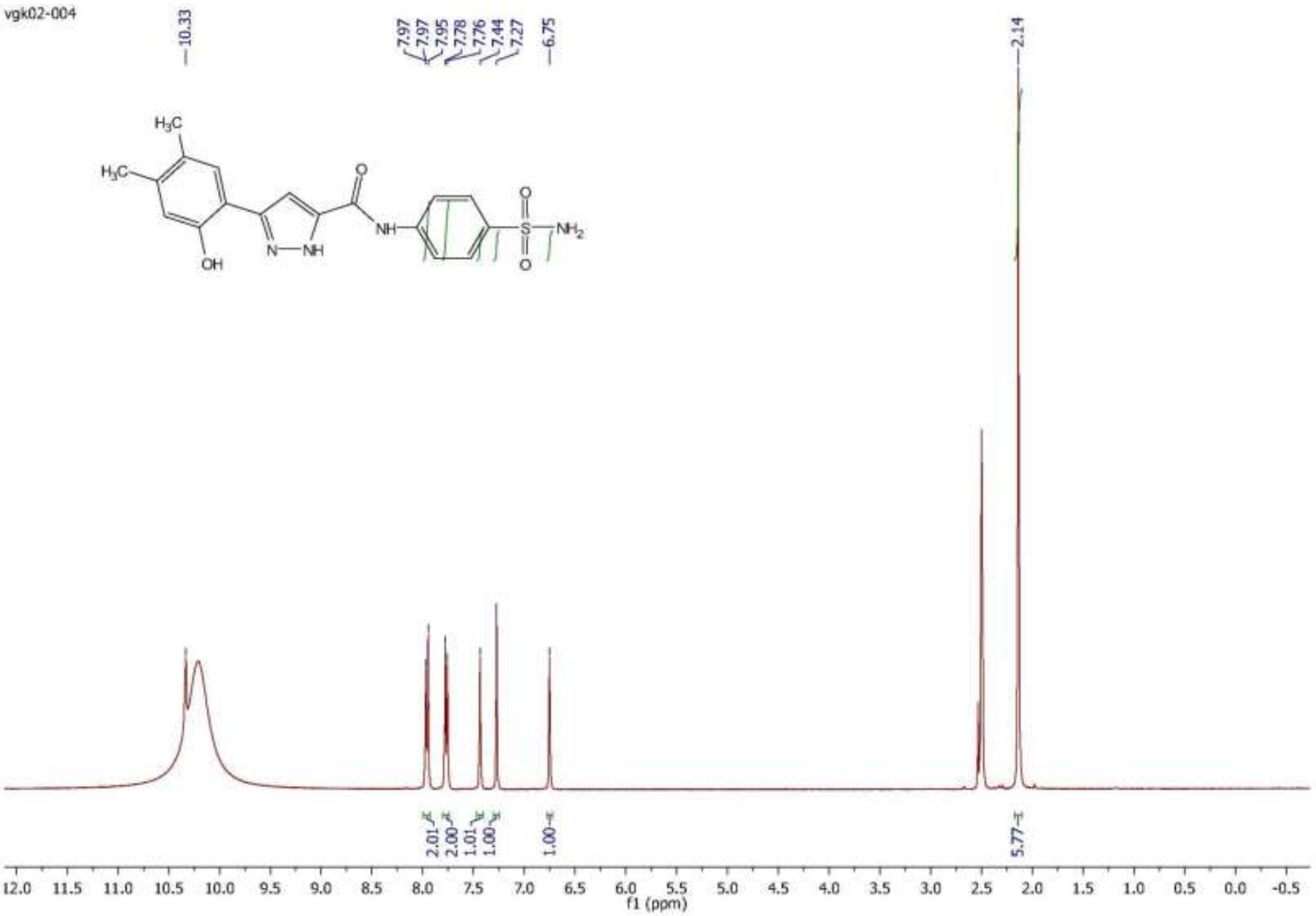

Figure S4B. ^{1}H NMR spectrum of *N-[4-(Aminosulfonyl)phenyl]-3-(2-hydroxy-4,5-dimethylphenyl)-1H-pyrazole-5-carboxamide* (**4d**) in DMSO-*d6*.+ 5%CF_3SO_3H.

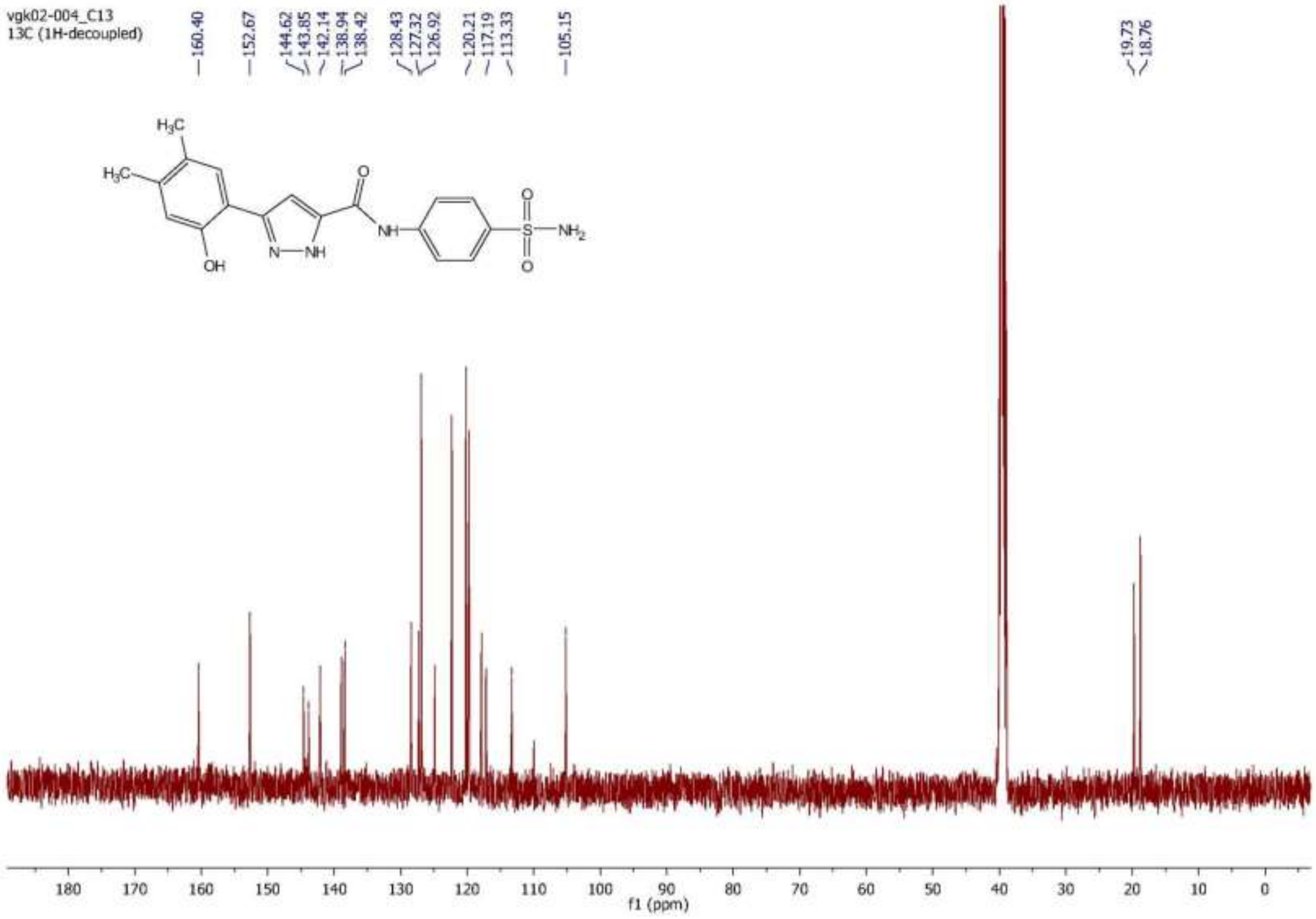

Figure S4B. ^{13}C NMR spectrum of *N-[4-(Aminosulfonyl)phenyl]-3-(2-hydroxy-4,5-dimethylphenyl)-1H-pyrazole-5-carboxamide* (**4d**) in DMSO-*d6*.+ 5%CF_3SO_3H.

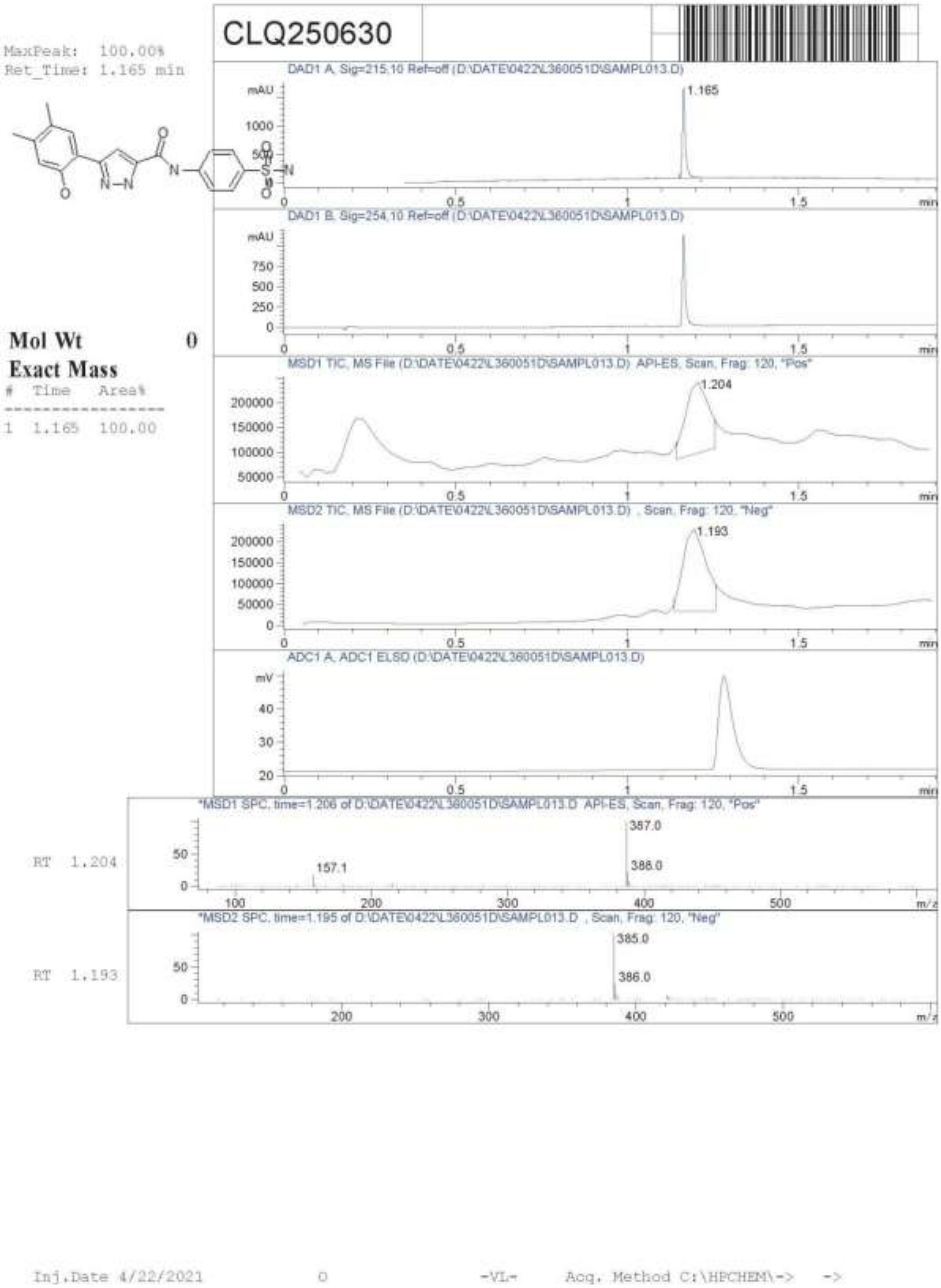

Figure S4D. LCMS spectrum of *N-[4-(Aminosulfonyl)phenyl]-3-(2-hydroxy-4,5-dimethylphenyl)-1H-pyrazole-5-carboxamide* (**4d**).

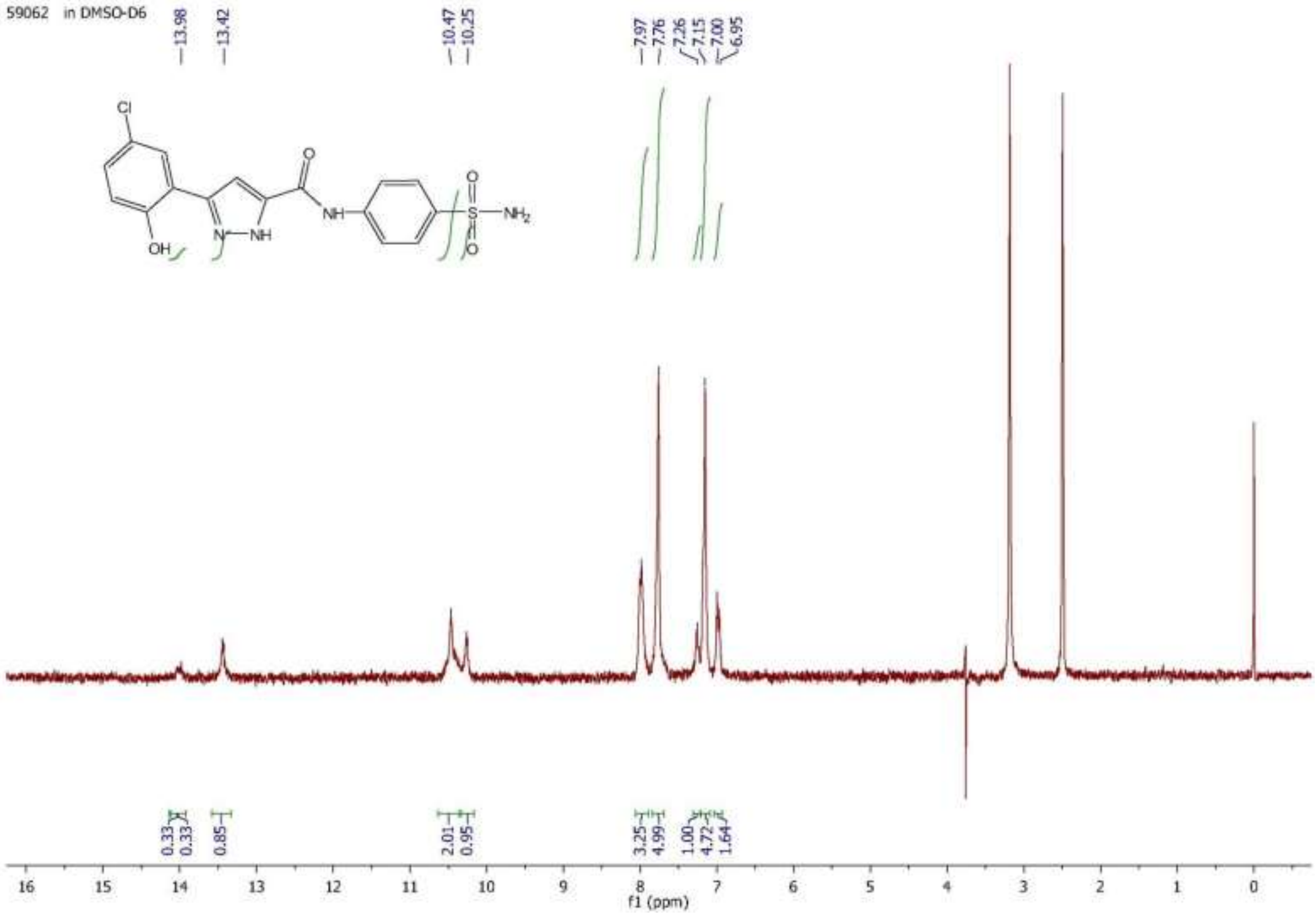

Figure S5A. ^{1}H NMR spectrum of *N-[4-(Aminosulfonyl)phenyl]-3-(5-chloro-2-hydroxyphenyl)-1H-pyrazole-5-carboxamide* (**4f**) in DMSO-*d6*..

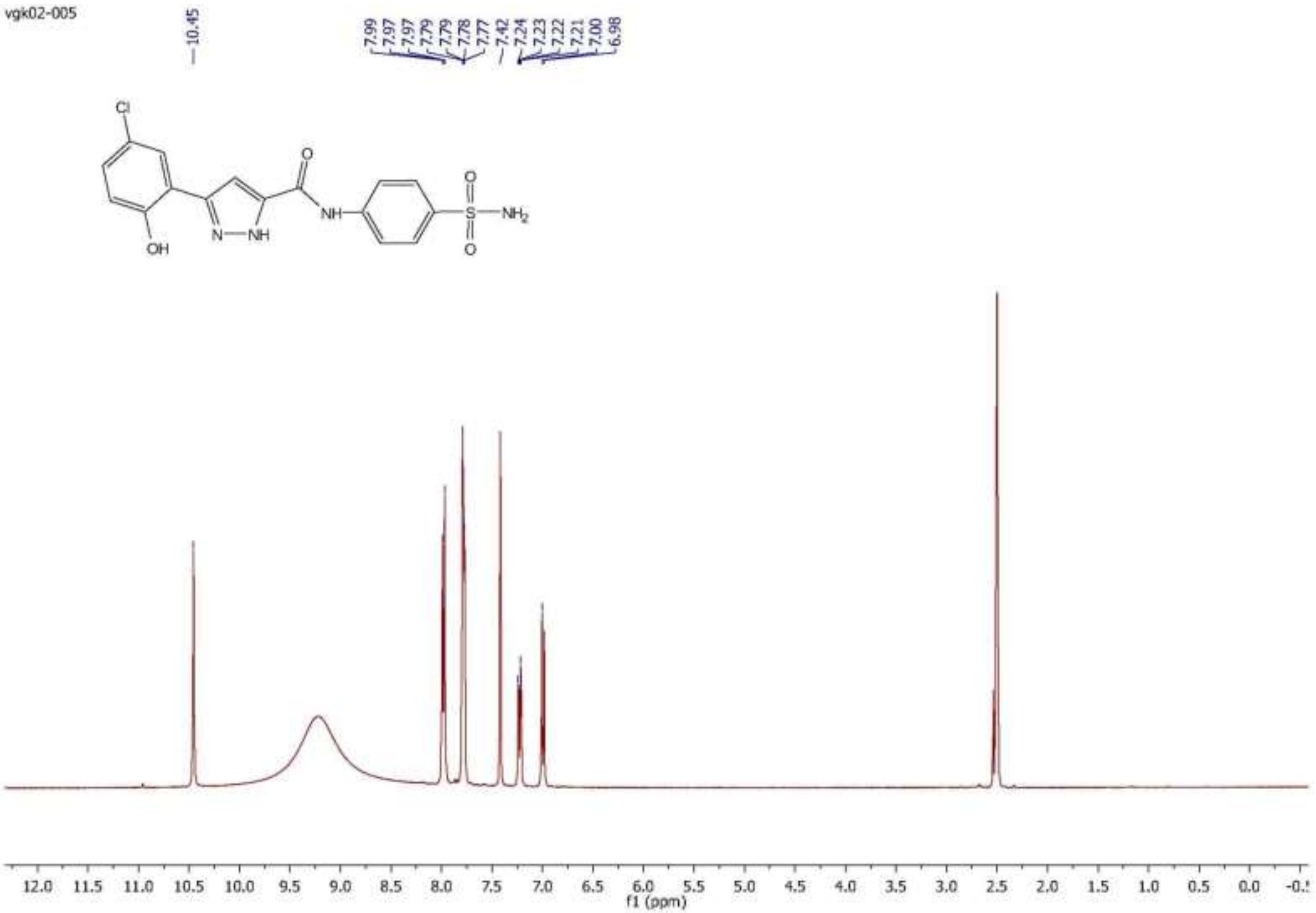

Figure S5B. ^{1}H NMR spectrum of *N-[4-(Aminosulfonyl)phenyl]-3-(5-chloro-2-hydroxyphenyl)-1H-pyrazole-5-carboxamide* (**4f**) in DMSO-*d6*.+ 5%CF_3SO_3H

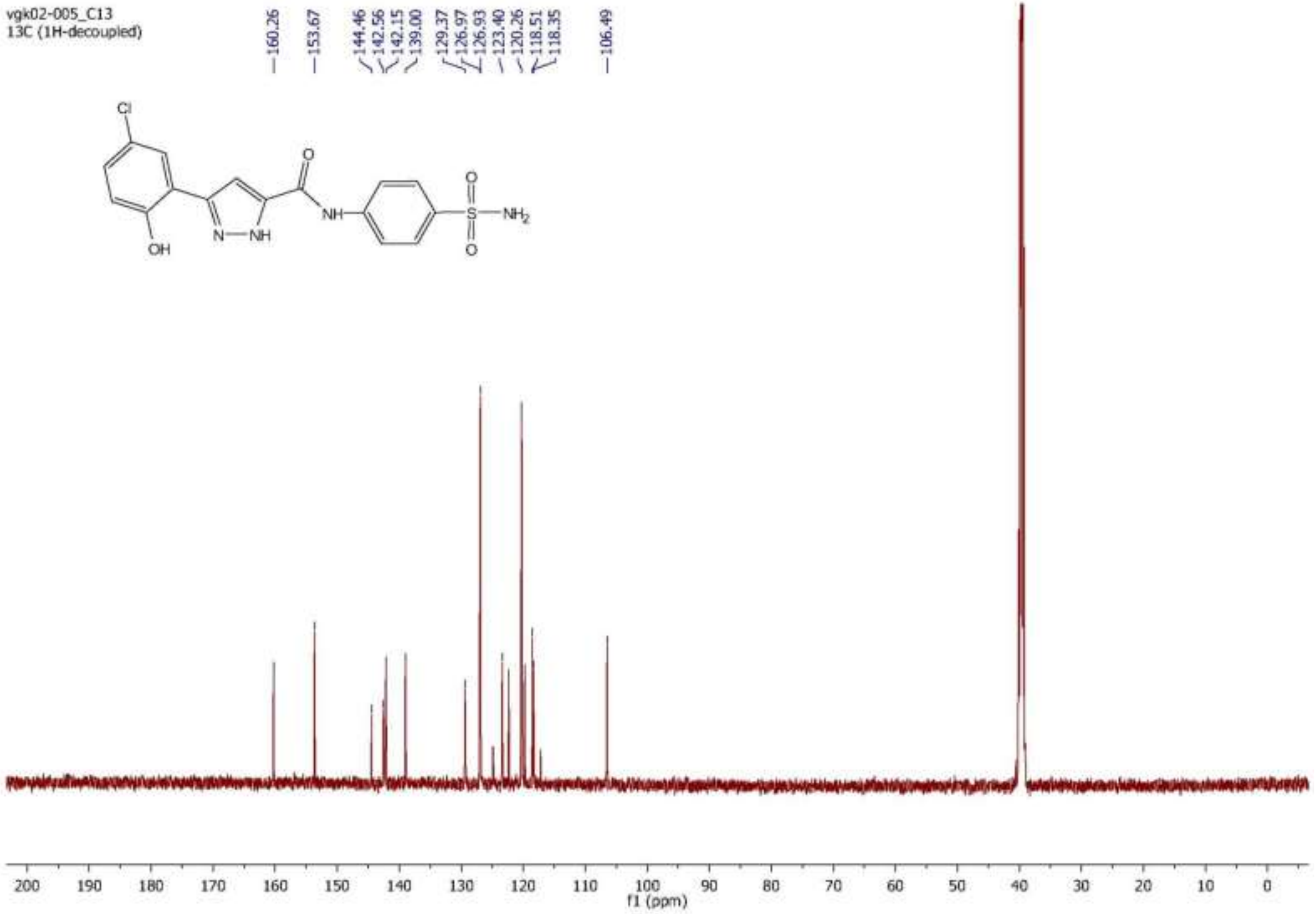

Figure S5C. ^{13}CNMR spectrum of *N-[4-(Aminosulfonyl)phenyl]-3-(5-chloro-2-hydroxyphenyl)-1H-pyrazole-5-carboxamide* (**4f**) in DMSO-*d6*.+ 5%CF_3SO_3H

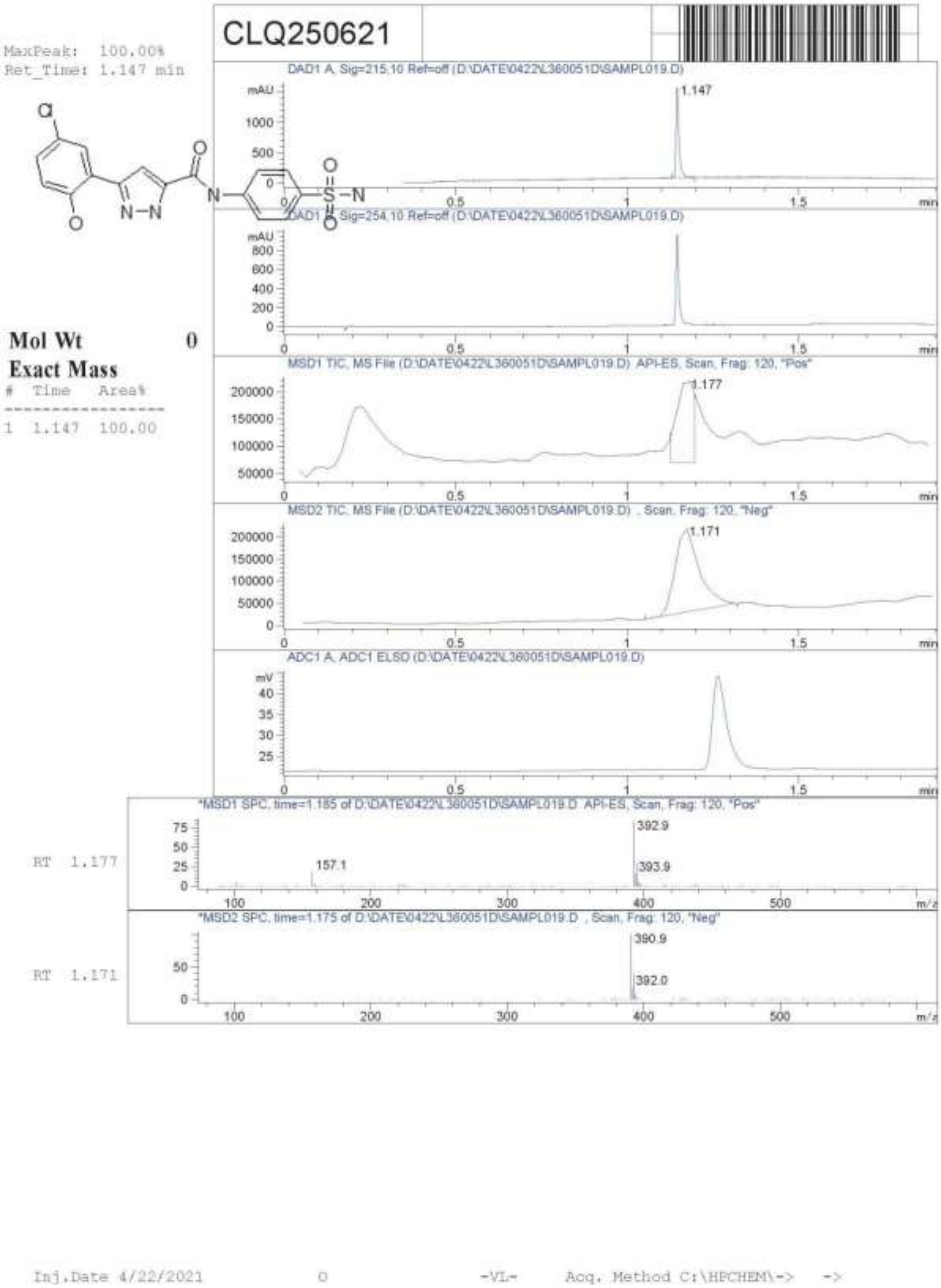

Figure S5D. LCMS spectrum of *N-[4-(Aminosulfonyl)phenyl]-3-(5-chloro-2-hydroxyphenyl)-1H-pyrazole-5-carboxamide* (**4f**).

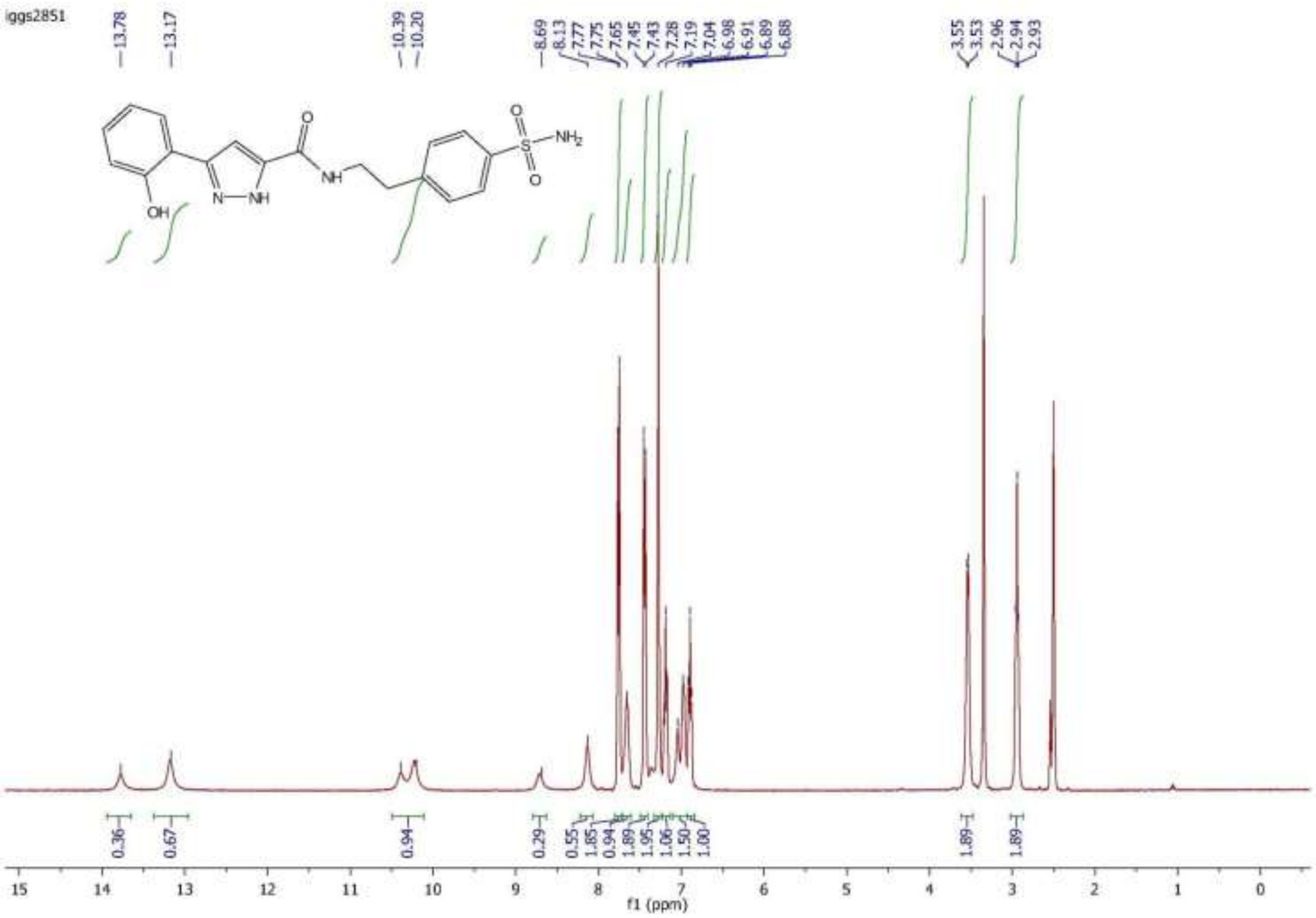

Figure S6A. ^{1}H NMR spectrum of *N-{2-[4-(Aminosulfonyl)phenyl]ethyl}-3-(2-hydroxyphenyl)-1H-pyrazole-5-carboxamide* (**5a**) in DMSO-*d6*.

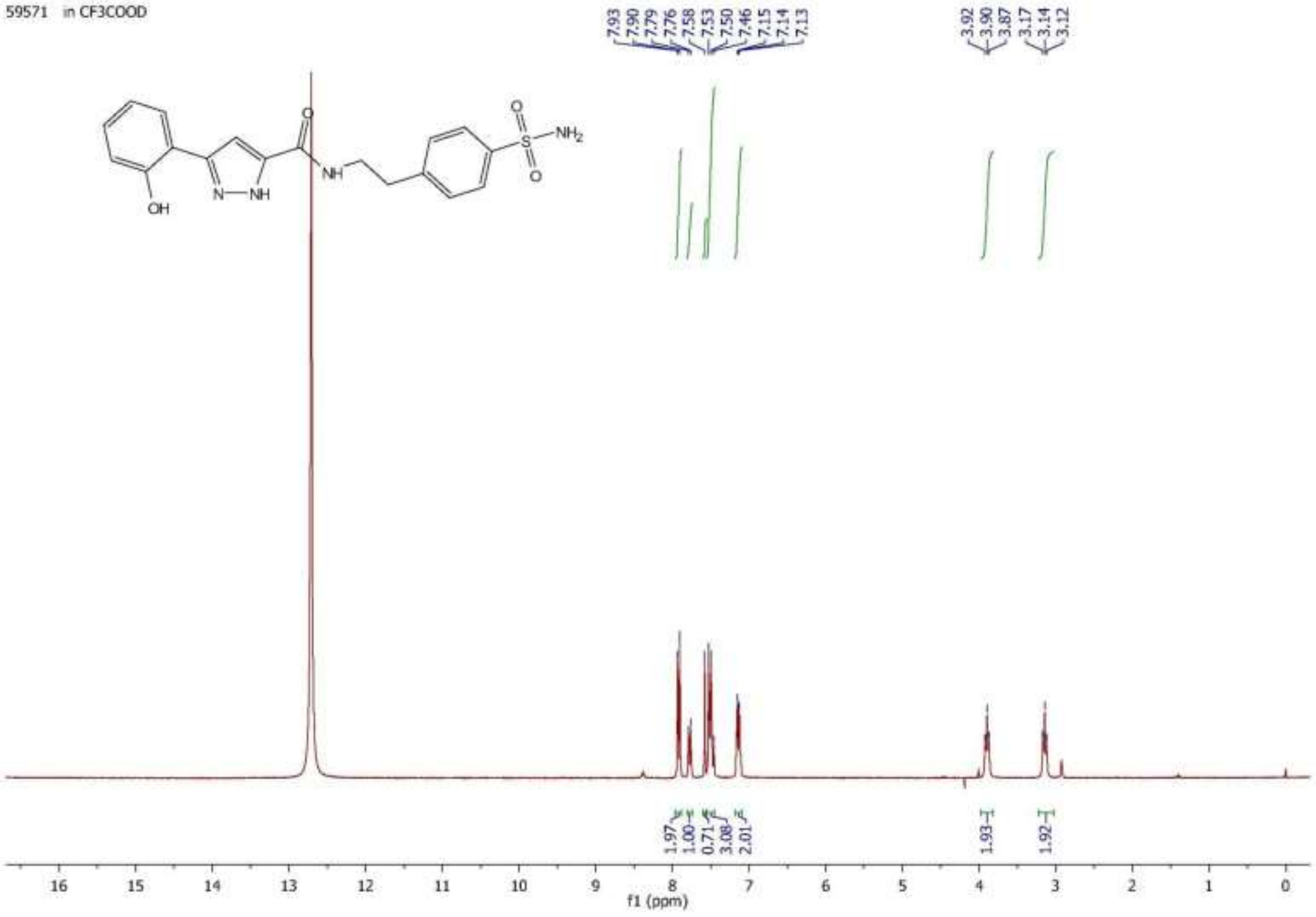

Figure S6B. ^{1}H NMR spectrum of *N-{2-[4-(Aminosulfonyl)phenyl]ethyl}-3-(2-hydroxyphenyl)-1H-pyrazole-5-carboxamide* (**5a**) in CF_3COOD.

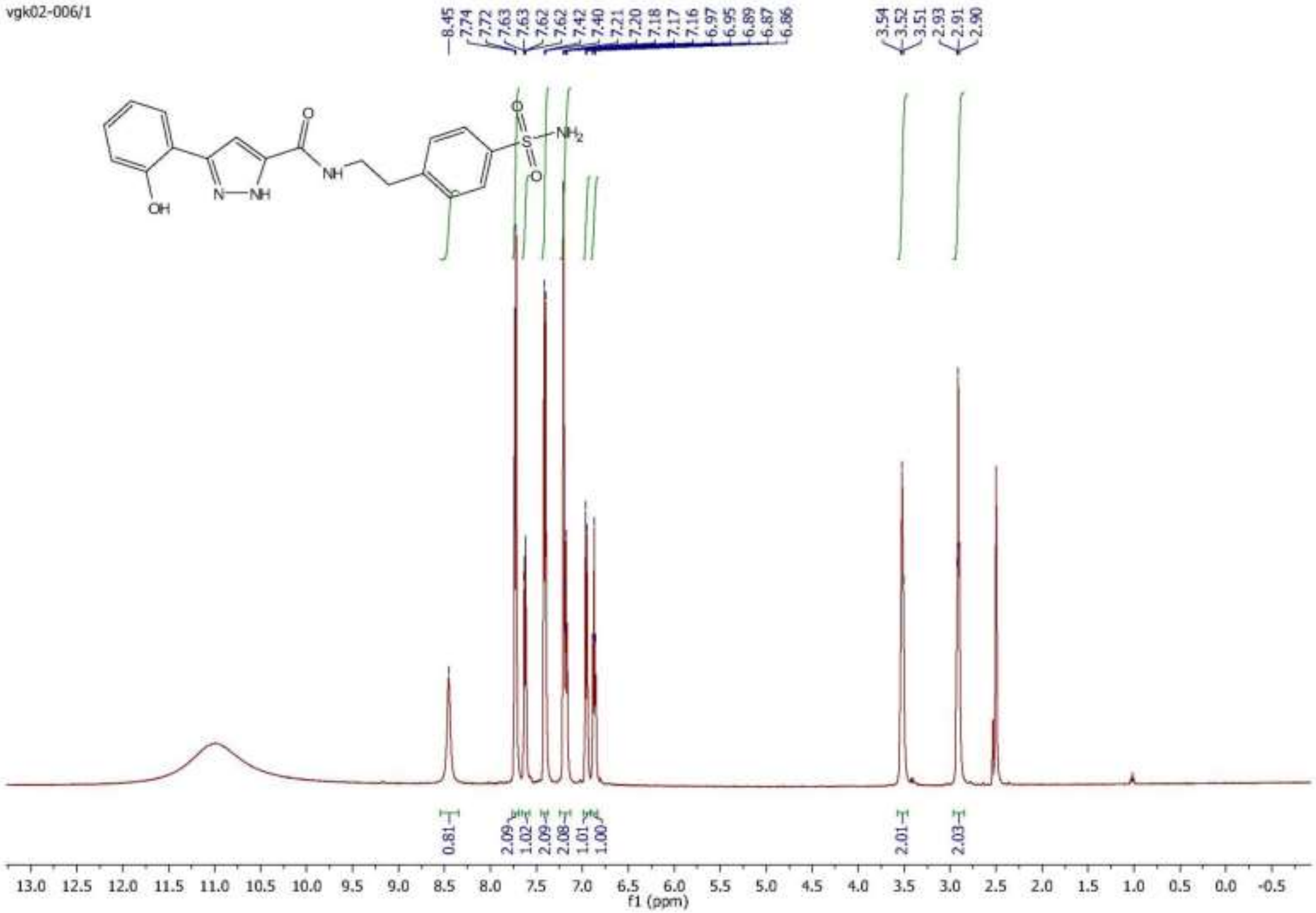

Figure S6C. ^{1}H NMR spectrum of *N-{2-[4-(Aminosulfonyl)phenyl]ethyl}-3-(2-hydroxyphenyl)-1H-pyrazole-5-carboxamide* (**5a**) in DMSO-*d6*.+ 5%CF_3SO_3H

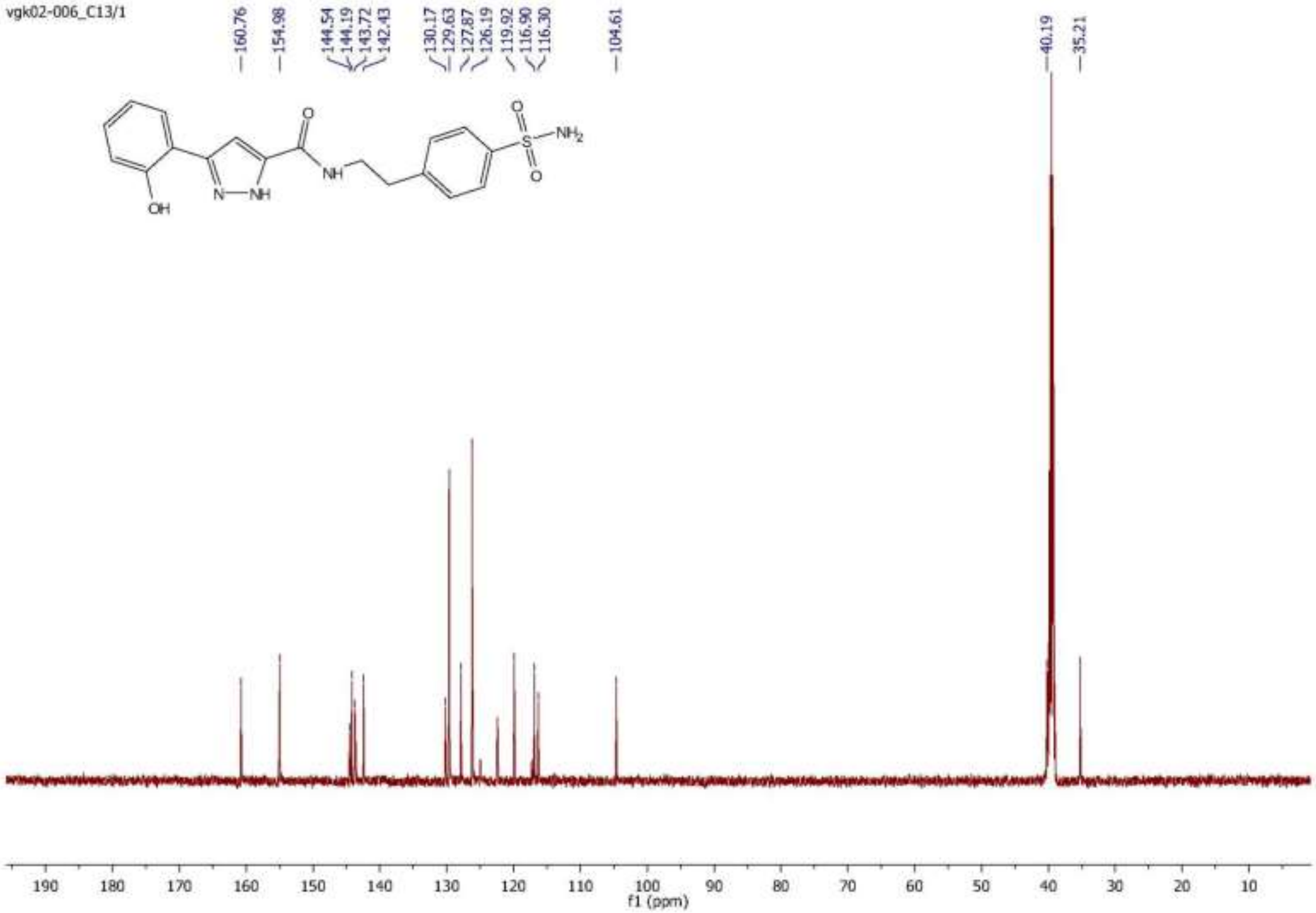

Figure S6D. ^{13}C NMR spectrum of *N-{2-[4-(Aminosulfonyl)phenyl]ethyl}-3-(2-hydroxyphenyl)-1H-pyrazole-5-carboxamide* (**5a**) in DMSO-*d6*.+ 5%CF_3SO_3H

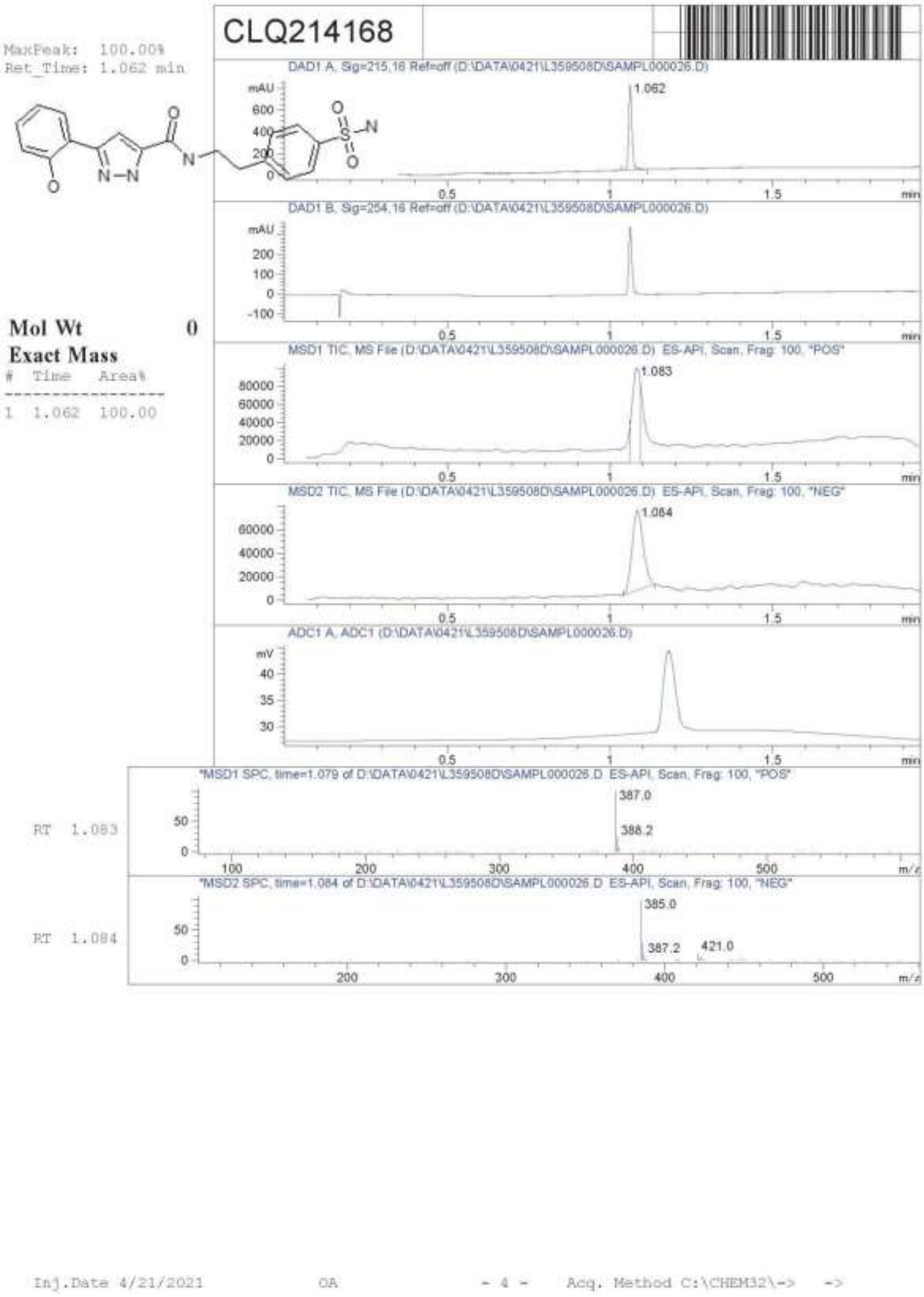

Figure S6F. LCMS spectrum of *N-{2-[4-(Aminosulfonyl)phenyl]ethyl}-3-(2-hydroxyphenyl)-1H-pyrazole-5-carboxamide* (**5a**).

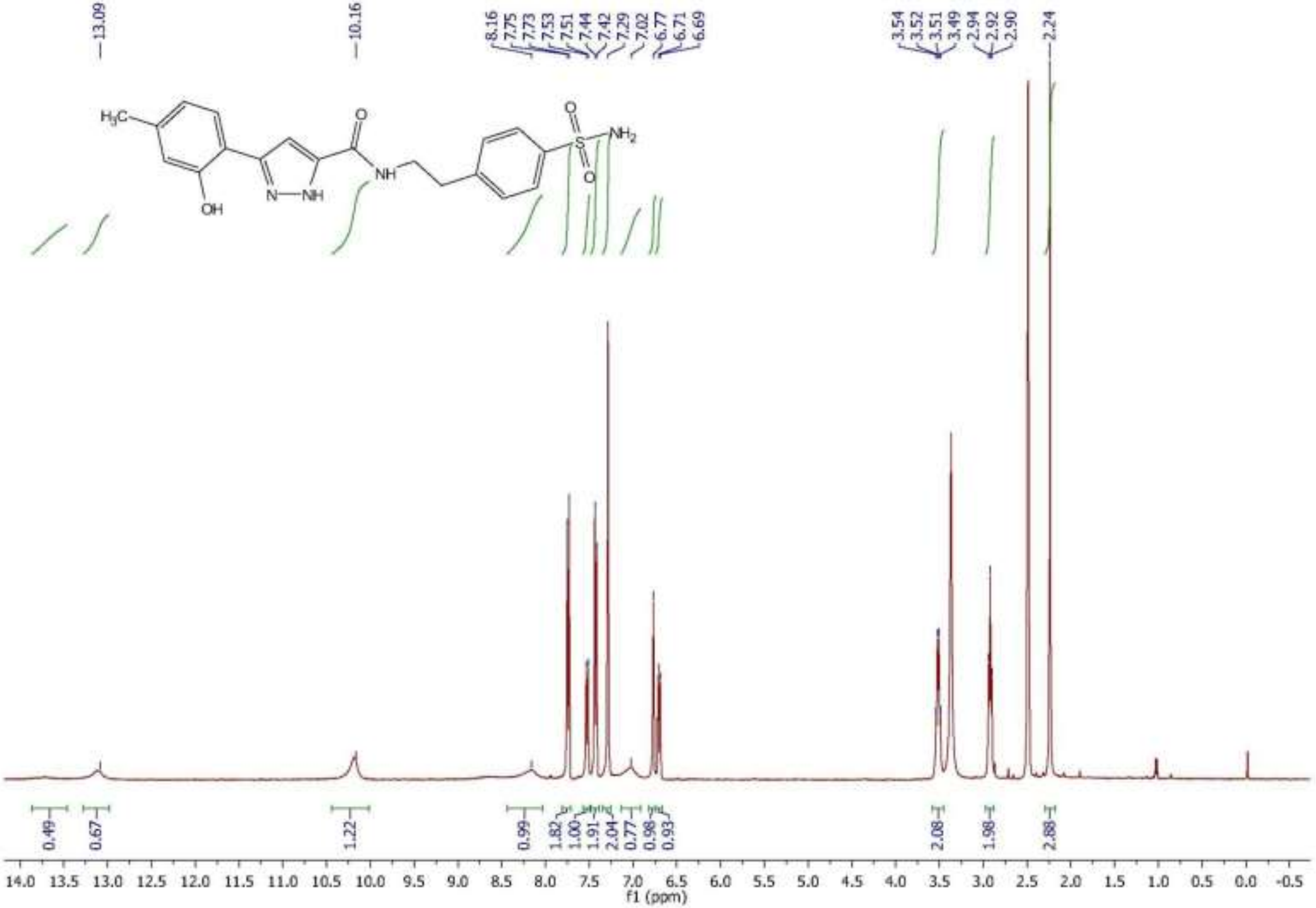

Figure S7A. ^{1}H NMR spectrum of *N-{2-[4-(Aminosulfonyl)phenyl]ethyl}-3-(2-hydroxy-4-methylphenyl)-1H-pyrazole-5-carboxamide* (**5b**) in DMSO-*d6*.

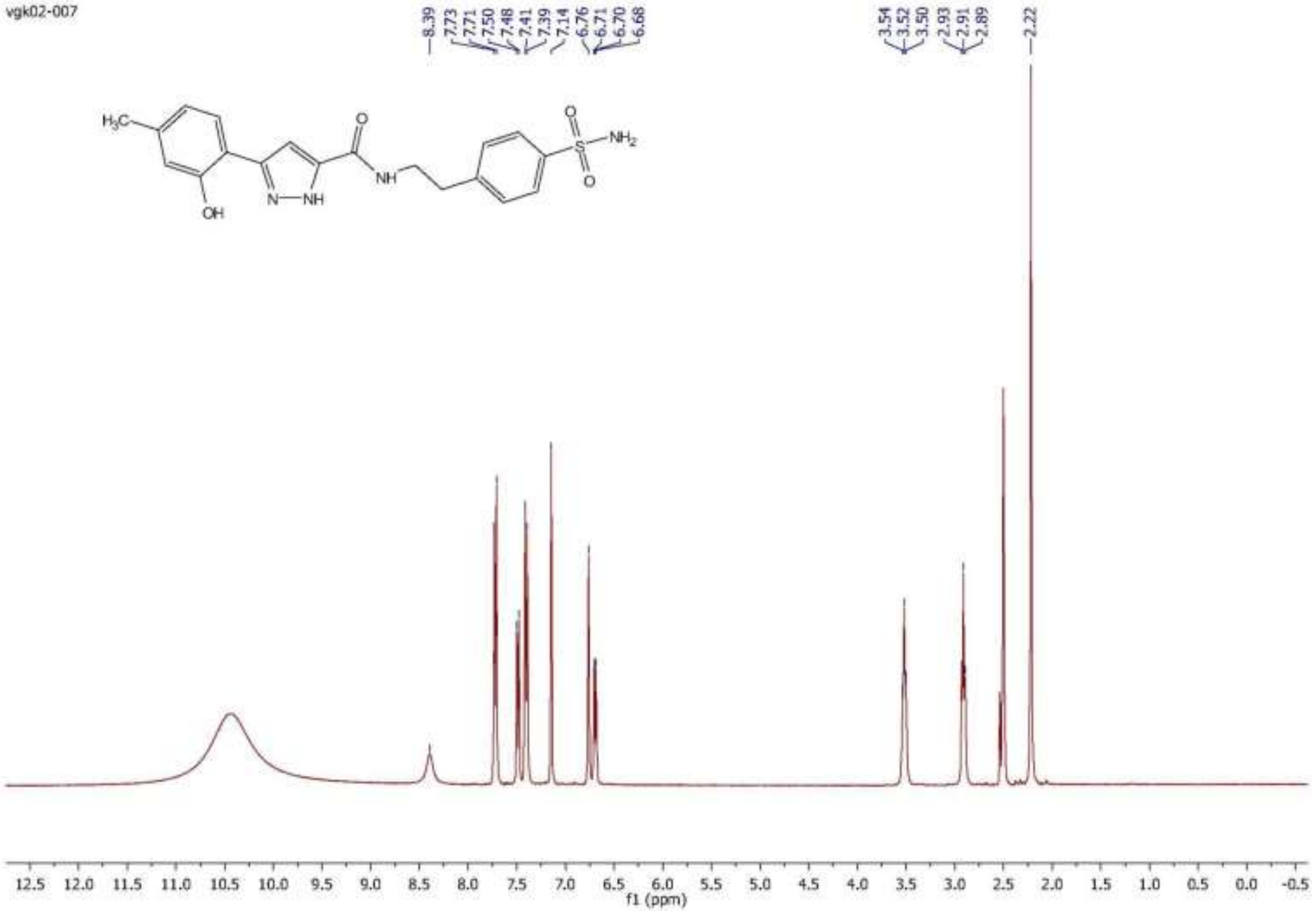

Figure S7B. ^{1}H NMR spectrum of *N-{2-[4-(Aminosulfonyl)phenyl]ethyl}-3-(2-hydroxy-4-methylphenyl)-1H-pyrazole-5-carboxamide* (**5b**) in DMSO-*d6*.+ 5%CF_3SO_3H.

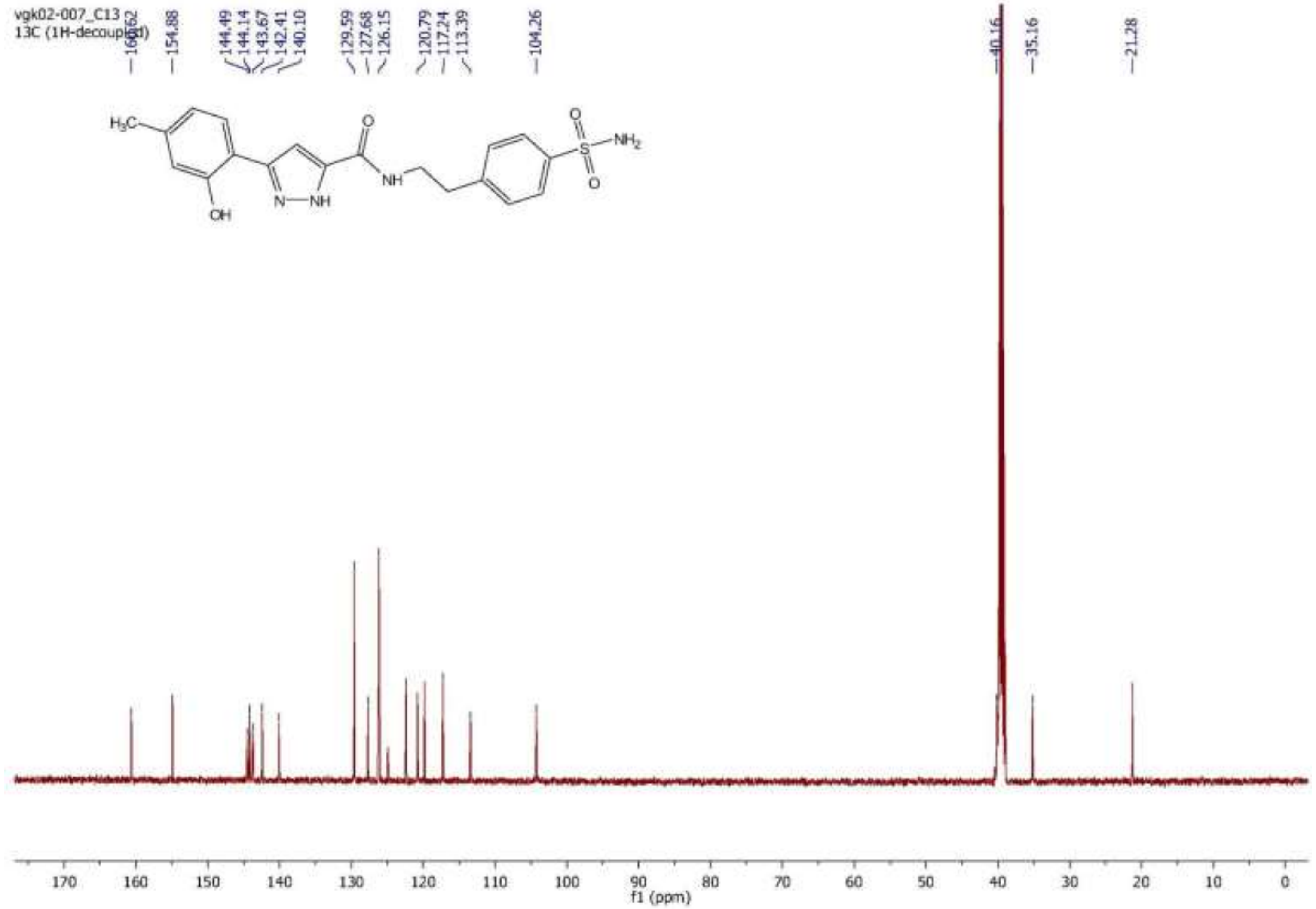

Figure S7C. ^{13}C NMR spectrum of *N-{2-[4-(Aminosulfonyl)phenyl]ethyl}-3-(2-hydroxy-4-methylphenyl)-1H-pyrazole-5-carboxamide* (**5b**) in DMSO-*d6*.+ 5%CF_3SO_3H.

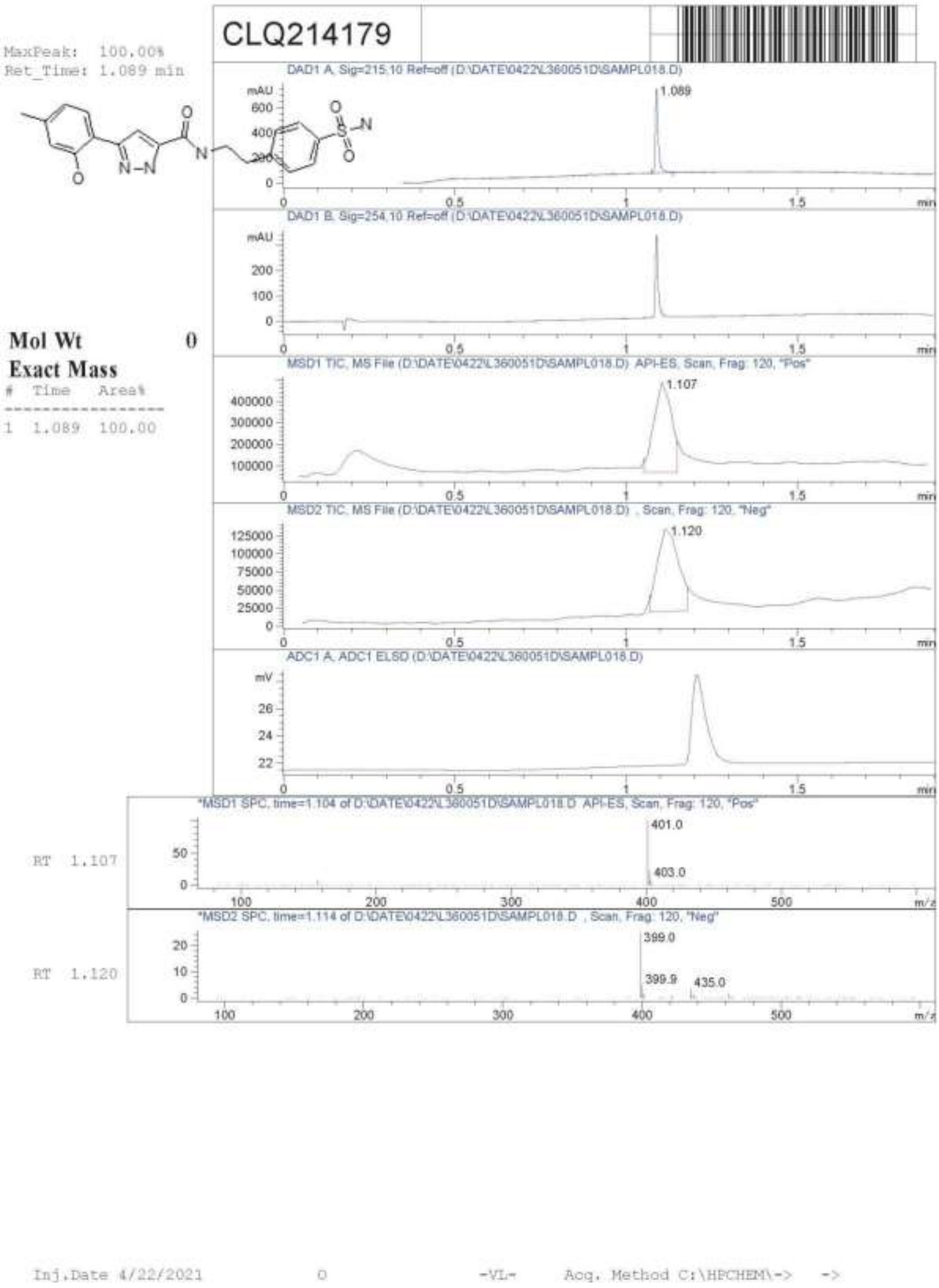

Figure S7D. LCMS spectrum of *N-{2-[4-(Aminosulfonyl)phenyl]ethyl}-3-(2-hydroxy-4-methylphenyl)-1H-pyrazole-5-carboxamide* (**5b**).

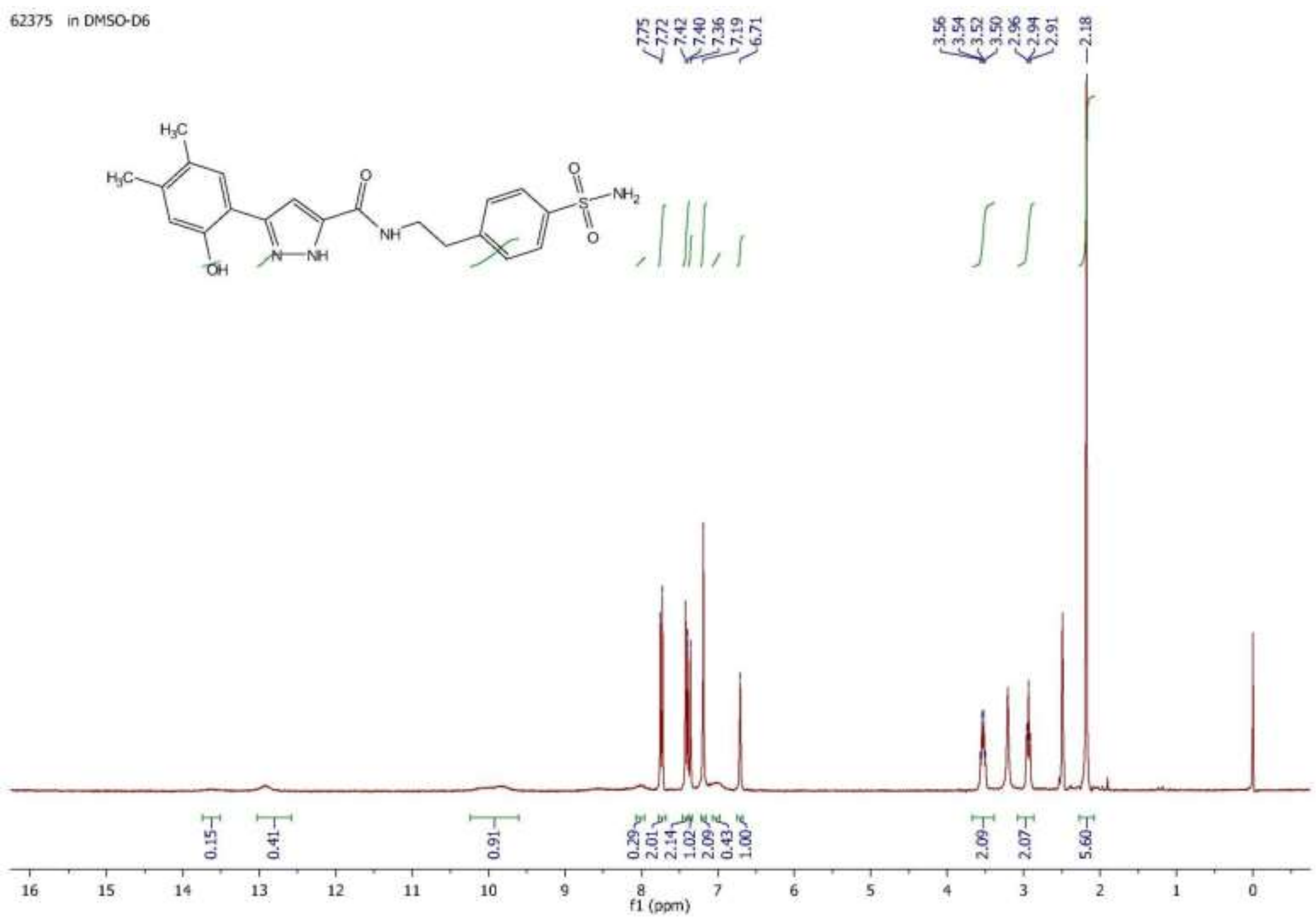

Figure S8A. ^{1}H NMR spectrum of *N-{2-[4-(Aminosulfonyl)phenyl]ethyl}-3-(2-hydroxy-4,5-dimethylphenyl)-1H-pyrazole-5-carboxamide* (**5d)** in DMSO-*d6*.

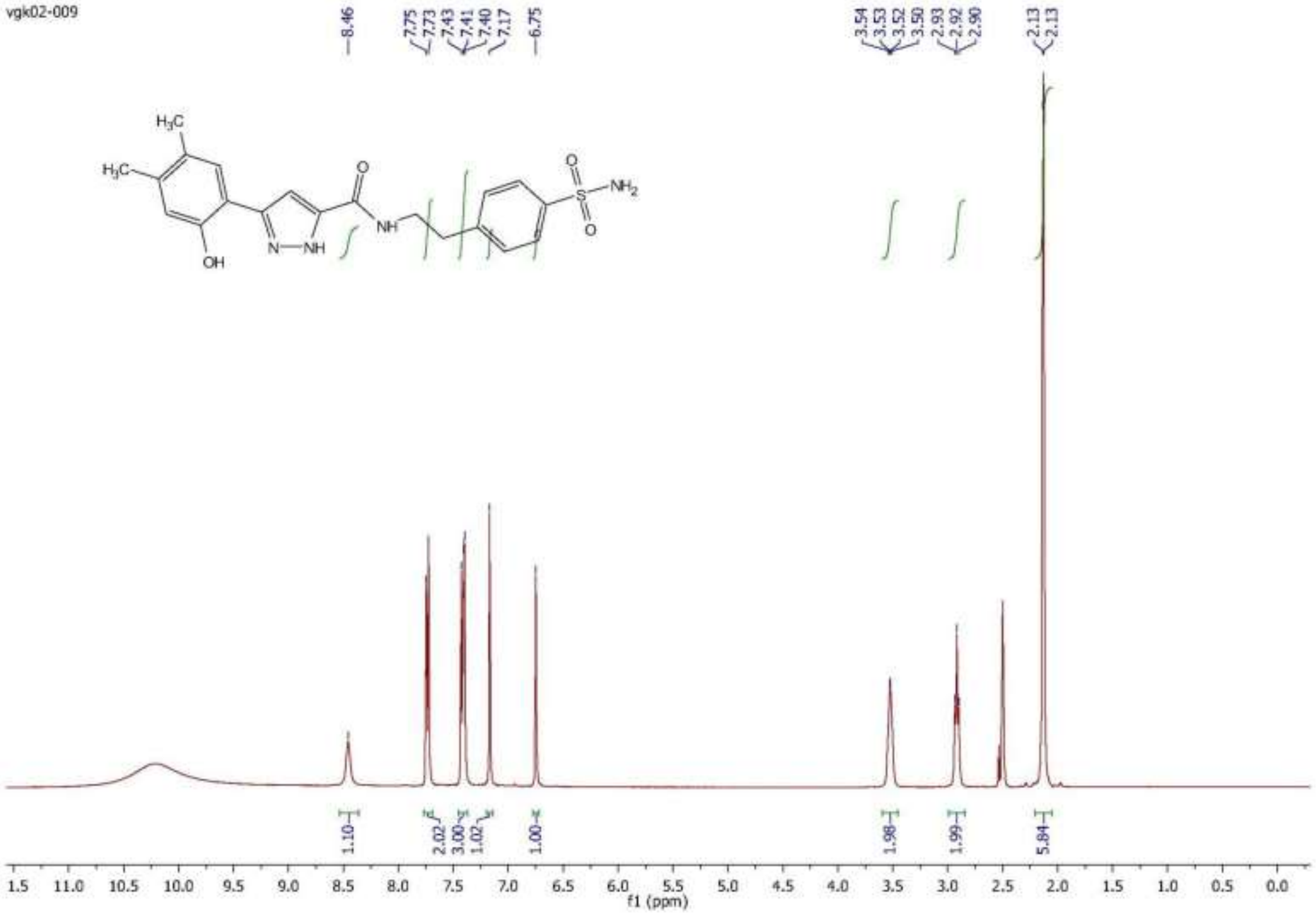

Figure S8B. ^{1}H NMR spectrum of *N-{2-[4-(Aminosulfonyl)phenyl]ethyl}-3-(2-hydroxy-4,5-dimethylphenyl)-1H-pyrazole-5-carboxamide* (**5d)** in DMSO-*d6*.+ 5%CF_3SO_3H.

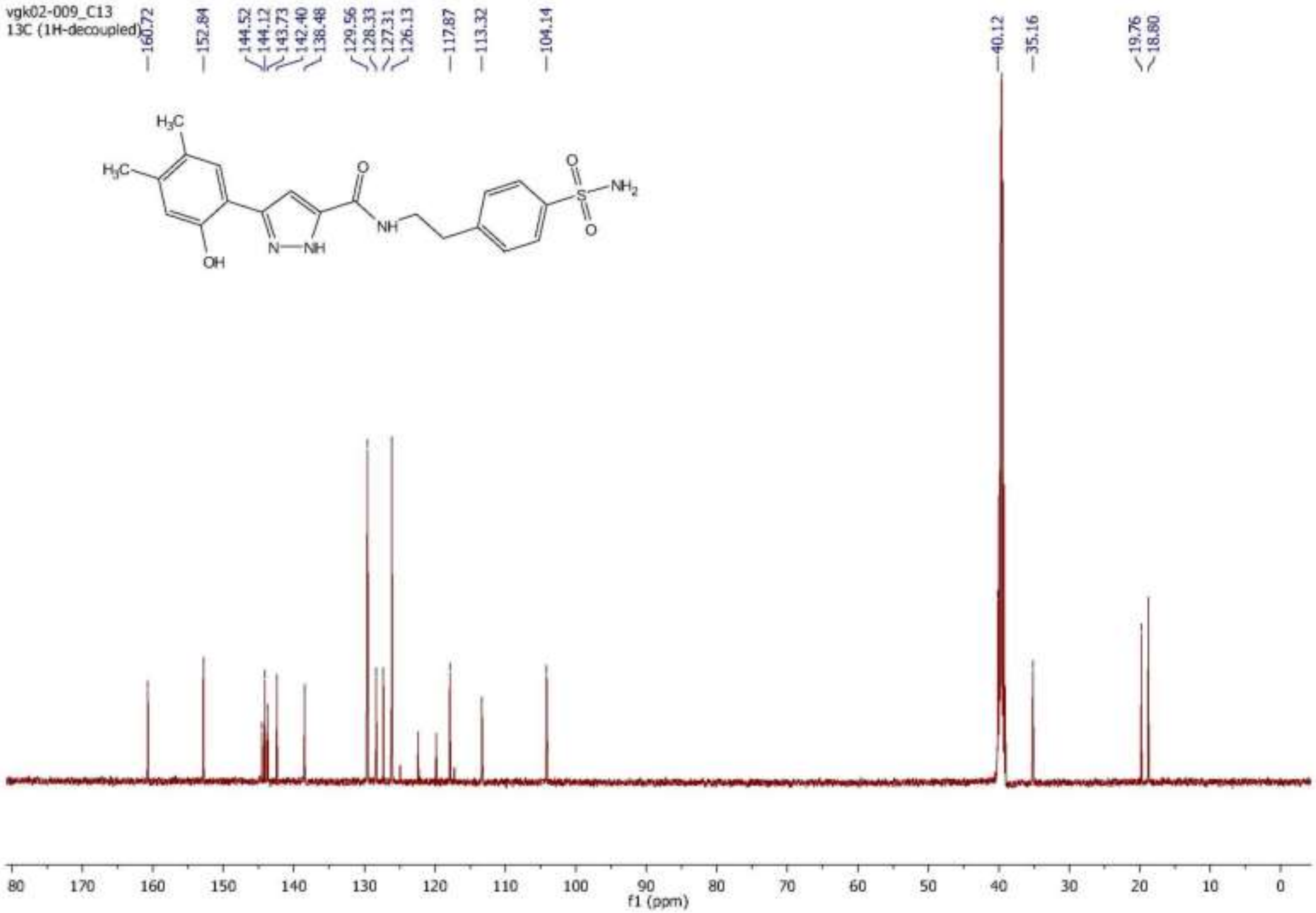

Figure S8C. ^{13}C NMR spectrum of *N-{2-[4-(Aminosulfonyl)phenyl]ethyl}-3-(2-hydroxy-4,5-dimethylphenyl)-1H-pyrazole-5-carboxamide* (**5d)** in DMSO-*d6*.+ 5%CF_3SO_3H.

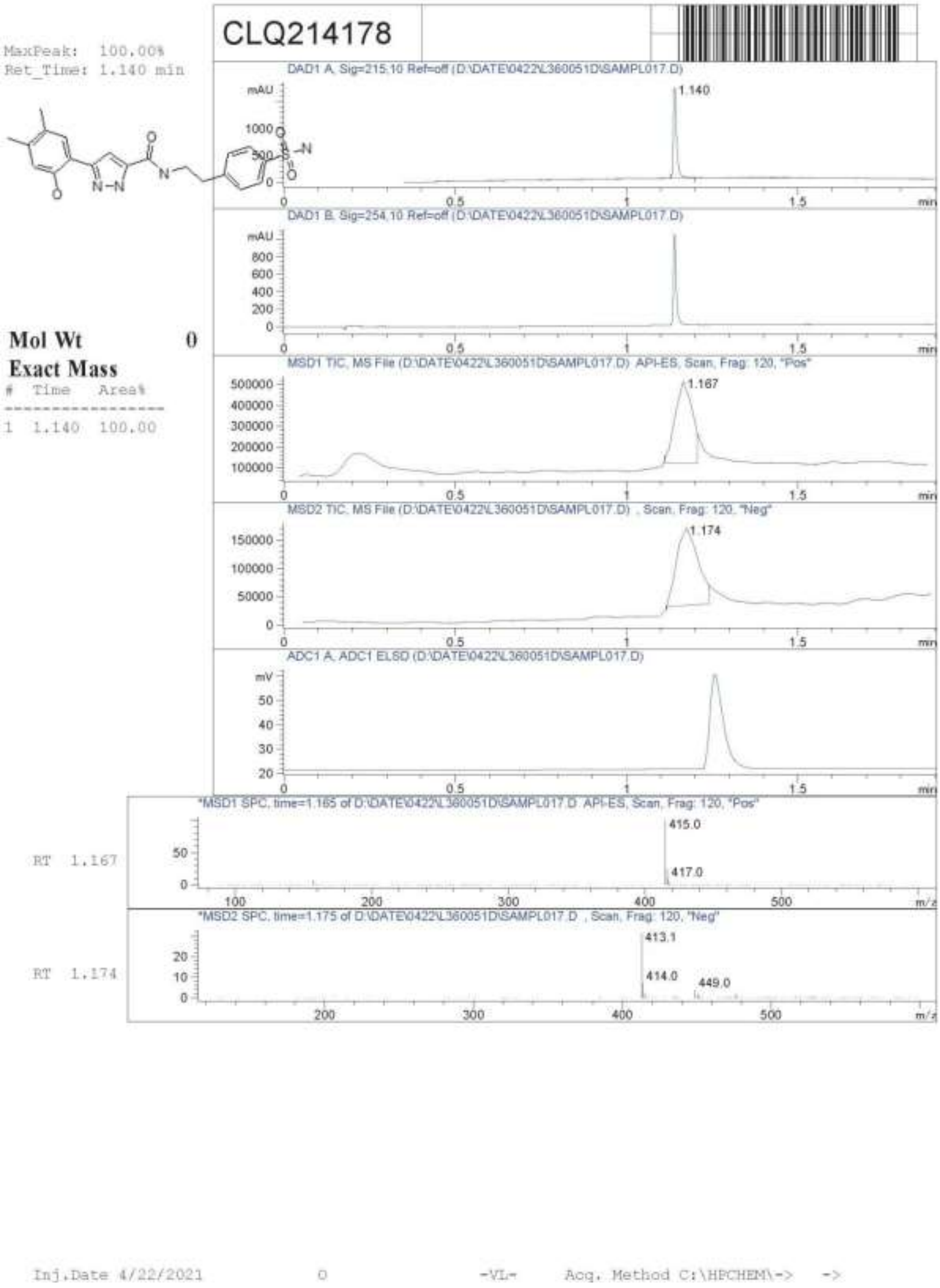

Figure S8D. LCMS spectrum of *N-{2-[4-(Aminosulfonyl)phenyl]ethyl}-3-(2-hydroxy-4,5-dimethylphenyl)-1H-pyrazole-5-carboxamide* (**5d**).

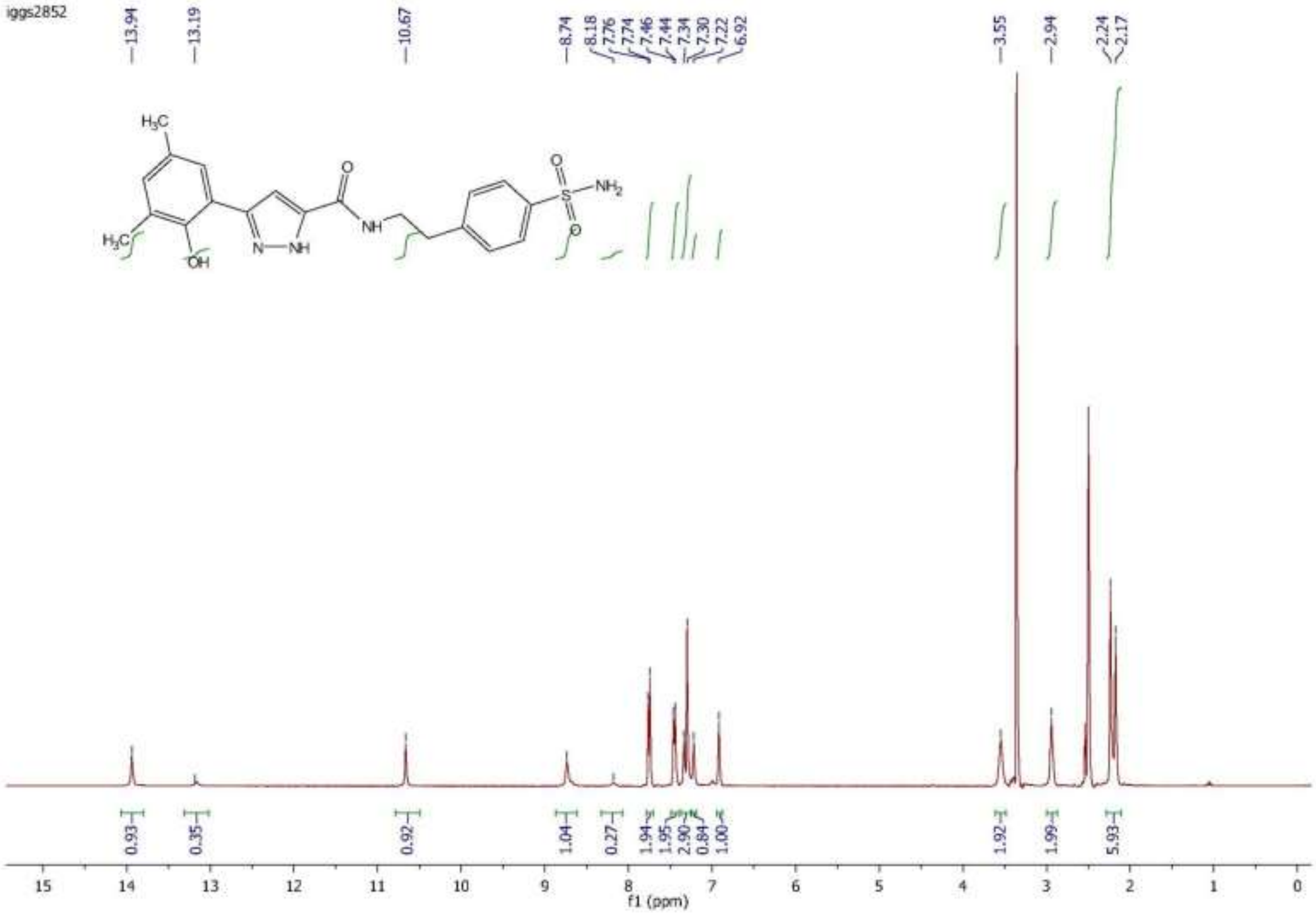

Figure S9A. ^{1}H NMR spectrum of *N-{2-[4-(Aminosulfonyl)phenyl]ethyl}-3-(2-hydroxy-3,5-dimethylphenyl)-1H-pyrazole-5-carboxamide* (**5e**) in DMSO-*d6*.

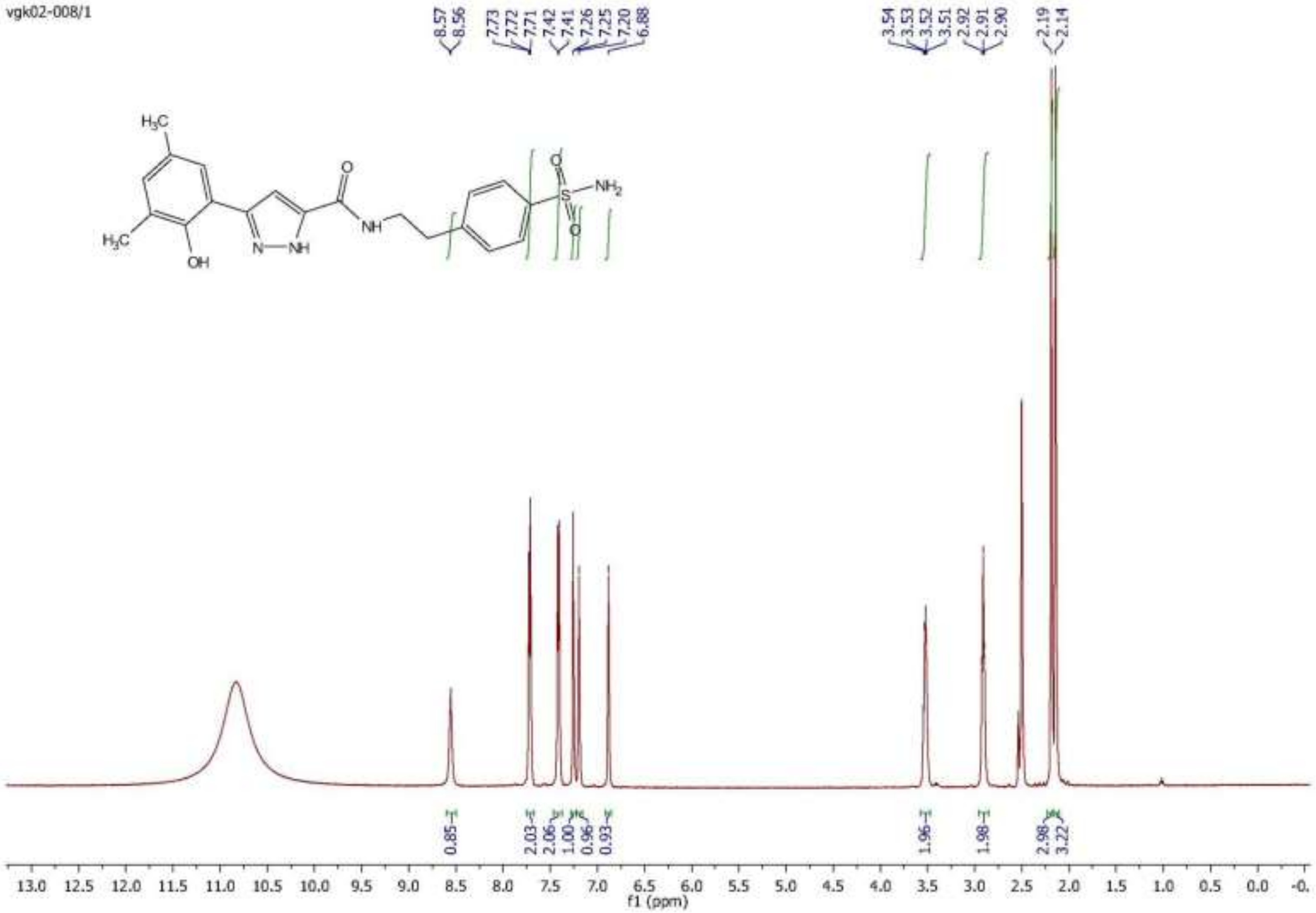

Figure S9B. ^{1}H NMR spectrum of *N-{2-[4-(Aminosulfonyl)phenyl]ethyl}-3-(2-hydroxy-3,5-dimethylphenyl)-1H-pyrazole-5-carboxamide* (**5e)** in DMSO-*d6*.+ 5%CF_3SO_3H.

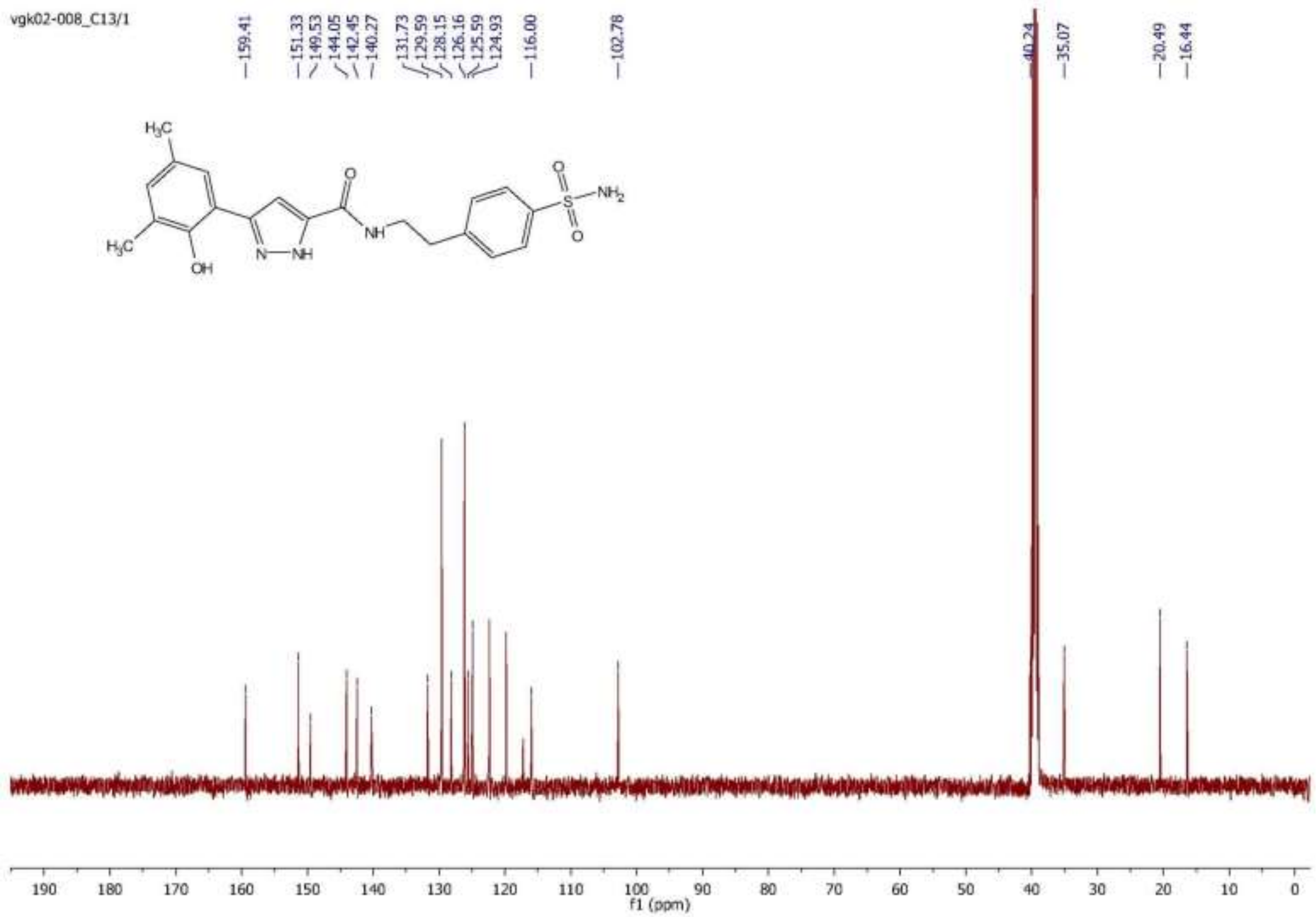

Figure S9C ^{13}C NMR spectrum of *N-{2-[4-(Aminosulfonyl)phenyl]ethyl}-3-(2-hydroxy-3,5-dimethylphenyl)-1H-pyrazole-5-carboxamide* (**5e)** in DMSO-*d6*.+ 5%CF_3SO_3H.

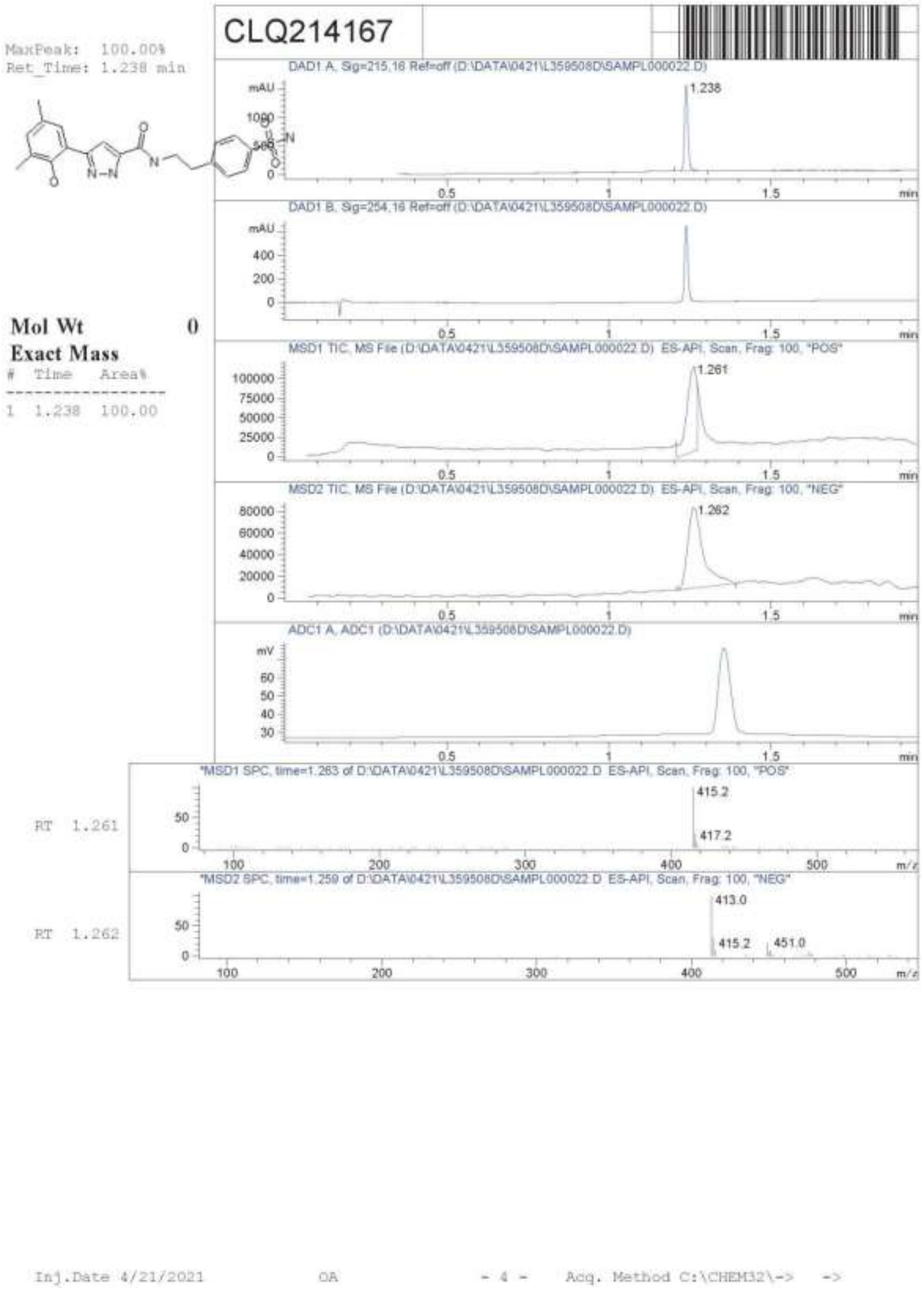

Figure S9D. LCMS spectrum of *N-{2-[4-(Aminosulfonyl)phenyl]ethyl}-3-(2-hydroxy-3,5-dimethylphenyl)-1H-pyrazole-5-carboxamide* (**5e**).

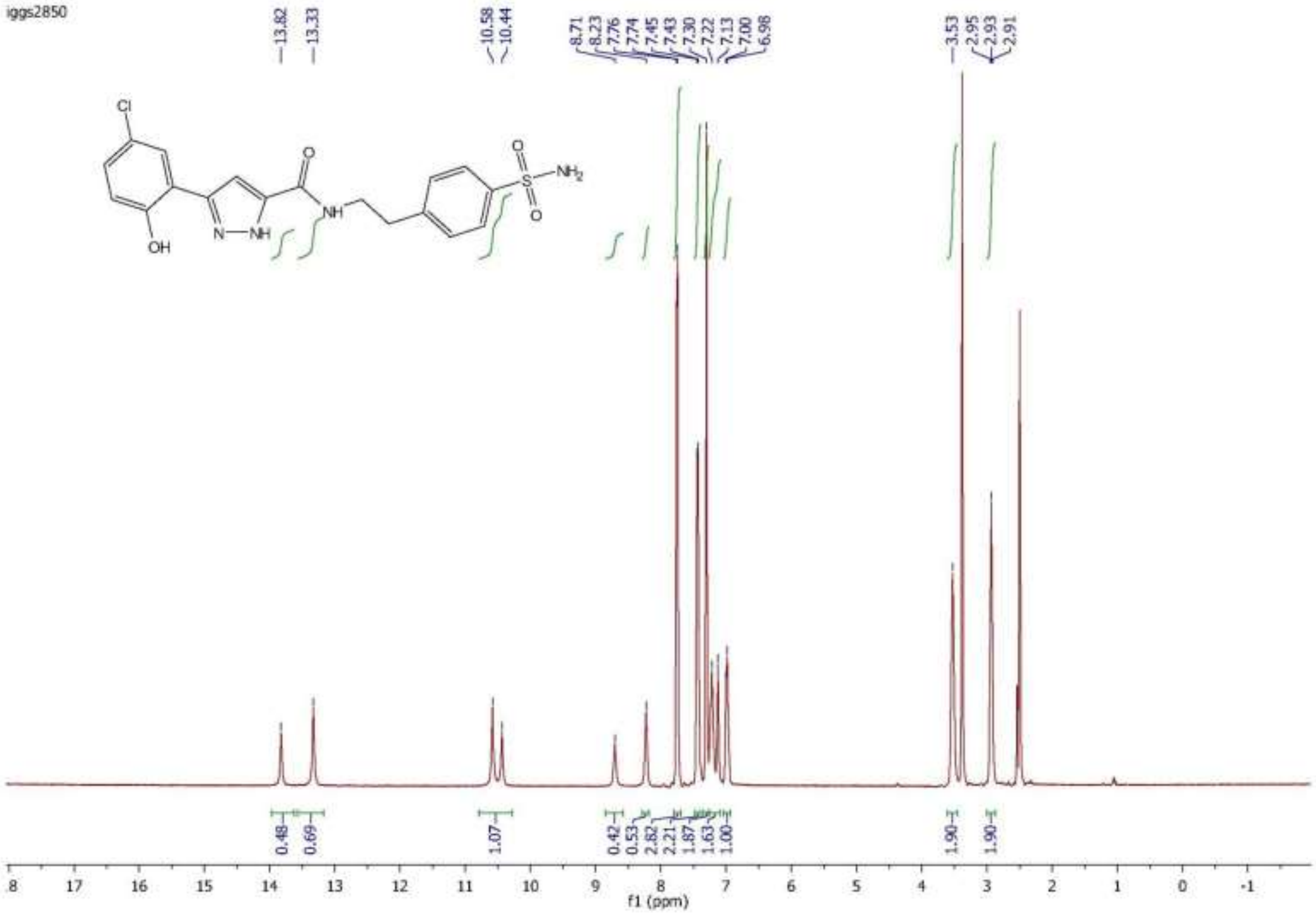

Figure S10A. ^{1}H NMR spectrum of *N-{2-[4-(Aminosulfonyl)phenyl]ethyl}-3-(5-chloro-2-hydroxyphenyl)-1H-pyrazole-5-carboxamide* (**5f)** in DMSO-*d6*.

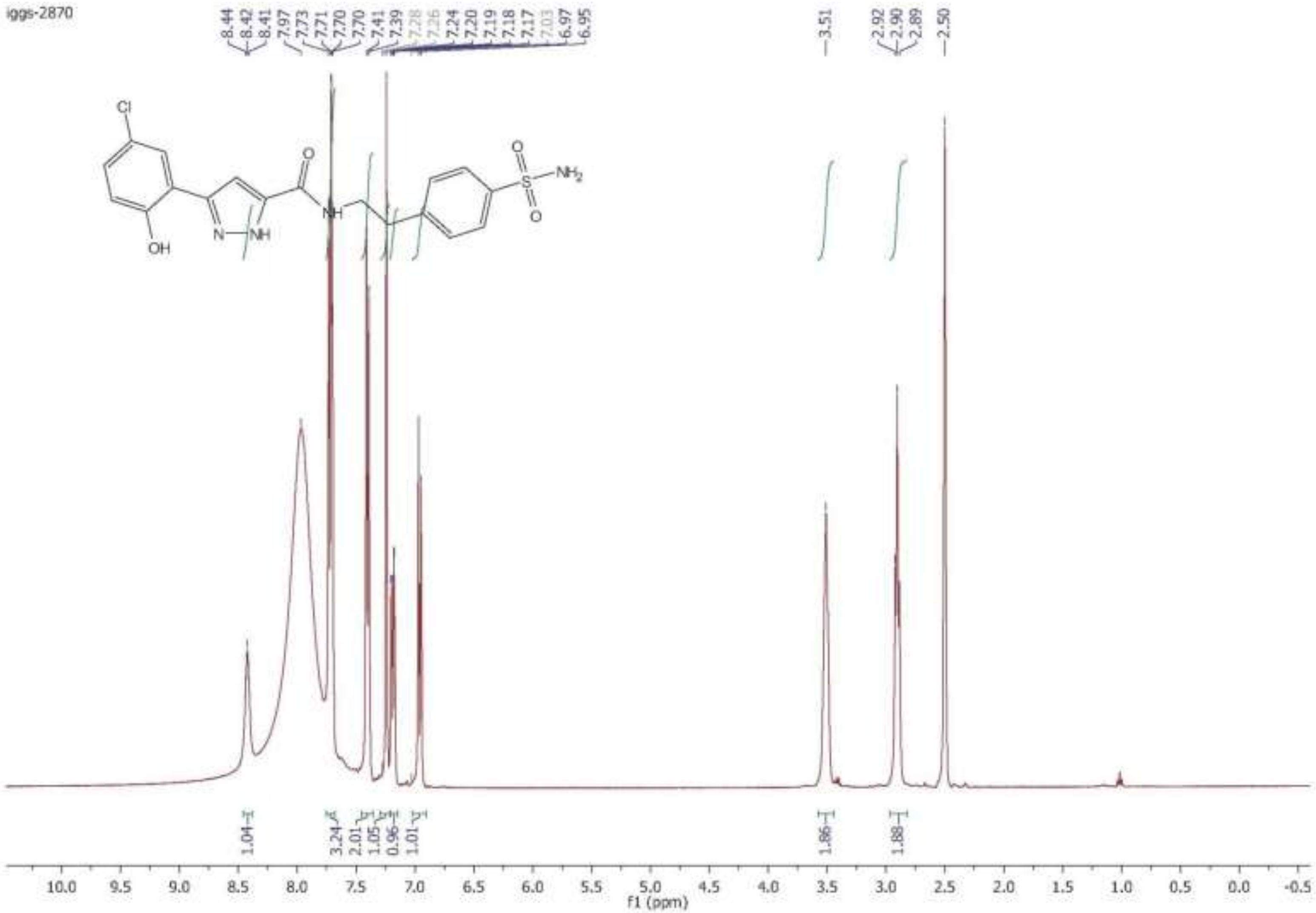

Figure S10B. ^{1}H NMR spectrum of *N-{2-[4-(Aminosulfonyl)phenyl]ethyl}-3-(5-chloro-2-hydroxyphenyl)-1H-pyrazole-5-carboxamide* (**5f)** in DMSO-*d6*+ 5%CF_3SO_3H.

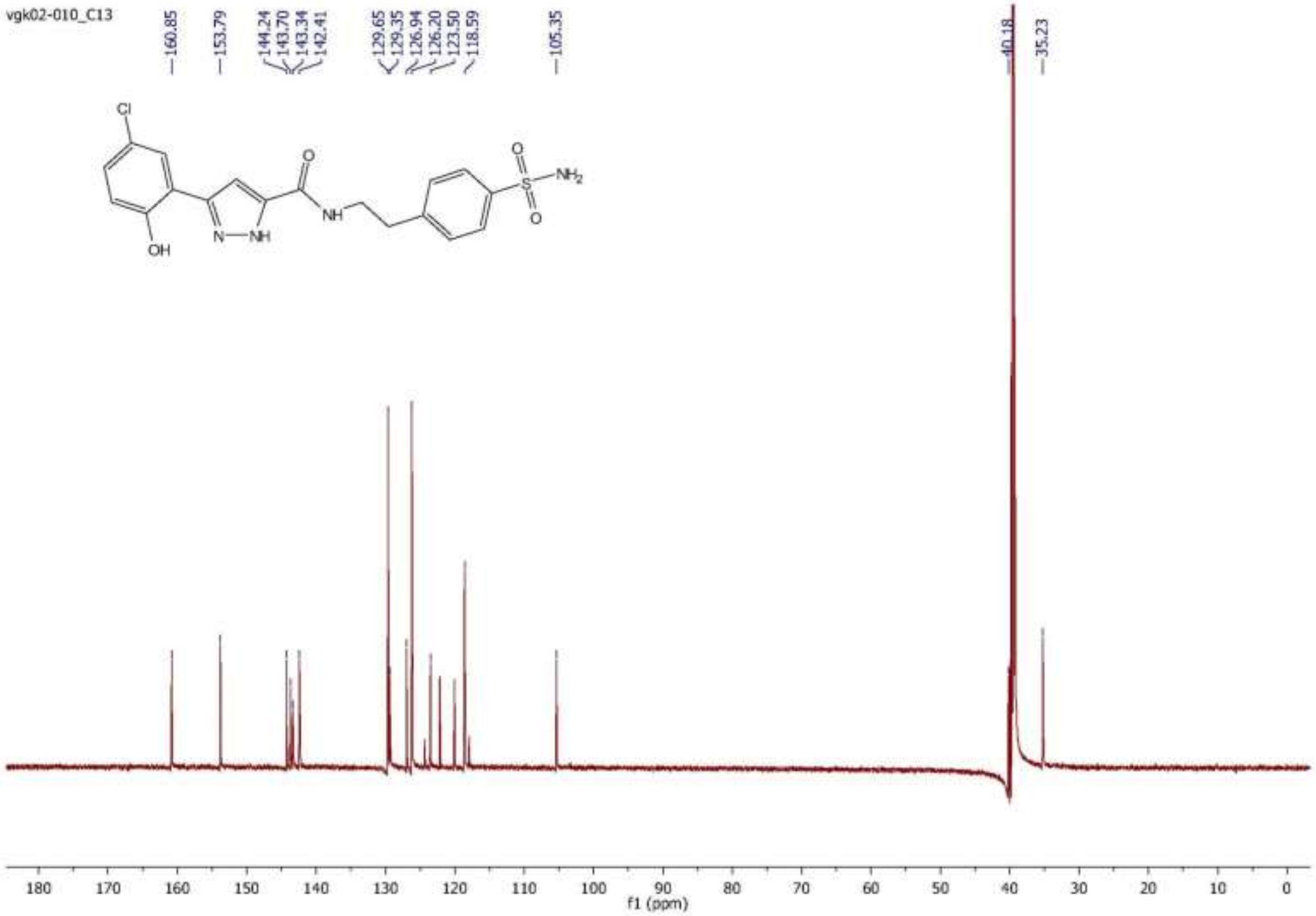

Figure S10C. ^{13}C NMR spectrum of *N-{2-[4-(Aminosulfonyl)phenyl]ethyl}-3-(5-chloro-2-hydroxyphenyl)-1H-pyrazole-5-carboxamide* (**5f)** in DMSO-*d6*+ 5%CF_3SO_3H.

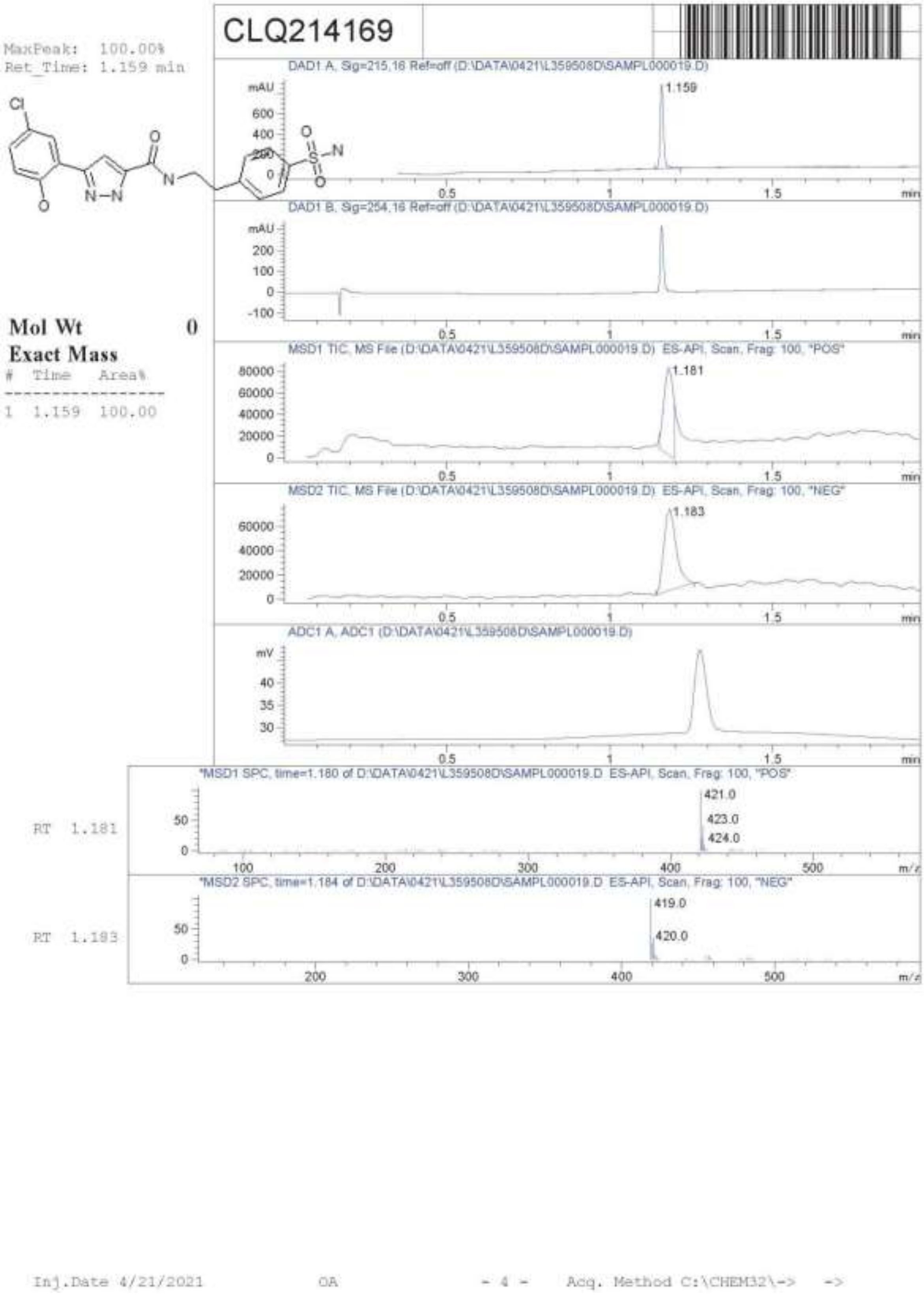

Figure S10D. LCMS spectrum of *N-{2-[4-(Aminosulfonyl)phenyl]ethyl}-3-(5-chloro-2-hydroxyphenyl)-1H-pyrazole-5-carboxamide* (**5f**).

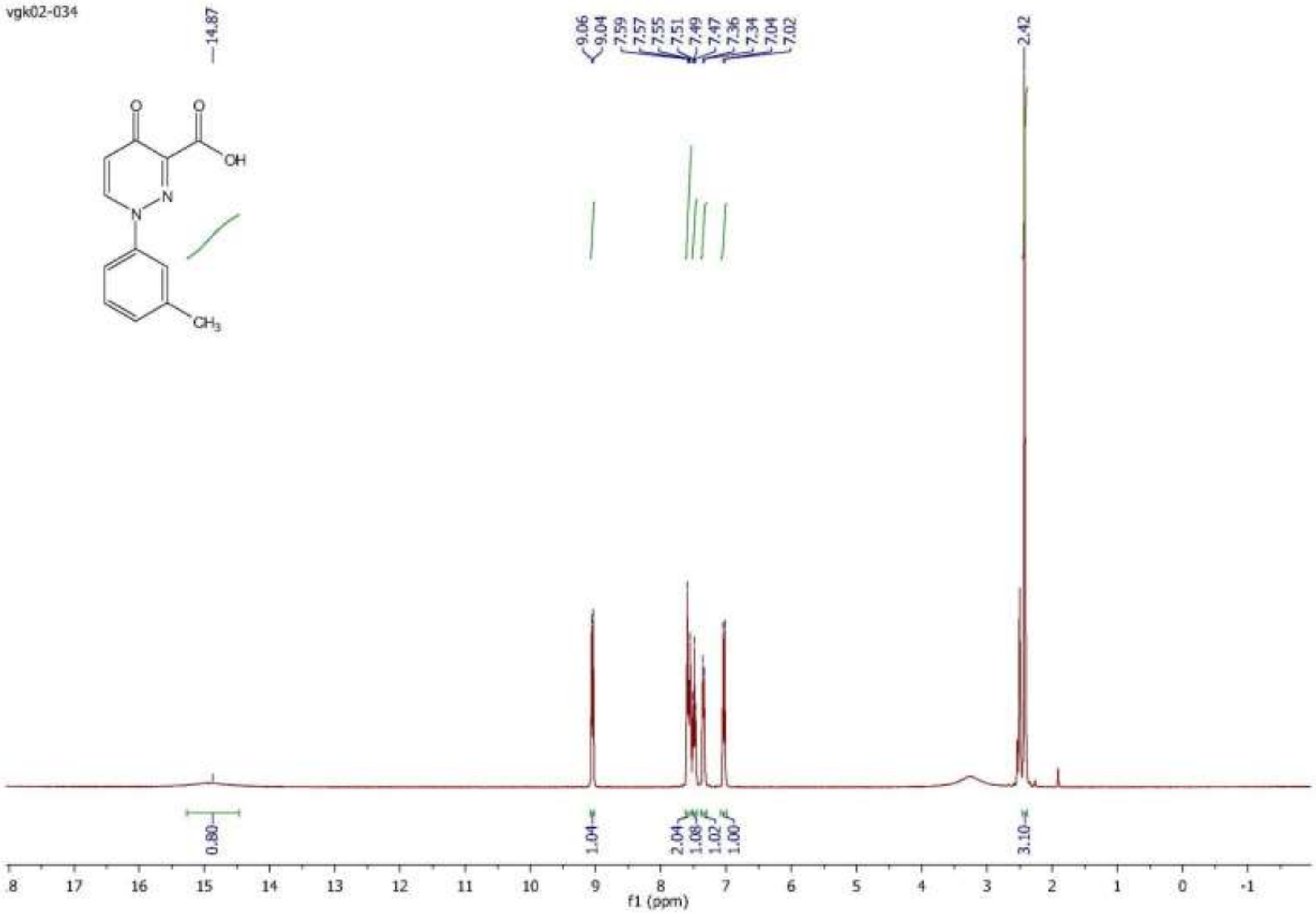

Figure S11. ¹H NMR spectrum of *1-(3-Methylphenyl)-4-oxo-1,4-dihydropyridazine-3-carboxylic acid* (**8a**) in DMSO-*d6*.

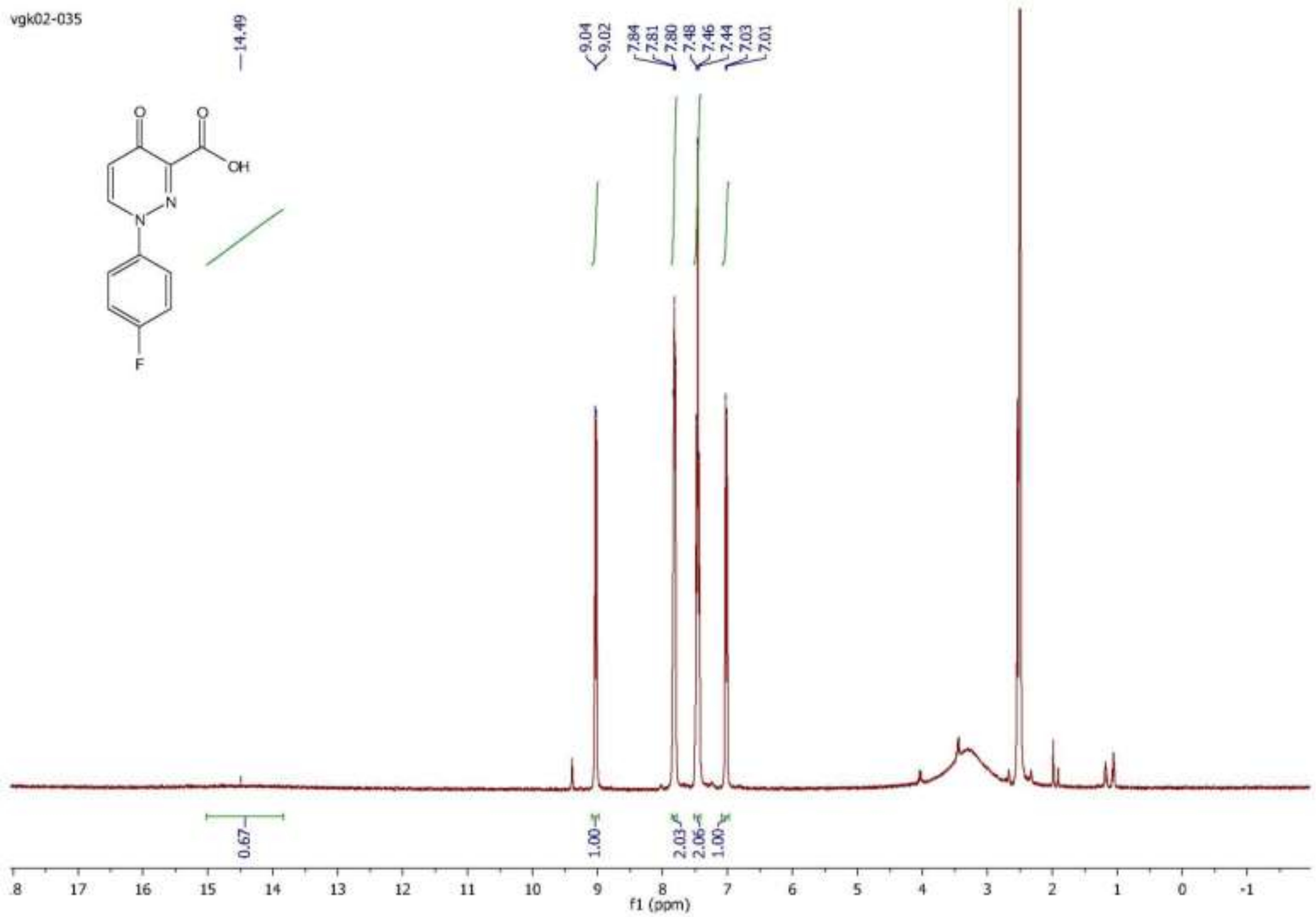

Figure S12. ^{1}H NMR spectrum of *1-(4-Fluorophenyl)-4-oxo-1,4-dihydropyridazine-3-carboxylic acid* (**8b**) in DMSO-*d6*.

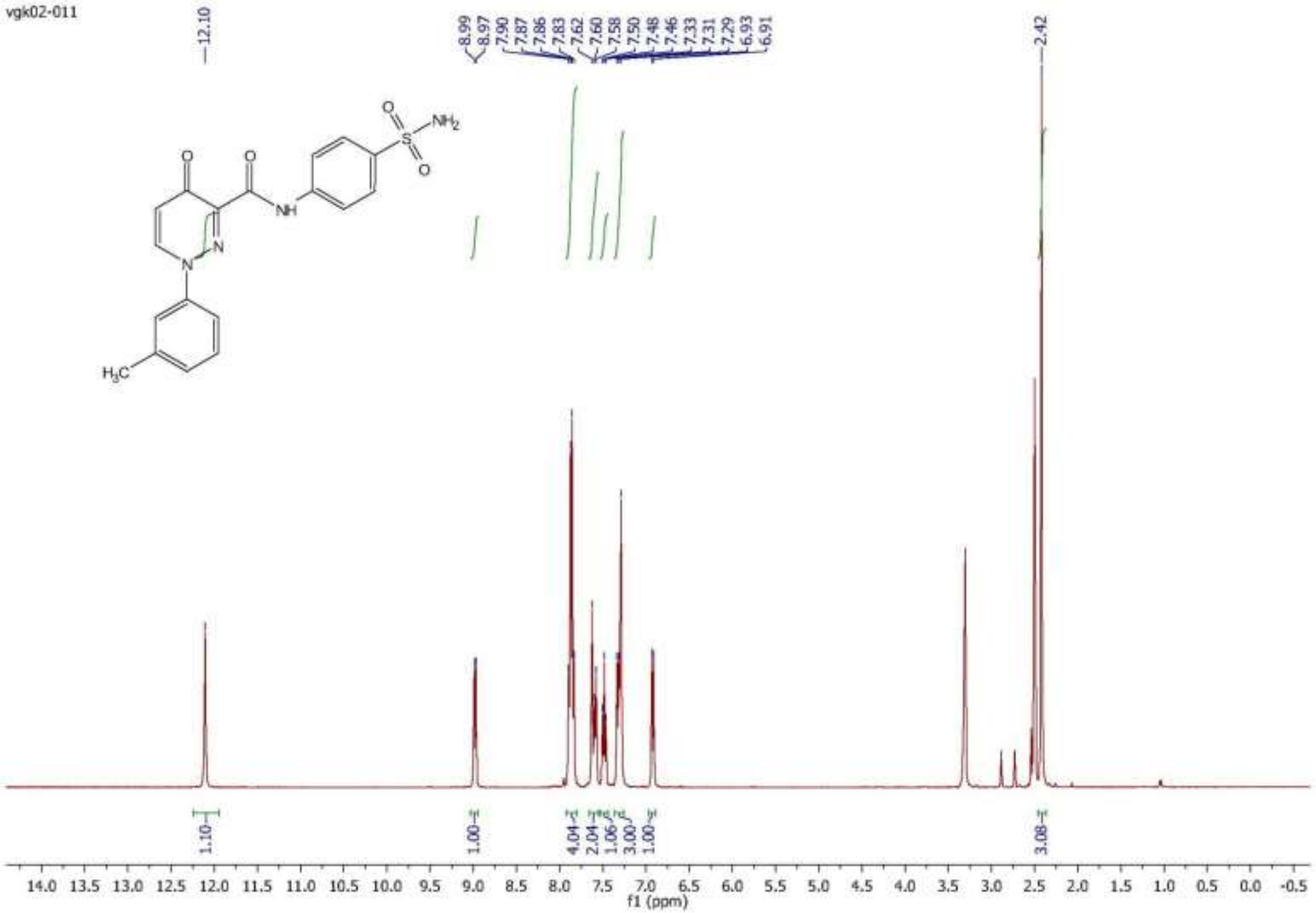

Figure S13A. ^{1}H NMR spectrum of *N-[4-(Aminosulfonyl)phenyl]-1-(3-methylphenyl)-4-oxo-1,4-dihydropyridazine-3-carboxamide* (**10a**) in DMSO-*d6*.

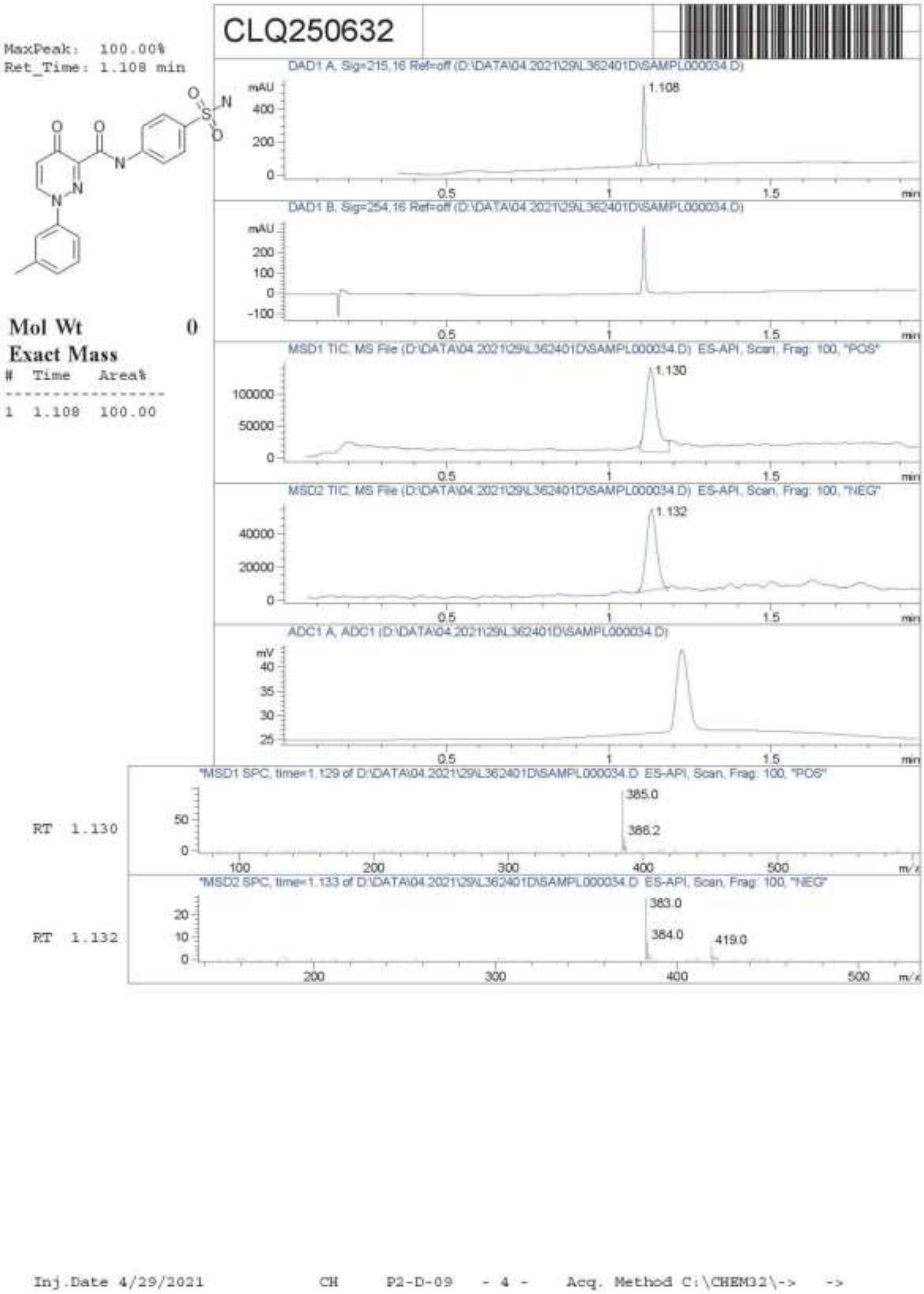

Figure S13B. LCMS spectrum of *N-[4-(Aminosulfonyl)phenyl]-1-(3-methylphenyl)-4-oxo-1,4-dihydropyridazine-3-carboxamide* (**10a).**

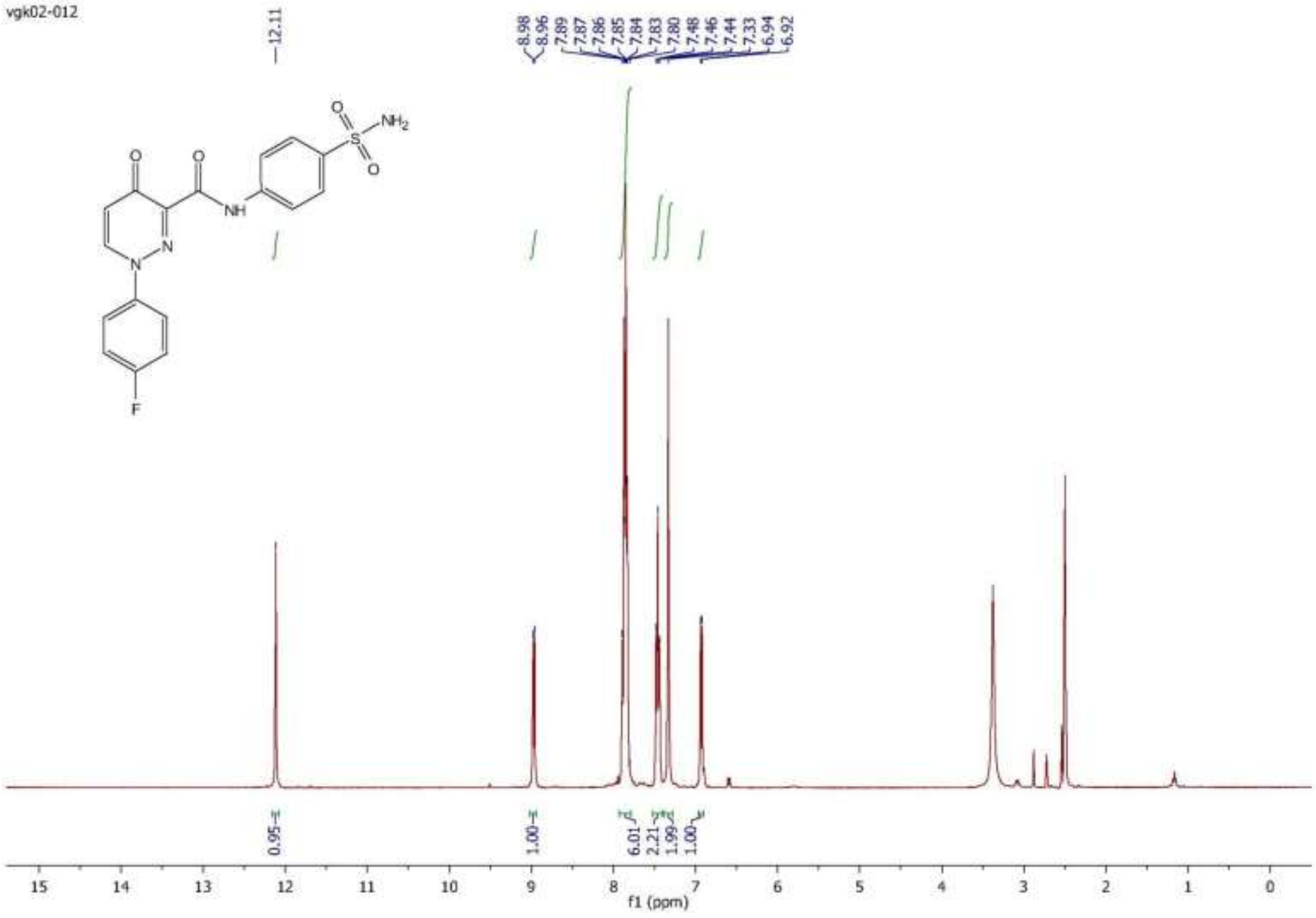

Figure S14A. ^{1}H NMR spectrum of *N-[4-(Aminosulfonyl)phenyl]-1-(4-fluorophenyl)-4-oxo-1,4-dihydropyridazine-3-carboxamide* (**10b**) in DMSO-*d6*.

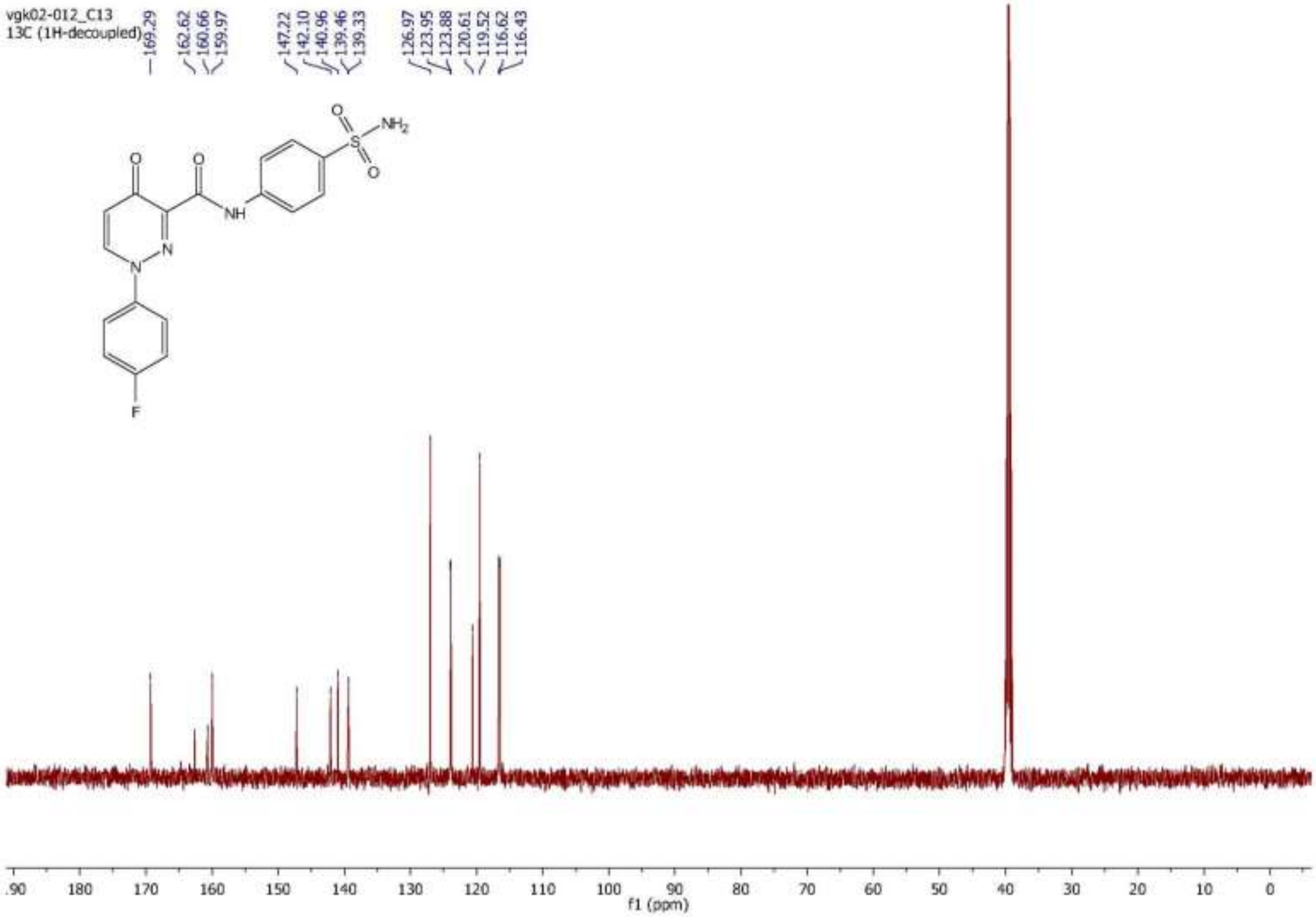

Figure S14B. ^{13}C NMR spectrum of *N-[4-(Aminosulfonyl)phenyl]-1-(4-fluorophenyl)-4-oxo-1,4-dihydropyridazine-3-carboxamide* (**10b**) in DMSO-*d6*.

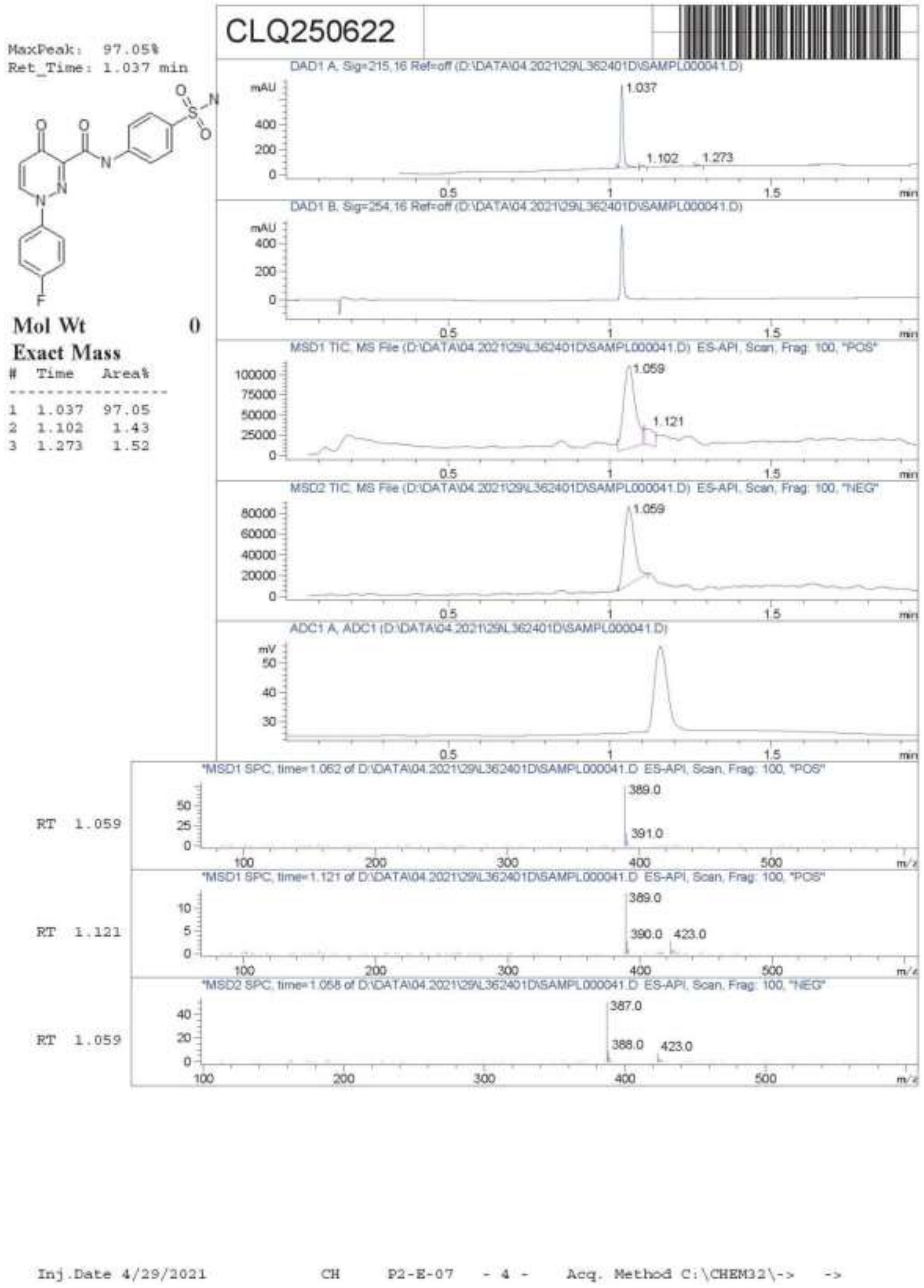

Figure S14C. LCMS spectrum of *N-[4-(Aminosulfonyl)phenyl]-1-(4-fluorophenyl)-4-oxo-1,4-dihydropyridazine-3-carboxamide* (**10b**).

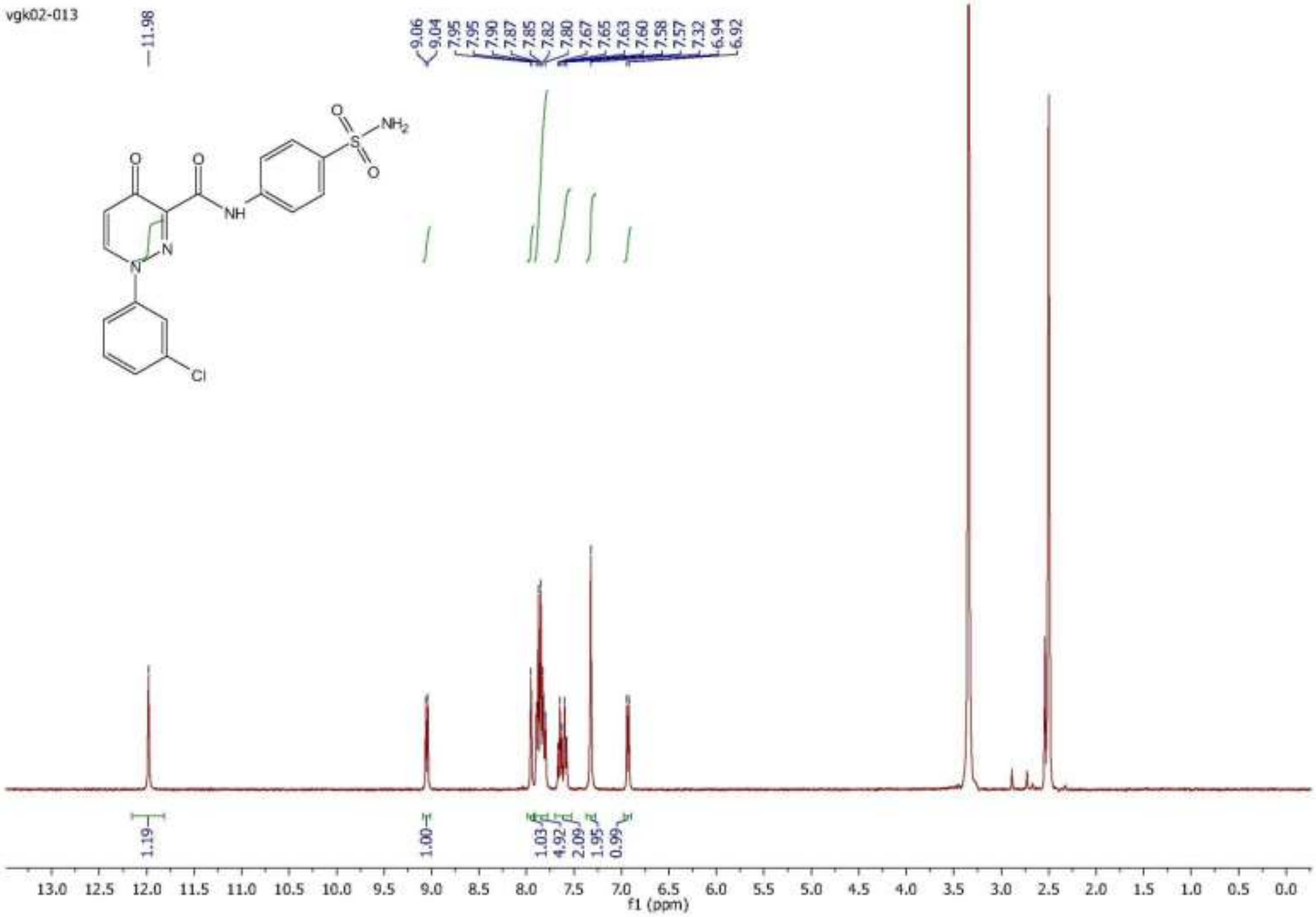

Figure S15A. ^{1}H NMR spectrum of *N-[4-(Aminosulfonyl)phenyl]-1-(3-chlorophenyl)-4-oxo-1,4-dihydropyridazine-3-carboxamide* (**10c**) in DMSO-*d6*.

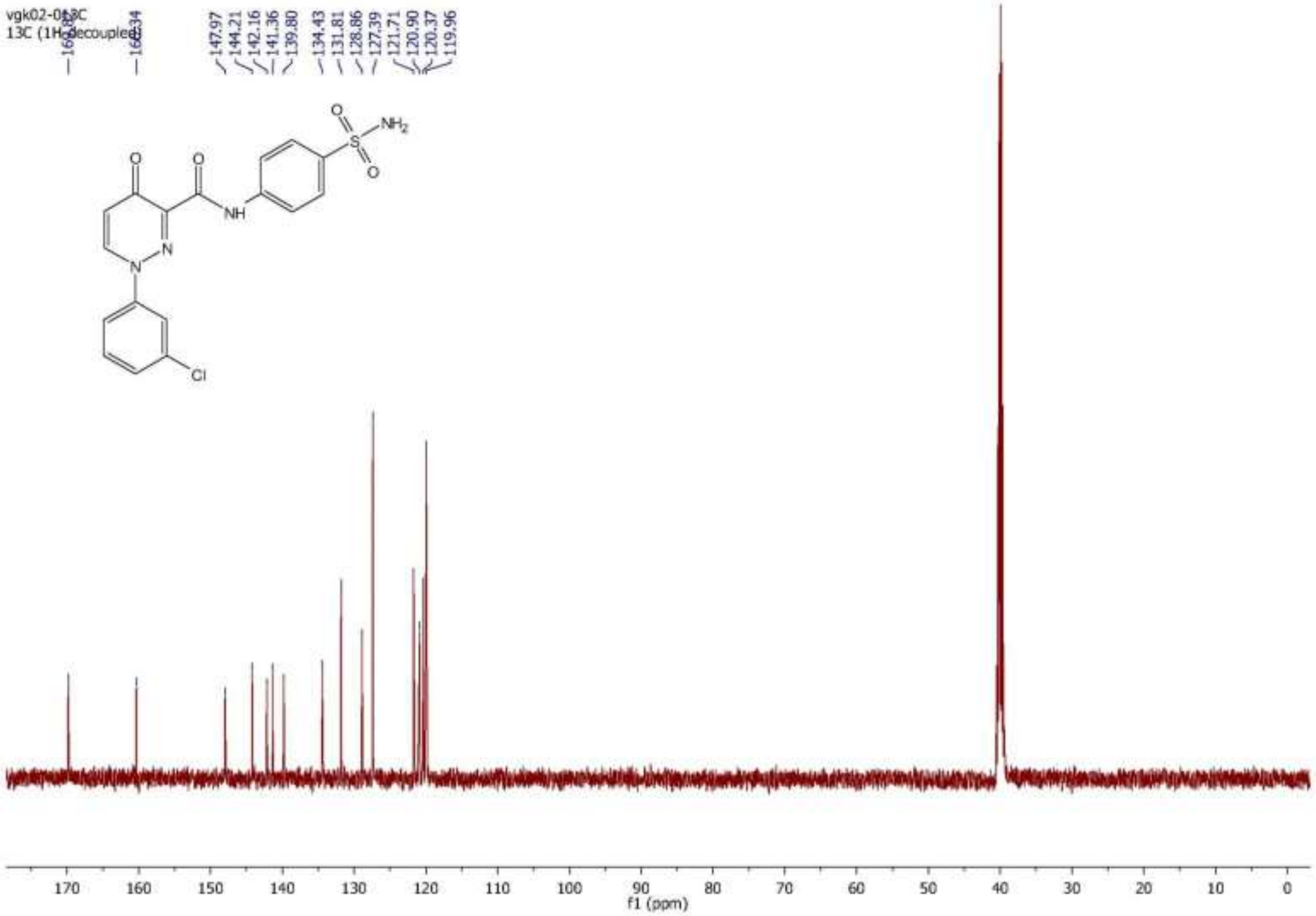

Figure S15B. ^{13}C NMR spectrum of *N-[4-(Aminosulfonyl)phenyl]-1-(3-chlorophenyl)-4-oxo-1,4-dihydropyridazine-3-carboxamide* (**10c**) in DMSO-*d6*.

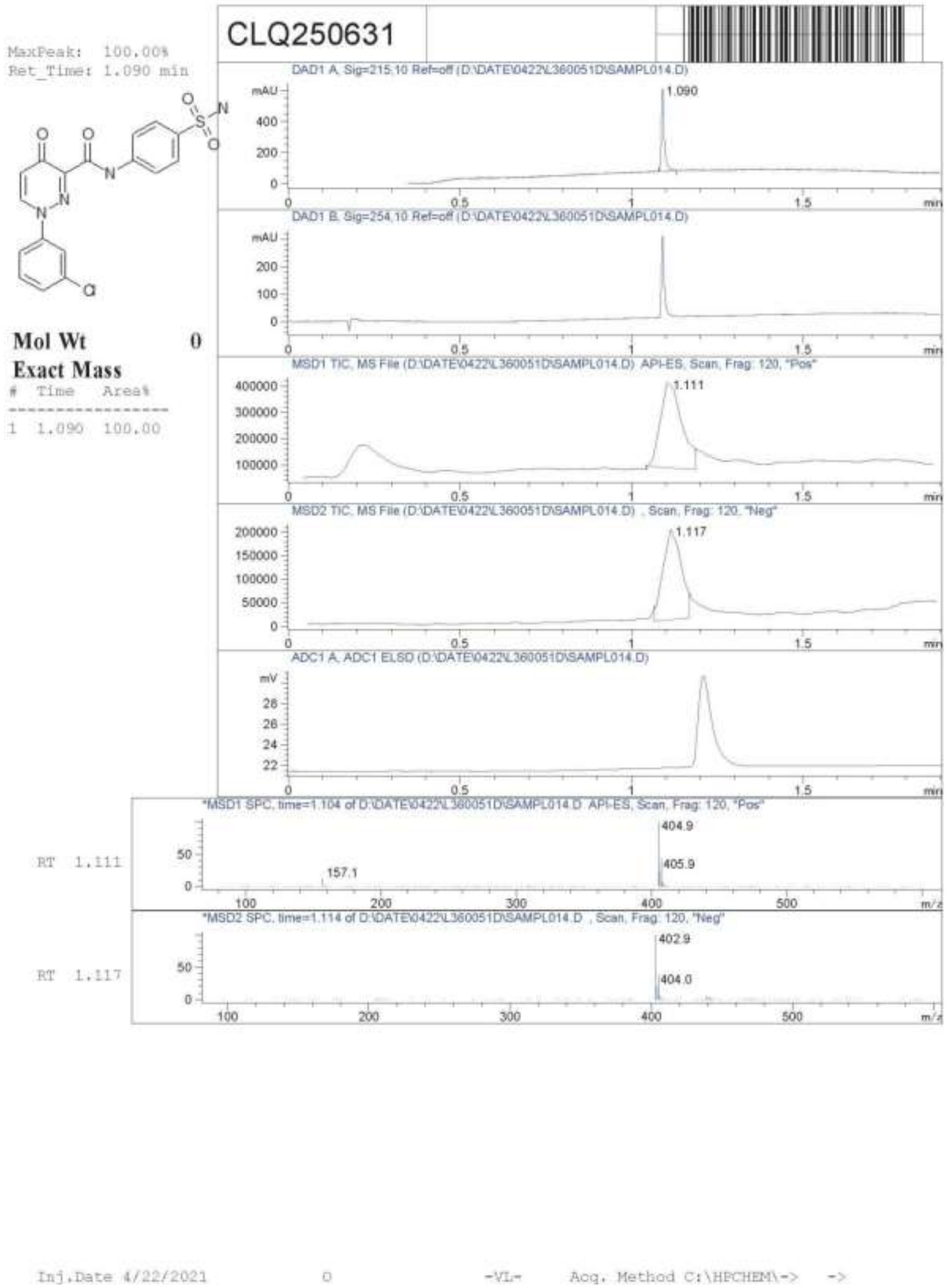

Figure S15C. LCMS spectrum of *N-[4-(Aminosulfonyl)phenyl]-1-(3-chlorophenyl)-4-oxo-1,4-dihydropyridazine-3-carboxamide* (**10c**).

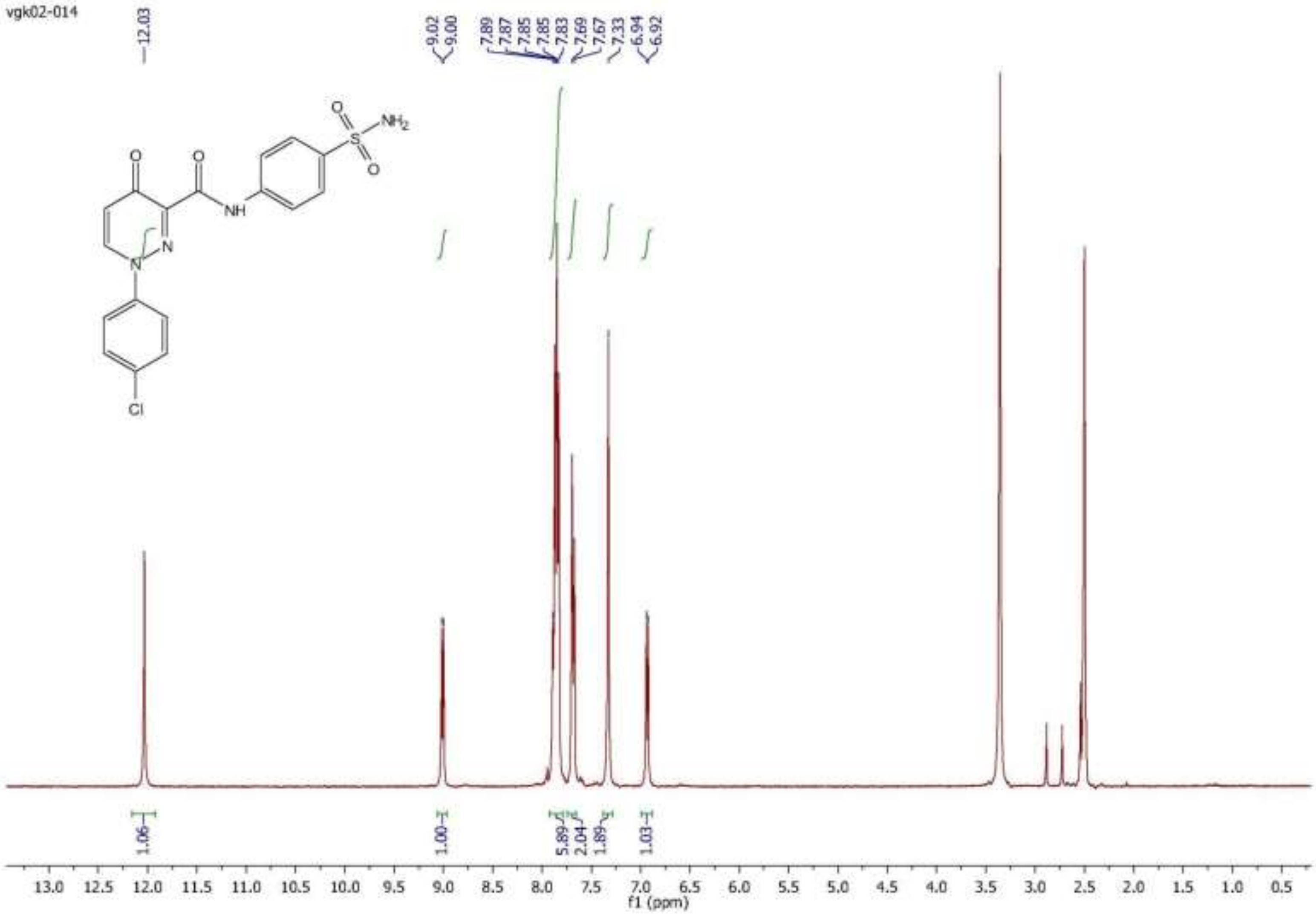

Figure S16A. ^{1}H NMR spectrum of *N-[4-(Aminosulfonyl)phenyl]-1-(4-chlorophenyl)-4-oxo-1,4-dihydropyridazine-3-carboxamide* (**10d**). in DMSO-*d6*.

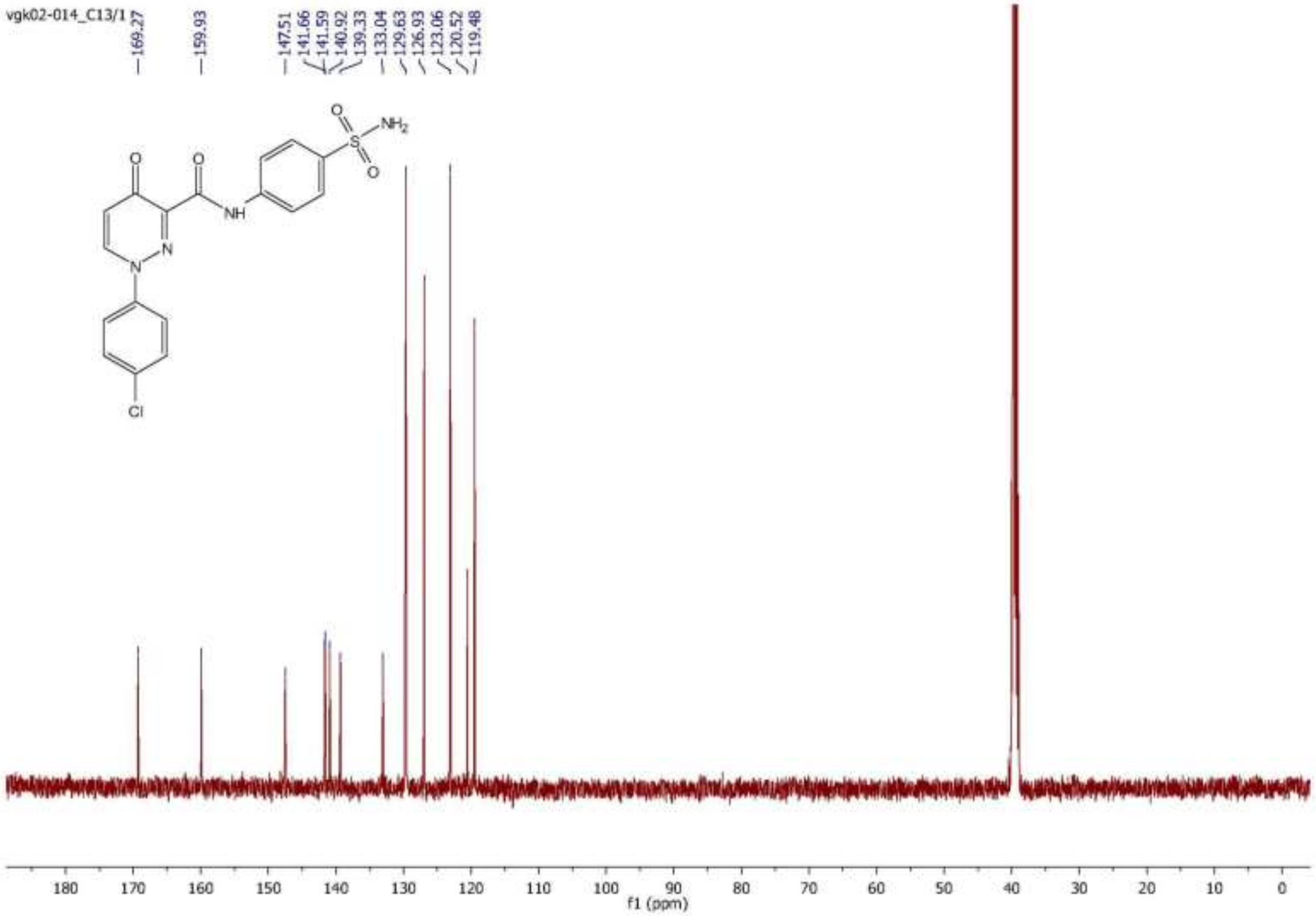

Figure S16B. ^{13}C NMR spectrum of *N-[4-(Aminosulfonyl)phenyl]-1-(4-chlorophenyl)-4-oxo-1,4-dihydropyridazine-3-carboxamide* (**10d**). in DMSO-*d6*.

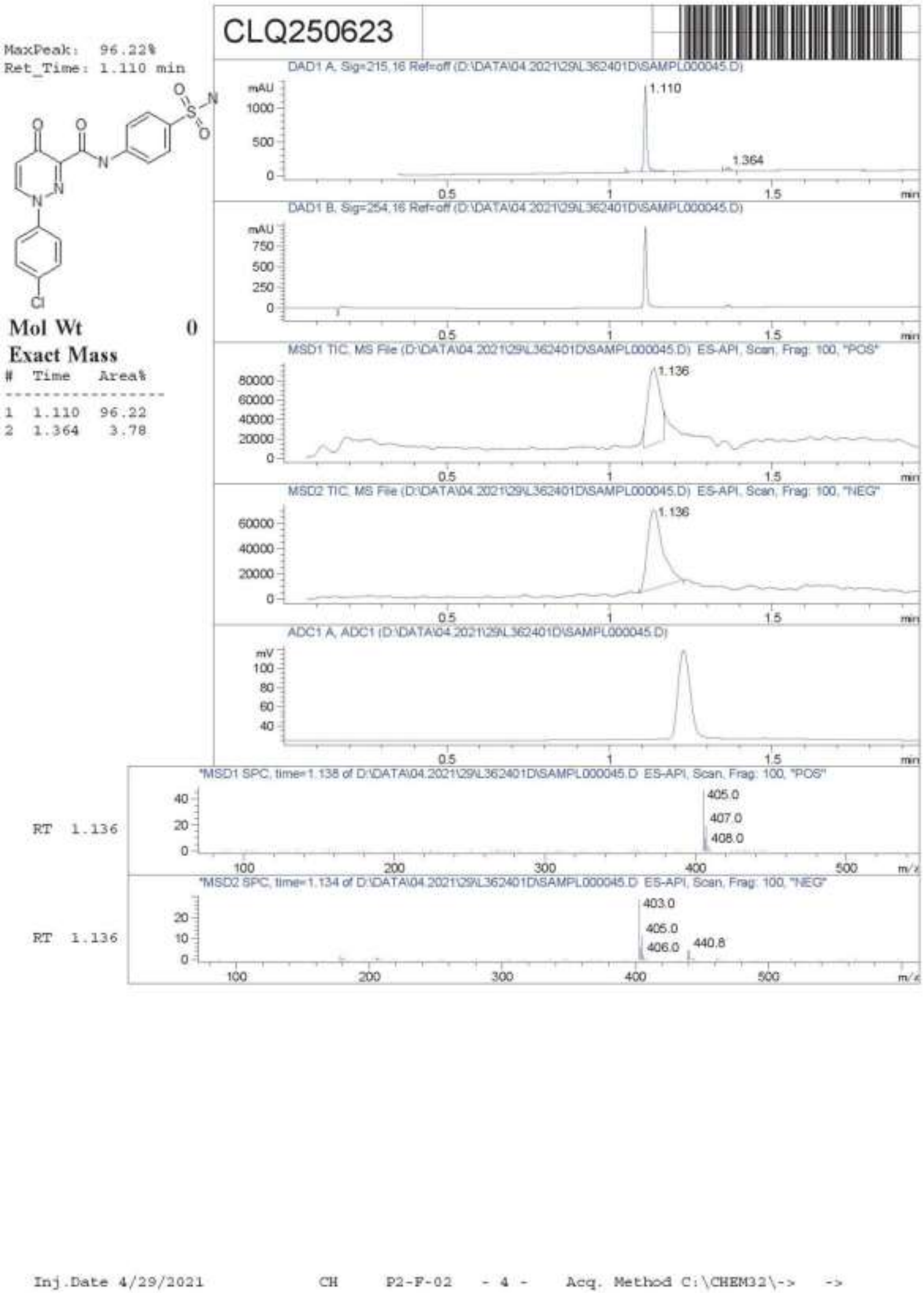

Figure S16C. LCMS spectrum of *N-[4-(Aminosulfonyl)phenyl]-1-(4-chlorophenyl)-4-oxo-1,4-dihydropyridazine-3-carboxamide* (**10d**).

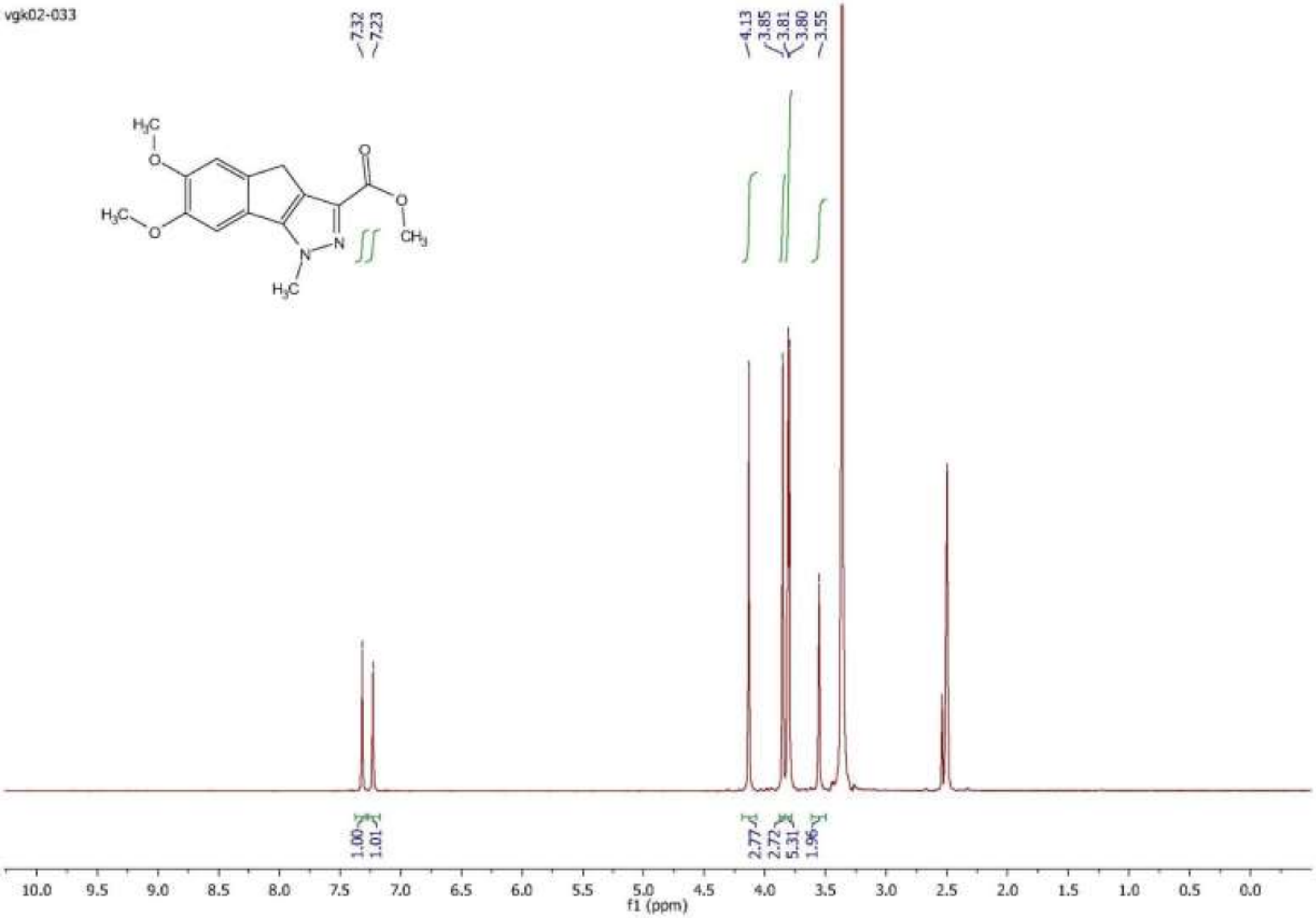

Figure S17A. ^{1}H NMR spectrum of *Methyl 6,7-dimethoxy-1-methyl-1,4-dihydroindeno[1,2-c]pyrazole-3-carboxylate* (**13**) in DMSO-*d6*.

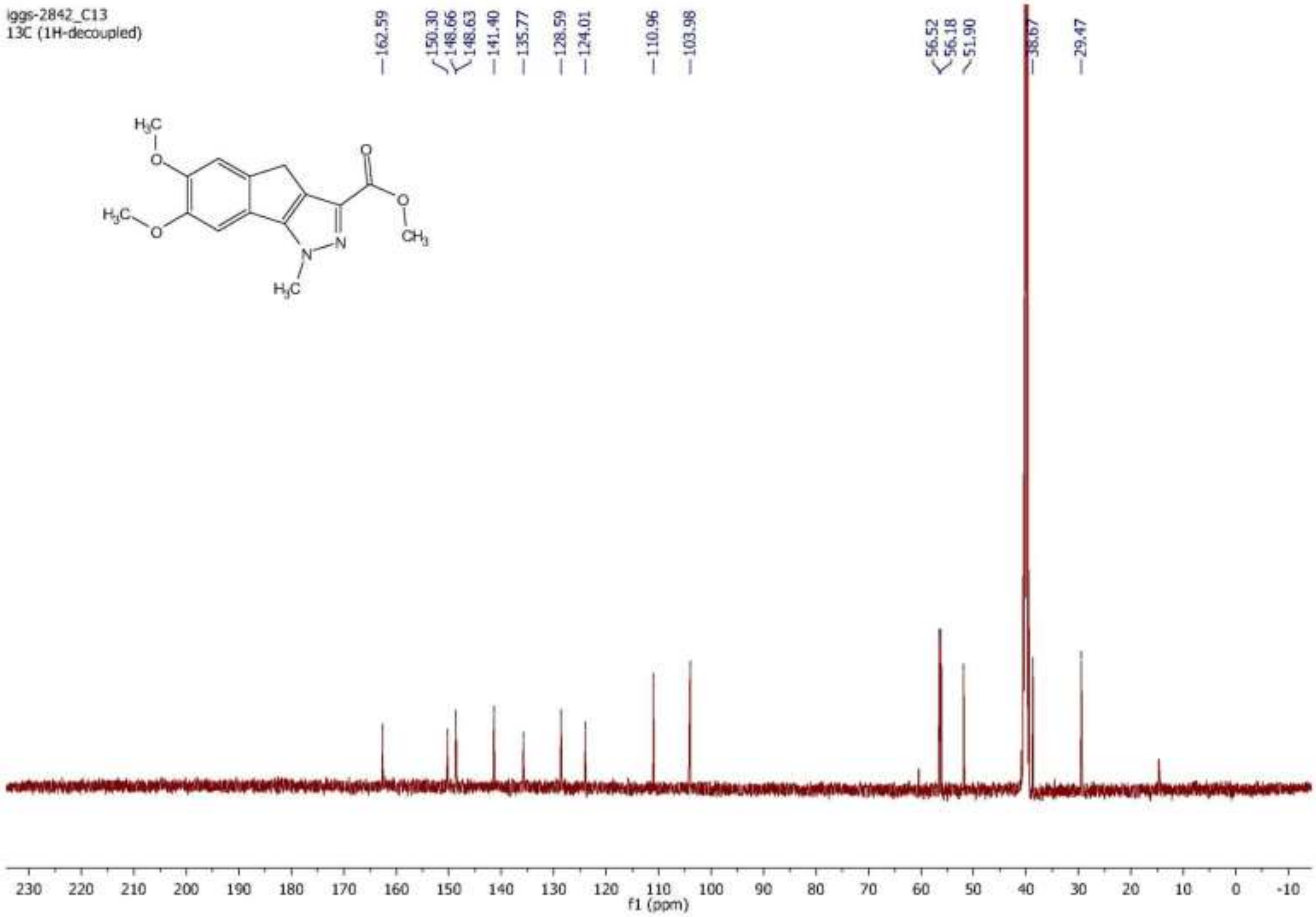

Figure S17B. ^{13}C NMR spectrum of *Methyl 6,7-dimethoxy-1-methyl-1,4-dihydroindeno[1,2-c]pyrazole-3-carboxylate* (**13**) in DMSO-*d6*.

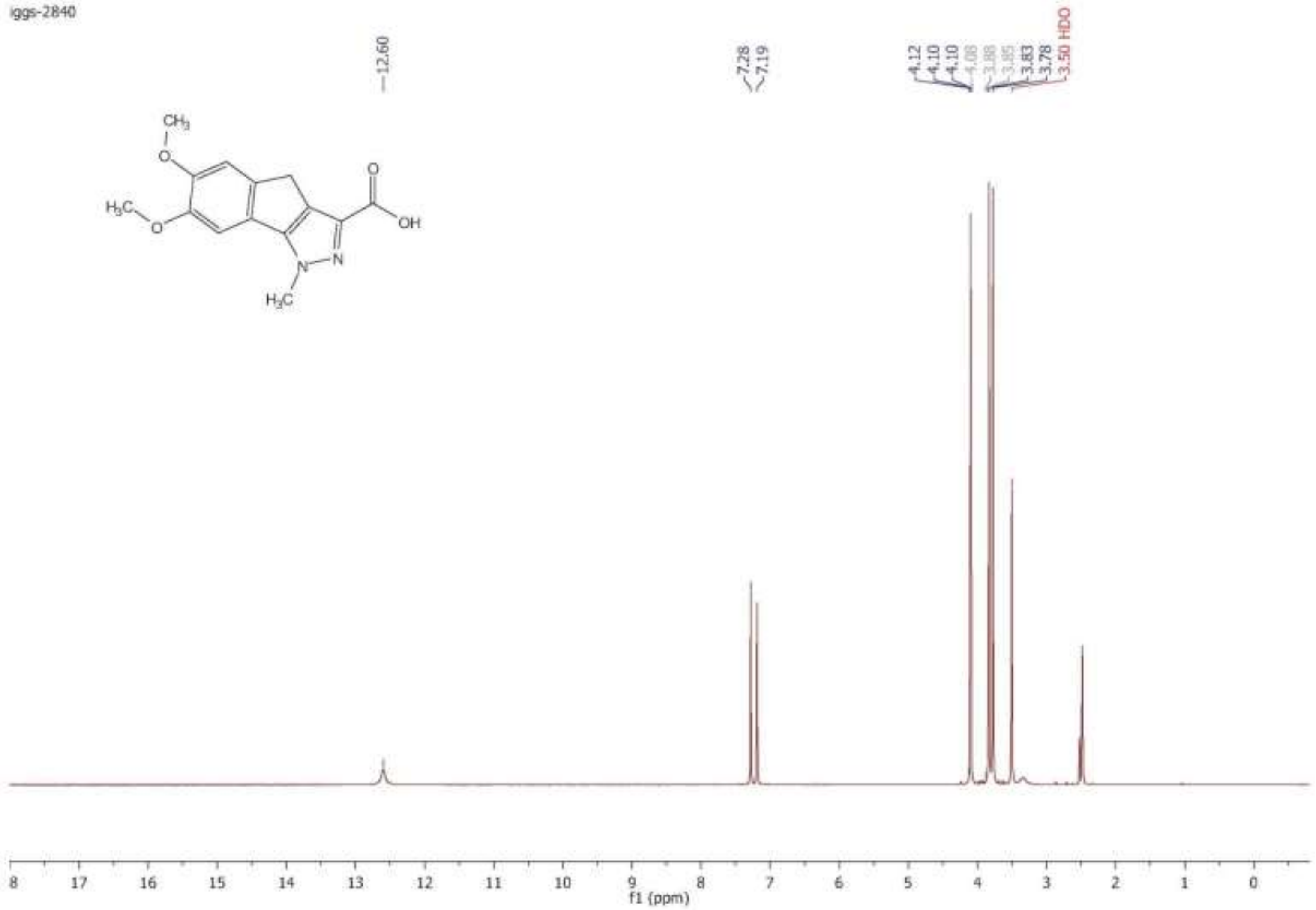

1

Figure S18A. [1]H.NMR spectrum of *6,7-Dimethoxy-1-methyl-1,4-dihydroindeno[1,2-c]pyrazole-3-carboxylic acid* (**14**). in DMSO-*d6*.

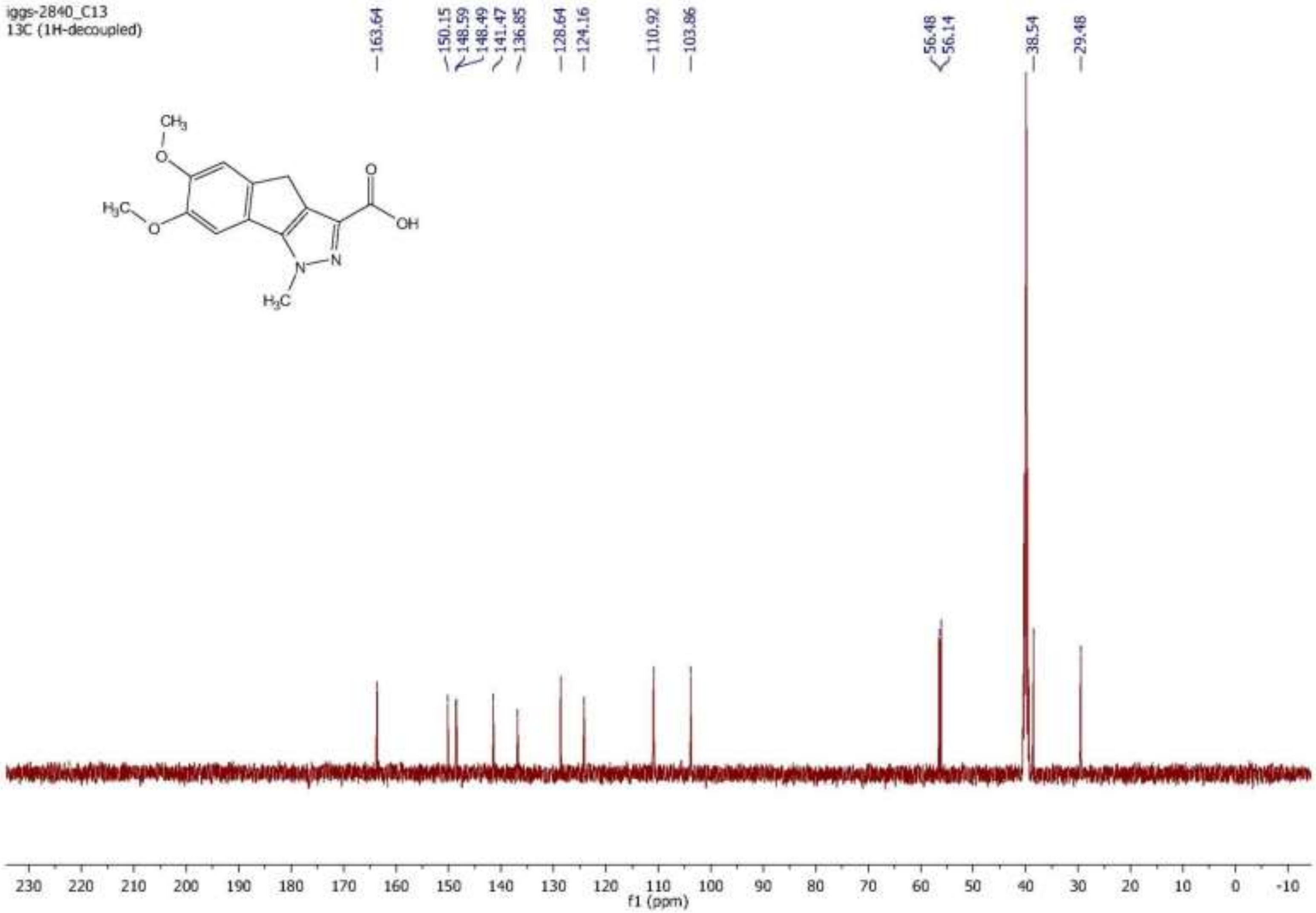

Figure S18B. ^{13}C.NMR spectrum of *6,7-Dimethoxy-1-methyl-1,4-dihydroindeno[1,2-c]pyrazole-3-carboxylic acid* (**14**). in DMSO-*d6*.

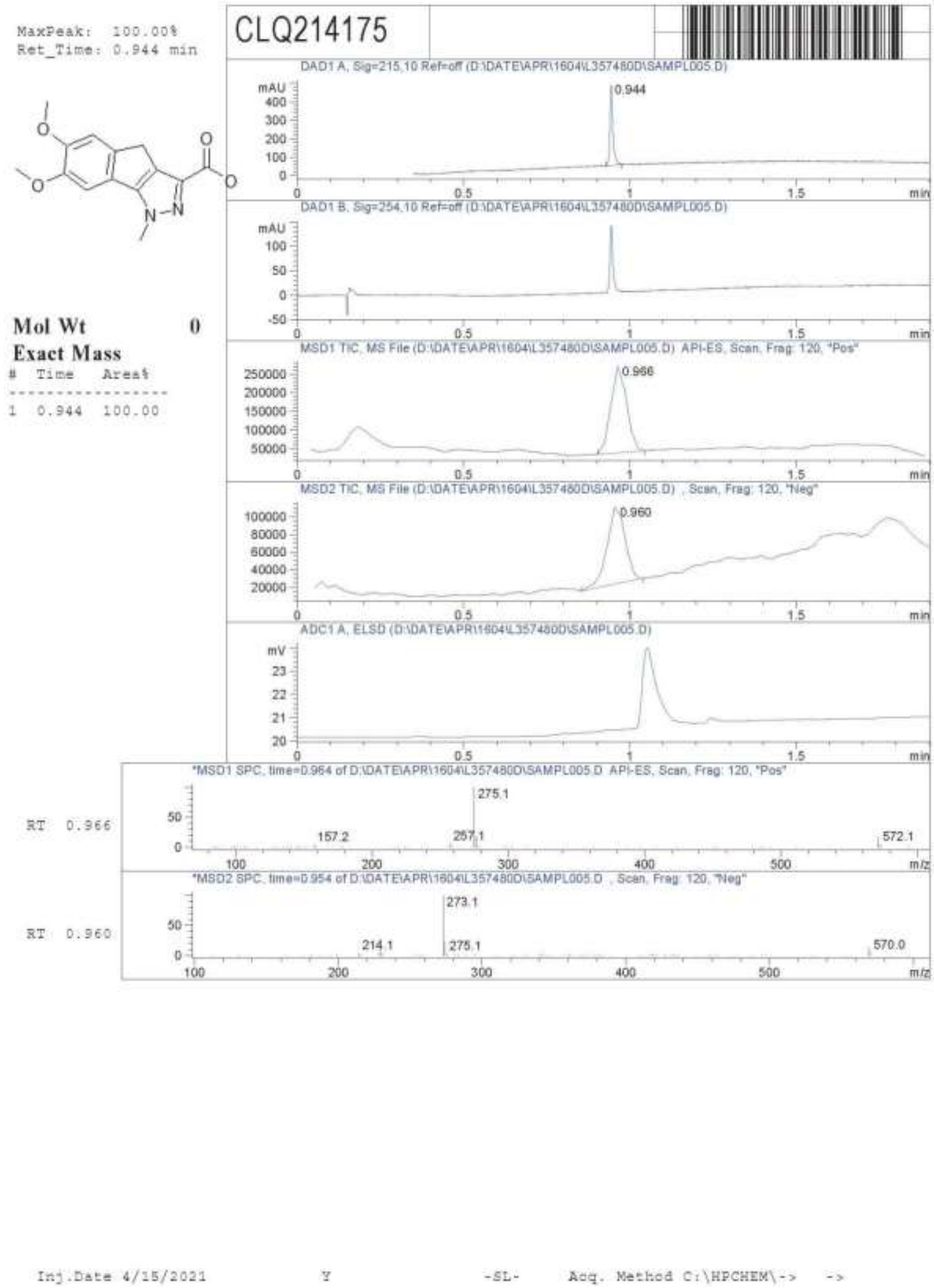

Figure S18C. LCMS spectrum of *6,7-Dimethoxy-1-methyl-1,4-dihydroindeno[1,2-c]pyrazole-3-carboxylic acid* (**14**).

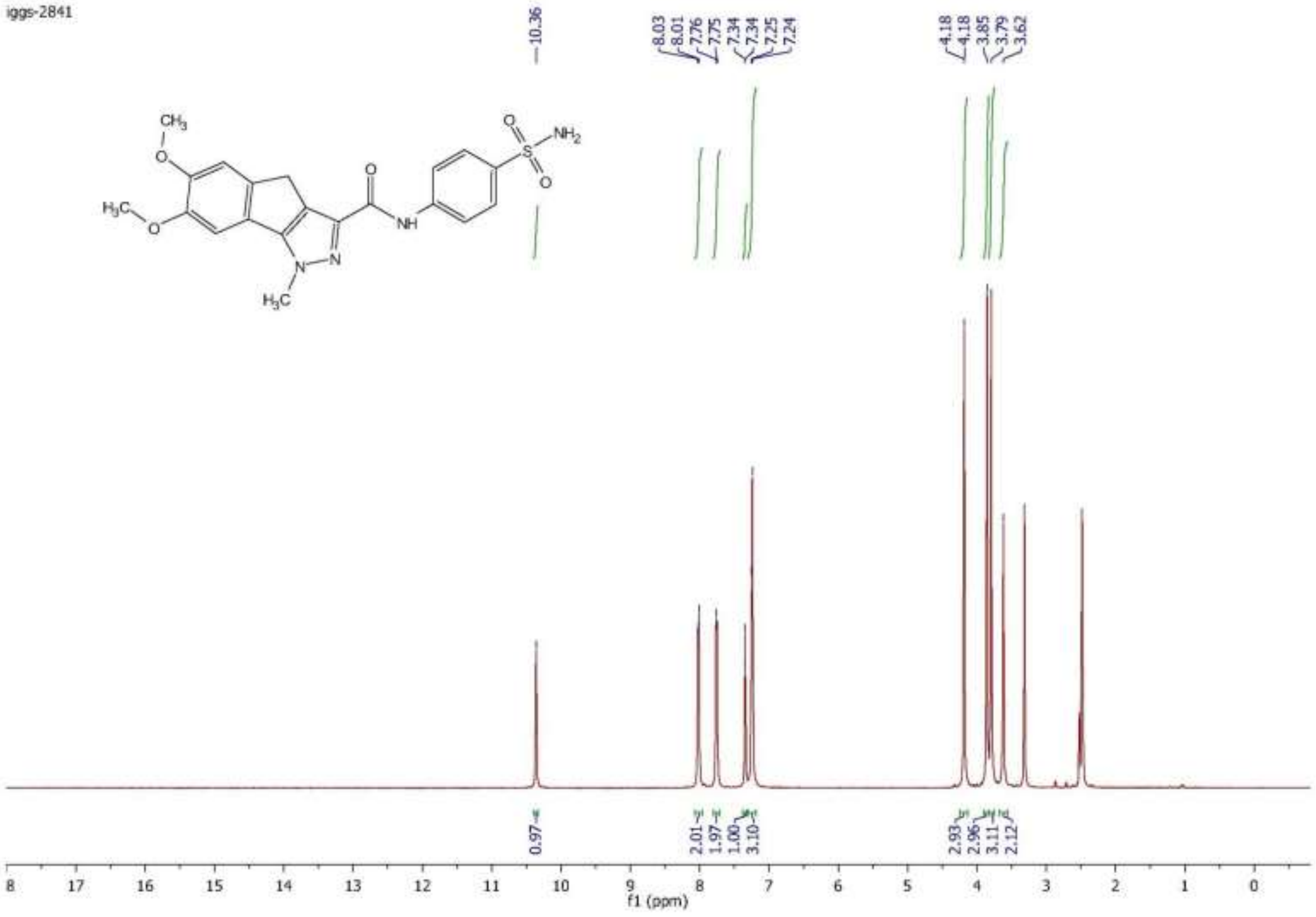

Figure S19A. ^{1}H.NMR spectrum of *N-[4-(Aminosulfonyl)phenyl]-6,7-dimethoxy-1-methyl-1,4-dihydroindeno[1,2-c]pyrazole-3-carboxamide* (**15**) in DMSO-*d6*.

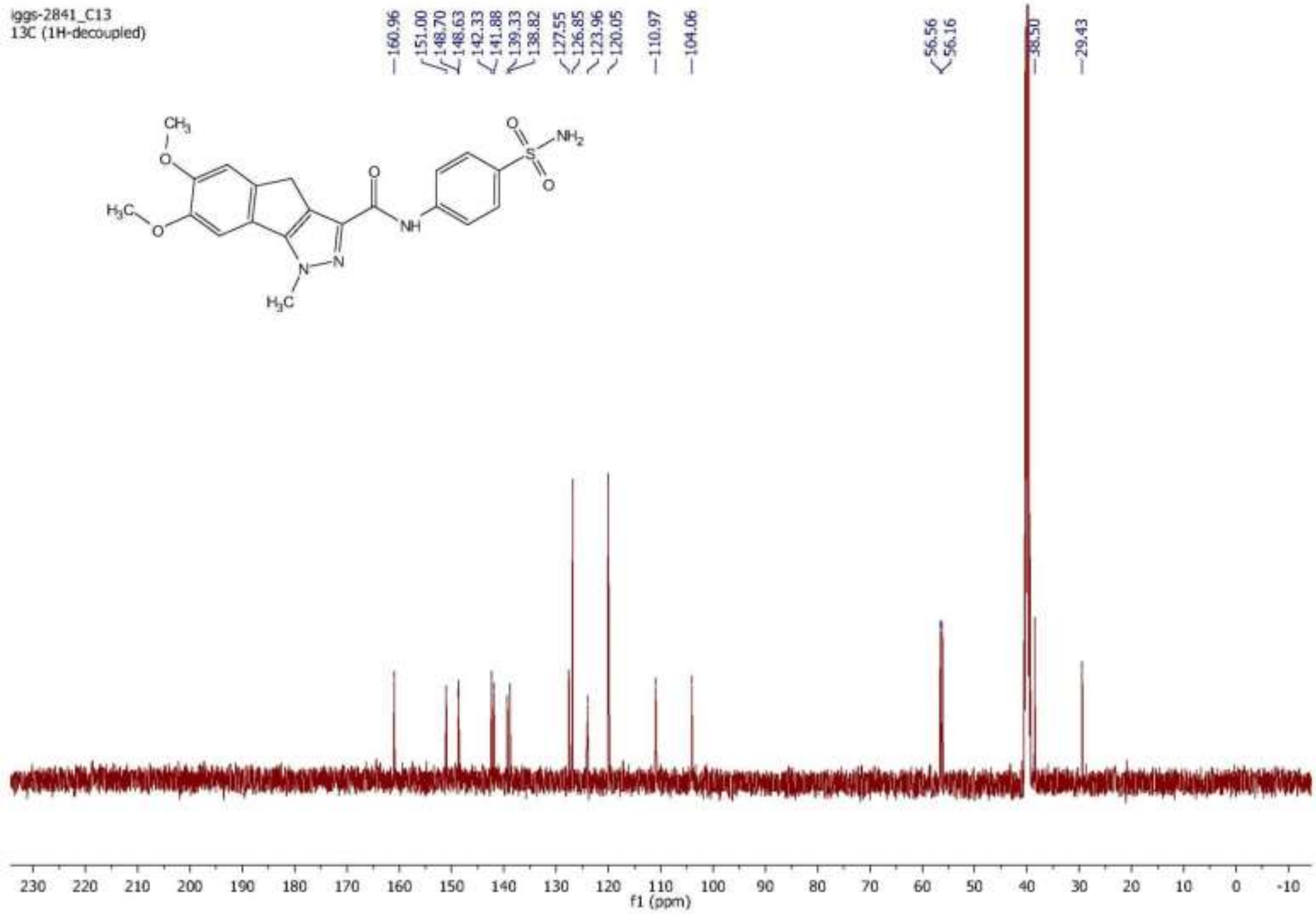

Figure S19B. ^{13}C.NMR spectrum of *N-[4-(Aminosulfonyl)phenyl]-6,7-dimethoxy-1-methyl-1,4-dihydroindeno[1,2-c]pyrazole-3-carboxamide* (**15**) in DMSO-*d6*.

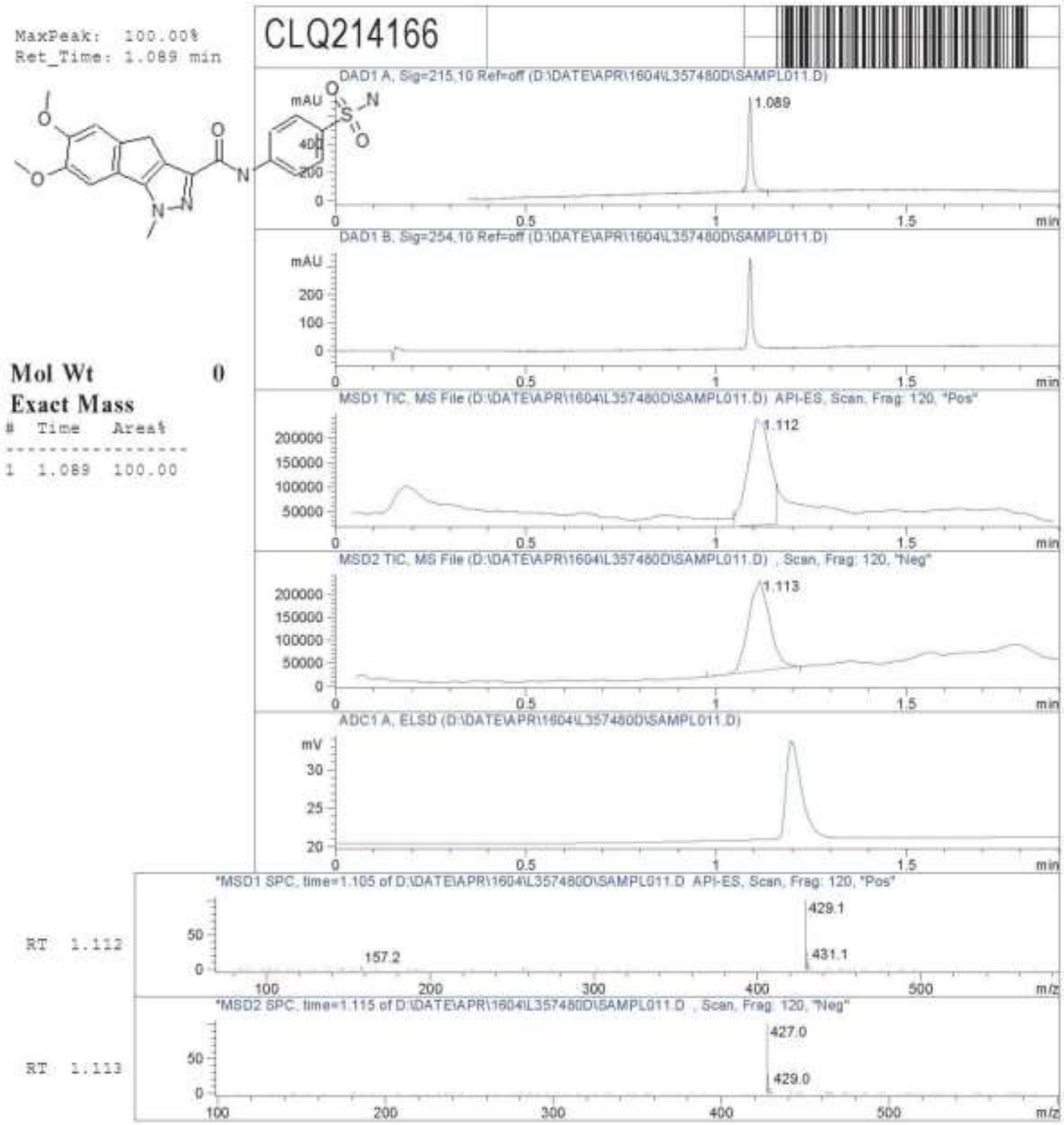

Inj.Date 4/15/2021 Y -SL- Acq. Method C:\HPCHEM\-> ->

Figure S19C. LCMS spectrum of *N-[4-(Aminosulfonyl)phenyl]-6,7-dimethoxy-1-methyl-1,4-dihydroindeno[1,2-c]pyrazole-3-carboxamide* (**15**).
